# Mitoribosomal synthetic lethality overcomes multidrug resistance in MYC-driven neuroblastoma

**DOI:** 10.1038/s41419-023-06278-x

**Published:** 2023-11-16

**Authors:** Karolina Borankova, Maria Krchniakova, Lionel Y. W. Leck, Adela Kubistova, Jakub Neradil, Patric J. Jansson, Michael D. Hogarty, Jan Skoda

**Affiliations:** 1 Department of Experimental Biology, Faculty of Science, Masaryk University, 62500 Brno, Czech Republic; 2 International Clinical Research Center, St. Anne’s University Hospital, 65691 Brno, Czech Republic; 3 Cancer Drug Resistance & Stem Cell Program, School of Medical Science, Faculty of Medicine and Health, The University of Sydney, Camperdown, NSW 2006 Australia; 4 Bill Walsh Translational Cancer Research Laboratory, Kolling Institute, Faculty of Medicine and Health, The University of Sydney, St. Leonards, NSW 2065 Australia; 5 Division of Oncology and Center for Childhood Cancer Research, The Children’s Hospital of Philadelphia, Philadelphia, PA USA; 6 Department of Pediatrics, Perelman School of Medicine, University of Pennsylvania, Philadelphia, PA USA

**Keywords:** Paediatric cancer, Target identification, Oncogenes

## Abstract

Mitochondria are central for cancer responses to therapy-induced stress signals. Refractory tumors often show attenuated sensitivity to apoptotic signaling, yet clinically relevant molecular actors to target mitochondria-mediated resistance remain elusive. Here, we show that MYC-driven neuroblastoma cells rely on intact mitochondrial ribosome (mitoribosome) processivity and undergo cell death following pharmacological inhibition of mitochondrial translation, regardless of their multidrug/mitochondrial resistance and stem-like phenotypes. Mechanistically, inhibiting mitoribosomes induced the mitochondrial stress-activated integrated stress response (ISR), leading to downregulation of c-MYC/N-MYC proteins prior to neuroblastoma cell death, which could be both rescued by the ISR inhibitor ISRIB. The ISR blocks global protein synthesis and shifted the c-MYC/N-MYC turnover toward proteasomal degradation. Comparing models of various neuroectodermal tumors and normal fibroblasts revealed overexpression of MYC proteins phosphorylated at the degradation-promoting site T58 as a factor that predetermines vulnerability of MYC-driven neuroblastoma to mitoribosome inhibition. Reducing N-MYC levels in a neuroblastoma model with tunable *MYCN* expression mitigated cell death induction upon inhibition of mitochondrial translation and functionally validated the propensity of neuroblastoma cells for MYC-dependent cell death in response to the mitochondrial ISR. Notably, neuroblastoma cells failed to develop significant resistance to the mitoribosomal inhibitor doxycycline over a long-term repeated (pulsed) selection. Collectively, we identify mitochondrial translation machinery as a novel synthetic lethality target for multidrug-resistant MYC-driven tumors.

## Introduction

Acquisition of aggressive dedifferentiated phenotype and therapy-induced multidrug resistance is the major cause of cancer therapy failure. Despite efforts, therapies that would overcome resistance mechanisms to kill all cancer cells including tumor-repopulating cancer stem-like cells remain elusive. Mitochondria have recently emerged as therapeutic targets in refractory cancers [1]. Besides serving as metabolic hubs, mitochondria integrate crucial roles in stemness maintenance [1], drug resistance [2], and cell death regulation [3]. Inhibiting mitochondrial processes has shown promising results in the most common tumor types, sensitizing resistant cancer cells to conventional chemotherapeutics [1]. However, our understanding of mitochondrial vulnerabilities in pediatric malignancies is limited.

Neuroblastoma is the most common extracranial childhood tumor. High-risk neuroblastomas are frequently driven by either N-MYC or c-MYC upregulation and have an extremely poor prognosis with 5-year overall survival of ~50% [4, 5]. Among other functions, MYC oncogenic transcription factors induce expression of genes involved in mitochondrial biogenesis [6–9] and mitochondria-dependent metabolism [10–12]. Importantly, we have recently demonstrated that mitochondria from therapy-resistant tumor cells often show attenuated apoptotic signaling, which largely contributes to neuroblastoma multidrug resistance [13]. Here, we therefore investigated potential mitochondrial dependencies, testing mitochondria as direct targets to overcome multidrug resistance in neuroblastoma. For this purpose, we took advantage of diverse mitochondrial inhibitors repurposed to target drug-resistant and/or stem-like cells in other cancers [14–18].

While mitochondria carry their own genome (mtDNA) and distinct transcription and translation machinery, their function heavily relies on nuclear-encoded mitochondrial proteins. Hence, mitochondrial perturbations must be efficiently relayed to cytosol and nucleus to orchestrate mitochondrial homeostasis, including proper stoichiometry of mitochondrial proteins [19]. Mitochondrial components of this retrograde signaling were only recently discovered, with inner mitochondrial membrane metalloprotease OMA1 identified as the major upstream regulator [20, 21]. We now provide insights into how this conserved mitochondrial stress-induced signaling might be exploited in neuroblastoma treatment. We demonstrate that disruption of mitochondrial proteostasis by mitoribosome inhibitors activates integrated stress response (ISR), in part via OMA1, which leads to c-MYC/N-MYC downregulation and cell death preferentially in neuroblastoma cells that rely on elevated MYC proteins. Our results reveal a novel mechanism of synthetic lethality that offers a promising therapeutic strategy to treat otherwise refractory MYC-driven tumors.

## Results

### Mitochondria-targeting inhibitors overcome emergent multidrug resistance in post-therapy neuroblastoma cells

**Fig. 1:**
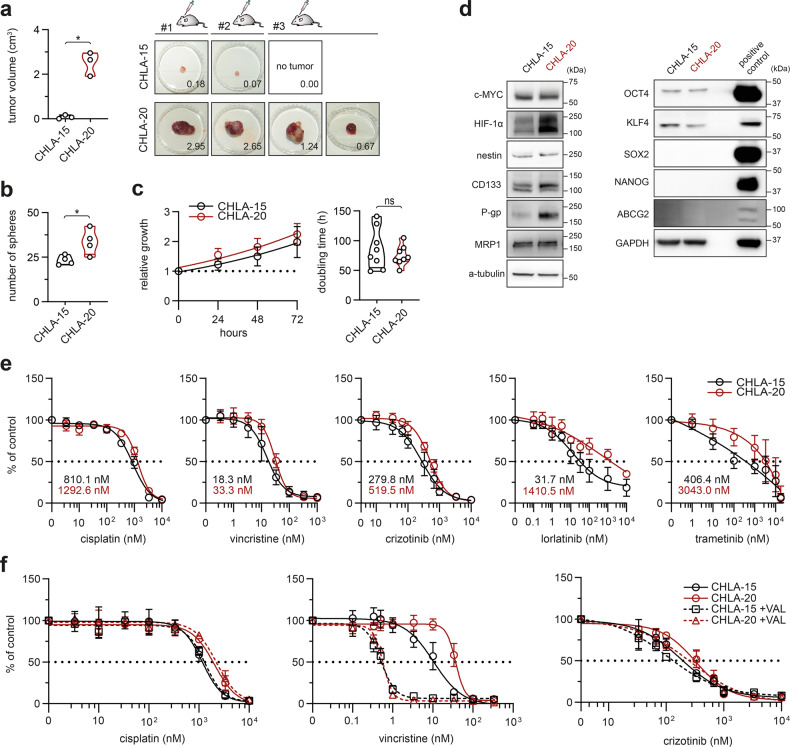
Therapy-naive CHLA-15 and post-therapy CHLA-20 near-isogenic cell lines provide a useful model of therapy-induced drug resistance and cancer stemness in neuroblastoma. **a** Mean volume of tumors per mice (left panel) and individual xenograft tumors (right panel) formed by CHLA-15 and CHLA-20 cells respectively after 29 days of injection in NSG mice. Right panel, numbers indicate the volume (cm^3^) of individual tumors. Also note the difference in the absolute tumor-forming efficiency (CHLA-15, 2/3 mice vs. CHLA-20, 3/3 mice). Left panel, data are presented as mean ± SD, biological *n* = 3 mice per group. **b** CHLA-20 are endowed with increased neurosphere formation capacity compared with CHLA-15, biological *n* = 4, technical *n* = 3. **c** MTT cell viability assay analysis showed no significant difference in growth rate (left panel; data are mean ± SD) or calculated doubling times (right panel) of the cell line pair, biological *n* = 8, technical *n* ≥ 4. **d** Western blot analysis of stemness transcription factors and CSC-related markers. Blots are representative of at least three experiments. Densitometric analysis is provided in Fig. S1. **e** Sensitivity to diverse chemotherapeutics tested by MTT assay after 72 h of treatment (calculated IC_50_ values are indicated). Data are presented as mean ± SD, biological *n* = 4, technical *n* = 3. **f** In vitro viability curves after 72 h exposure to drugs alone or with 0.5 μM of P-gp inhibitor valspodar (VAL). MTT data presented as mean ± SD, biological *n* = 3, technical *n* = 3. Statistical significance was determined by unpaired two-tailed Student’s t-test (**a**–**c**), *p < 0.05, ns not significant.

To establish a suitable model for our study, we first characterized a pair of *MYC*-amplified therapy-naive CHLA-15 and drug-resistant CHLA-20 neuroblastoma cell lines derived from tumors of the same patient at diagnosis and at relapse after multimodal therapy, respectively [22]. Functionally, these cell lines differed in their stem cell-like characteristics, with CHLA-20 cells showing a markedly enhanced capacity to initiate tumors in NSG mice (Fig. 1a) and form neurospheres in vitro (Fig. 1b) while maintaining a similar growth rate (Fig. 1c). Pointing to a complex fine-tuning of stem-like traits [23, 24], both cell lines expressed similar levels of the c-MYC oncoprotein and other common stemness-associated markers, except for the upregulation of HIF-1α and OCT4 in CHLA-20 (Figs. 1d, S1a).

Previously, CHLA-20 cells were reported more resistant to diverse chemotherapeutics than CHLA-15 [13, 22]. Validating this phenotype in our experimental settings, CHLA-20 cells exhibited broad resistance (1.6-fold to 44.5-fold difference vs. CHLA-15) to both DNA and non-DNA targeting drugs. The latter even included modern targeted agents never used to treat the donor patient’s tumor, i.e., crizotinib and lorlatinib, inhibiting ALK kinase (both CHLA-15 and CHLA-20 harbor the same *ALK*R1275Q activating mutation), or the MEK inhibitor trametinib (Fig. 1e). Of the common multidrug efflux pumps examined, only P-glycoprotein (P-gp) was upregulated in CHLA-20 compared with the therapy-naive CHLA-15 (Figs. 1d, S1b). However, P-gp upregulation was unlikely the mechanism underlying the broad resistance of CHLA-20 cells. First, it cannot explain resistance to cisplatin that is not a P-gp substrate. Second, when we inhibited P-gp by valspodar, CHLA-20 still retained enhanced resistance to drugs that are known P-gp substrates, such as crizotinib [25] (Fig. 1f). This is in line with our previous findings demonstrating that CHLA-20 has diminished sensitivity to apoptosis induction directly at the level of isolated mitochondria [13]. Together, the CHLA-15/CHLA-20 pair represents a useful model of emergent therapy resistance and cancer stemness in neuroblastoma.

**Fig. 2:**
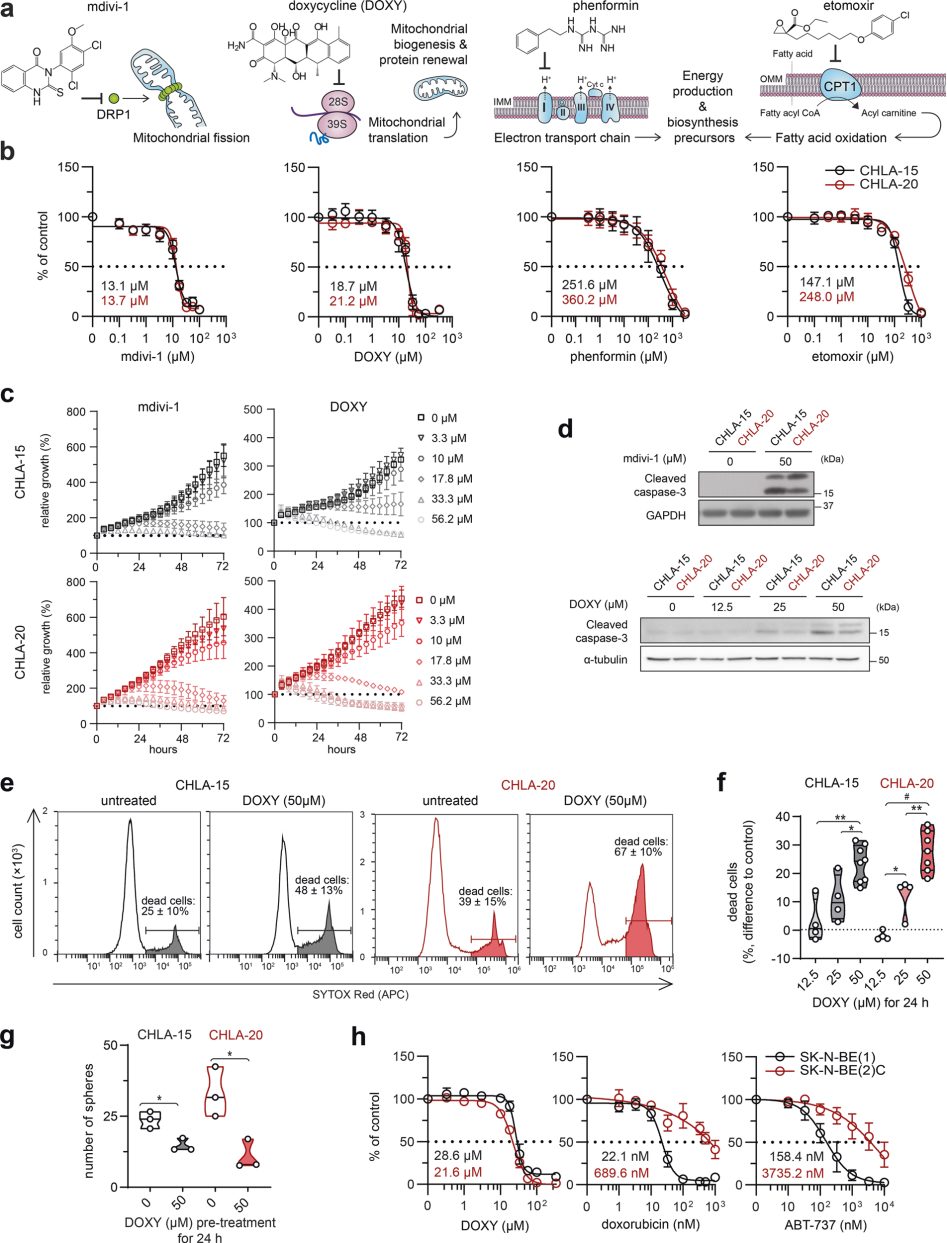
Therapy-naive and drug-resistant neuroblastoma cells retain sensitivity to inhibitors of mitochondrial quality control. **a** An overview of utilized mitochondrial inhibitors with indicated mechanism of action. **b** MTT cell viability assay analysis after 72-h treatment showed no significant difference in sensitivity to mitochondrial inhibitors between therapy-naive CHLA-15 and post-therapy CHLA-20 cell lines (calculated IC_50_ values are indicated). Data are presented as mean ± SD, biological *n* ≥ 3, technical *n* = 3. **c** Live-cell imaging growth rate analysis of CHLA-15 and CHLA-20 treated with indicated concentrations of mdivi-1 and DOXY. Data are presented as mean ± SD, biological *n* ≥ 3, technical *n* = 3. Supporting Supplementary Videos 1, 2 are provided. **d** Western blotting of the cleaved caspase-3 showed that DOXY and mdivi-1 treatment for 24 h induced apoptosis in both CHLA-15 and CHLA-20. Blots are representative of three experiments. **e**, **f** Flow cytometry analysis of cell viability after DOXY treatment for 24 h. Representative histograms including percentages of SYTOX Red-positive dead cells as mean ± SD, biological *n* ≥ 7 (**e**) and the difference in percentages of SYTOX Red-positive dead cells after indicated treatment vs. untreated controls (**f**). **g** Pretreating neuroblastoma cells with 50 µM DOXY for 24 h reduced their neurosphere formation capacity. Notably, the inhibition of sphere-formation capacity was more pronounced in CHLA-20 cells which exhibit enhanced stem-like traits (3-fold reduction relative to untreated control) compared with CHLA-15 (1.6-fold reduction); biological *n* = 3, technical *n* = 3. **h** CellTiter-Glo cell viability assay analysis of therapy-naive SK-N-BE(1) and post-therapy SK-N-BE(2)C after 72-h treatment (calculated IC_50_ are indicated) showed their similar sensitivity to DOXY whereas post-therapy SK-N-BE(2)C were found resistant to conventional chemotherapy drugs (see also Fig. S2) or a BH3 mimetic, ABT-737, inhibiting multiple anti-apoptotic BCL-2 proteins. Data are presented as mean ± SD, biological *n* = 3, technical *n* = 3. Statistical significance was determined by one-way ANOVA followed by Tukey’s multiple comparisons test (**f**) and by unpaired two-tailed Student’s t-test (**g**), *p < 0.05, **p < 0.01, #*p* < 0.0001.

To investigate potential mitochondrial vulnerabilities in this model, we treated both cell lines with several mitochondria-targeting drugs (Fig. 2a, b). Mitochondrial ATP production inhibitors, phenformin (inhibits complex I in the electron transport chain [1, 15]) and etomoxir (inhibits carnitine-palmitoyl transferase-1 [1, 14]), showed inefficient and reduced cell viability only at concentrations multiple times exceeding their selective or clinically tolerable profiles [26–29], with CHLA-20 still retaining a slightly increased resistance to these drugs (1.4-fold to 1.7-fold vs. CHLA-15; Fig. 2b). In contrast, inhibition of dynamin-related protein 1 (DRP1) by mdivi-1 and blocking mitochondrial protein synthesis by doxycycline (DOXY) substantially suppressed cell growth and induced apoptosis with a similar efficiency in both cell lines (Fig. 2b–d, Supplementary Videos 1, 2). Importantly, the effective DOXY concentrations (IC_50_ ~20 µM for 72 h) were within the range that shows a great clinical safety profile even during prolonged treatment [30, 31] and in young children [32, 33].

Both mitochondrial translation and DRP1-mediated mitochondrial fission are crucial for mitochondrial renewal [34], suggesting that survival of neuroblastoma cells was dependent on efficient mitochondrial quality control irrespective of their drug-resistant state. We decided to explore this dependency by focusing on mitochondrial translation inhibition, using DOXY as one of the FDA-approved ribosome-targeting antibiotics that might be readily repurposed for potential anticancer therapies. Flow cytometric analysis confirmed the dose-dependent effects of DOXY leading to the induction of cell death in the CHLA-15/CHLA-20 pair (Fig. 2e, f). Viable DOXY-pretreated cells also formed significantly fewer spheres (Fig. 2g) which is a proxy for the capacity of DOXY to eliminate stem-like tumor-initiating neuroblastoma cells. We next validated the therapeutic potential of mitochondrial translation inhibition in another model pair of *MYCN*-amplified therapy-naive SK-N-BE(1) and drug-resistant SK-N-BE(2)C neuroblastoma cells, the latter being even more sensitive to DOXY (Figs. 2h, S2). Collectively, these results revealed that targeting mitochondrial translation is efficient against bulk as well as drug-resistant/stem-like neuroblastoma cells.

### Inhibiting mitochondrial translation downregulates major oncoproteins and reveals a vulnerability shared across multiple neuroblastoma cell lines

**Fig. 3:**
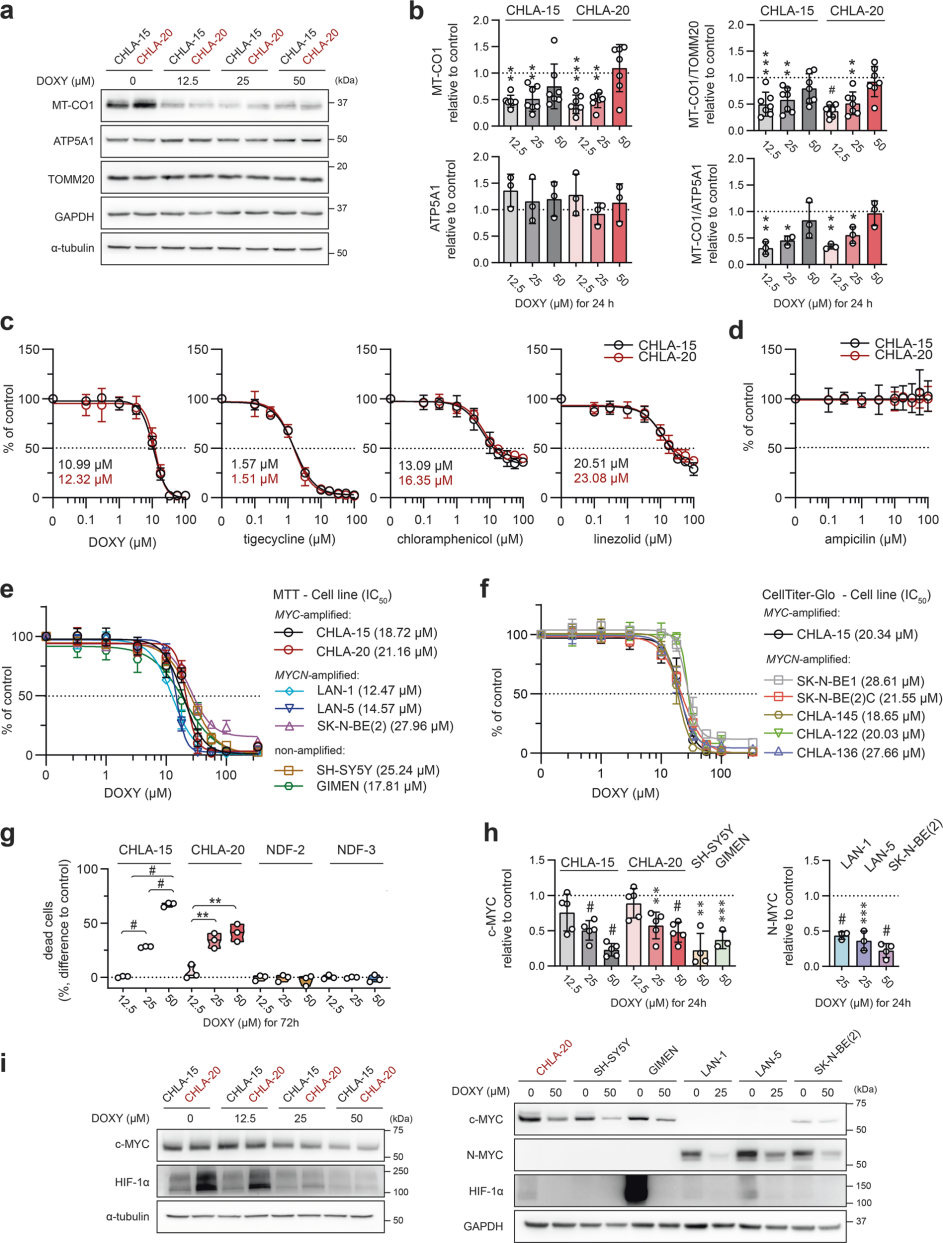
DOXY-mediated inhibition of mitochondrial protein synthesis impairs cell viability and reduces oncogenic transcription factors across a panel of neuroblastoma cells. **a**, **b** Expression of mitochondrial proteins, mitochondrial-encoded MT-CO1 and nuclear-encoded proteins ATP5A1 and TOMM20, after 24-h DOXY treatment analyzed by western blotting (**a**) and subsequent densitometry (**b**). Normalized protein levels are plotted relative to untreated controls, mean ± SD. **c**, **d** MTT cell viability assay analysis of 6-day treatment with DOXY and other FDA-approved antibiotics targeting procaryotic ribosomes, tigecycline, chloramphenicol, and linezolid (**c**), and targeting bacterial cell wall synthesis, ampicillin (**d**). Calculated IC_50_ are indicated. Data presented as mean ± SD, biological *n* = 4, technical *n* = 3. **e** MTT and **f** CellTiter-Glo cell viability assay analysis after 72-h treatment revealed all neuroblastoma cell lines to be highly sensitive to DOXY. Respective IC_50_ values are indicated in brackets. Data points are mean ± SD, biological *n* ≥ 3, technical *n* = 3. **g** Cell death rate of neuroblastoma cells (CHLA-15 and CHLA-20) and neonatal dermal fibroblasts (NDF-2 and NDF-3) was analyzed after 72 h of DOXY treatment by flow cytometry using SYTOX Red staining. Data are presented as the difference in percentages of SYTOX Red-positive dead cells after indicated treatment vs. respective untreated controls, biological *n* = 3. **h**, **i** Densitometric analysis (**h**) of western blotting detection (**i**) revealed downregulation of c-MYC, N-MYC, and HIF-1α across a panel of neuroblastoma cell lines treated with indicated concentrations of DOXY for 24 h. Normalized protein levels are plotted relative to untreated controls, mean ± SD. Densitometric analysis of HIF-1α is provided in Fig. S4. Statistical significance was determined by one-way ANOVA followed by Tukey’s multiple comparisons test (**b**, **g**, **h**), *p < 0.05, **p < 0.01, ***p < 0.001, #*p* < 0.0001.

Given the significant anticancer effects of DOXY, we further examined mechanisms underlying its activity. In line with previous studies [35–37], DOXY treatment induced an imbalance of mtDNA-encoded and nuclear-encoded mitochondrial proteins. While mtDNA-encoded cytochrome c oxidase I (MT-CO1) was downregulated in DOXY-treated cells, the levels of nuclear-encoded ATP synthase alpha-subunit 1 (ATP5A1) or mitochondrial import receptor subunit TOM20 homolog (TOMM20) were unaffected (Fig. 3a, b). Validating the general significance of our observations, we also detected similar growth inhibitory effects using other antibiotics that target bacterial, and thus mitochondrial ribosomes. Both tigecycline, a DOXY-related tetracycline derivate, and DOXY-unrelated antibiotics, linezolid and chloramphenicol, reduced cell viability in a dose-dependent manner (Fig. 3c). In contrast, ampicillin, a bacterial cell wall synthesis inhibitor not interfering with ribosome activity, did not affect the neuroblastoma cell growth (Fig. 3d). These results confirmed that the anti-neuroblastoma effects of DOXY were mediated by its specific binding to mitoribosomes leading to suppression of mitochondrial protein synthesis.

We next utilized a panel of twelve neuroblastoma cell lines, including MYC- or MYCN-amplified and non-amplified clones, to test whether mitochondrial translation might be a common vulnerability in high-risk neuroblastoma. Using two different viability assays, we found all examined cell lines highly sensitive to DOXY (Fig. 3e, f). On the contrary, when tested in nonmalignant neonatal dermal fibroblasts NDF-2 and NDF-3, DOXY limited proliferation only at much higher concentrations and did not deteriorate cell viability (Figs. 3g, S3, Supplementary Video 2), which suggests a potentially favorable therapeutic window of mitochondrial translation-targeted therapies in neuroblastoma.

Independent of genomic amplification, overexpression of N-MYC or c-MYC associates with the worst outcomes in neuroblastoma [38]. Yet, strategies targeting the MYC proteins for neuroblastoma treatment remain limited to preclinical studies [39]. Together with HIF-1α, another transcription factor associated with poor neuroblastoma prognosis, MYC proteins are known to affect mitochondrial biogenesis and metabolism [6–12]. Strikingly, DOXY-mediated inhibition of mitochondrial translation led to a dose-dependent downregulation of these transcription factors (Figs. 3h, i, S4), pointing to a novel therapeutically promising approach for inhibiting MYC proteins in multidrug-resistant high-risk neuroblastoma.

### DOXY disrupts mitochondrial morphology, suppresses mitochondrial fission machinery and primes mitochondria for apoptosis

**Fig. 4:**
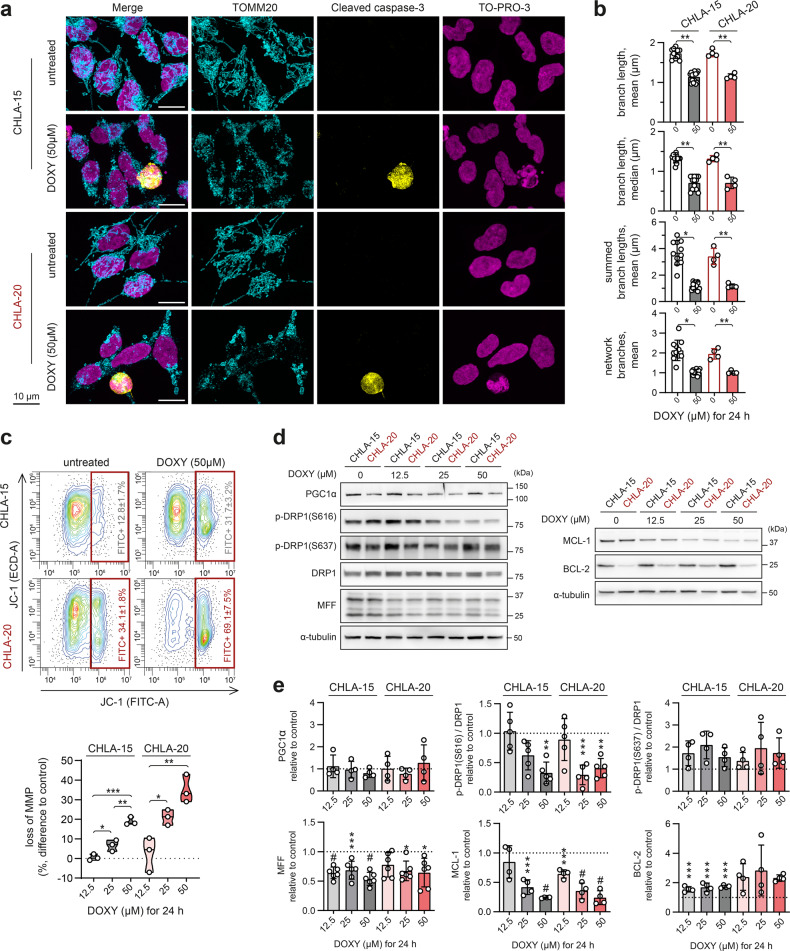
DOXY-mediated inhibition of mitochondrial translation disrupts mitochondrial morphology and fission machinery and primes mitochondria for apoptosis. **a** Mitochondrial morphology–visualized by immunofluorescence staining of TOMM20 (cyan)–revealed signs of fragmentation and swelling in most cells after 24-h treatment with 50 µM DOXY. At this timepoint, apoptosis marked by cleaved caspase-3 (yellow) and fragmented nuclei (TO-PRO-3; magenta) was detected only in a subset of cells. Maximum intensity projections of confocal microscopy Z-stacks are shown. **b** Image analysis by ImageJ plug-in tool MiNA - Mitochondrial Network Analysis confirmed disrupted mitochondrial morphology in cells treated with 50 µM DOXY for 24 h. Data are presented as parameters determined for individual field of vision images, mean ± SD. **c** Flow cytometry using JC-1 probe after 24-h DOXY treatment revealed dose-dependent loss of mitochondrial potential. Upper panel, representative contour plots; percentages of cells with depolarized mitochondria are presented as mean ± SD, biological *n* = 3. Bottom panel, the differences in percentages of cells with depolarized mitochondria after indicated treatment vs. untreated cells. **d**, **e** Western blotting detection (**d**) and densitometric analysis (**e**) of proteins related to mitochondrial dynamics and BCL-2 anti-apoptotic proteins after 24-h DOXY treatment in indicated concentrations. Normalized protein levels are plotted relative to untreated controls, mean ± SD. Statistical significance was determined by unpaired two-tailed Student’s t-test (**b**) and by one-way ANOVA followed by Tukey’s multiple comparisons test (**e**), *p < 0.05, **p < 0.01, ***p < 0.001, #*p* < 0.0001.

Microscopically, most DOXY-treated neuroblastoma cells showed substantially impaired mitochondrial morphology and disrupted mitochondrial network (Fig. 4a, b). However, cleaved caspase-3 was detected only in a fraction of these cells, which suggests that the collapse of mitochondrial network was an early event after DOXY-induced inhibition of mitochondrial translation, priming mitochondria to apoptosis (Fig. 4a). As demonstrated by JC-1 probe, DOXY treatment disrupted mitochondrial membrane potential in a dose-dependent manner (Fig. 4c), which corresponded with the cleaved caspase-3 levels detected in cells treated with increasing concentrations of DOXY (Fig. 2d).

The inhibition of mitochondrial translation did not affect the master regulator of mitochondrial biogenesis PGC1α (Fig. 4d, e). However, mitochondrial fission machinery, essential for the maintenance of mitochondrial health [40] and adjustment of mitochondrial functions [41], was suppressed after DOXY treatment. We found significant downregulation of fission-active DRP1 phosphorylated at Ser616, p-DRP1(S616), whereas its inactive form phosphorylated at Ser637, p-DRP1(S637), remained unaffected. Mitochondrial fission factor (MFF), a DRP1 adaptor protein, was also decreased upon DOXY treatment (Fig. 4d, e). At the level of anti-apoptotic BCL-2 family proteins, we identified a marked ~4-fold decrease of MCL-1 together with a ~2-fold BCL-2 upregulation (Fig. 4d, e). As CHLA-15 and CHLA-20 are both BCL-2-dependent [42, 43], these changes unlikely contributed to the DOXY-induced apoptosis.

### Mitochondrial stress-activated ISR is an early event during DOXY-mediated inhibition of mitochondrial translation

**Fig. 5:**
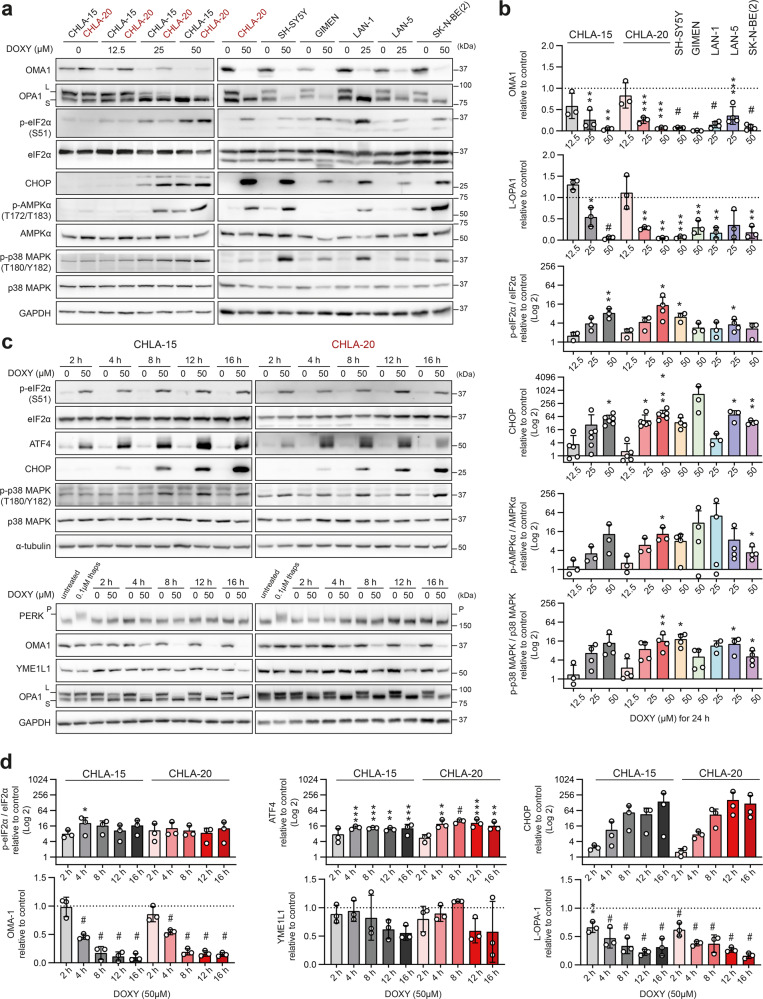
Inhibition of mitochondrial protein synthesis leads to early activation of OMA1-mediated ISR. **a**–**d** Western blotting detection and densitometric analysis of markers of mitochondrial stress, ISR and stress signaling kinases in a panel of neuroblastoma cells after DOXY treatment for 24 h (**a**, **b**) and over a 16-h time course (**c**, **d**). Normalized protein levels are plotted relative to untreated controls, mean ± SD. Treatment with 0.1 µM thapsigargin for 1 h served as a positive control of PERK phosphorylation, assessed by a reduced electrophoretic mobility in 6% polyacrylamide gel. Western blotting and densitometric analysis of YME1L1 after 24-h DOXY treatment and densitometric analysis of p-p38 MAPK (T180/Y182)/p38 MAPK during 16-h DOXY treatment are provided in Fig. S6b, c, respectively. Statistical significance was determined by one-way ANOVA followed by Tukey’s multiple comparisons test (**b**, **d**), *p < 0.05, **p < 0.01, ***p < 0.001, #*p* < 0.0001.

The identified mitochondrial protein imbalance, disrupted morphology and dynamics, and the loss of membrane potential collectively underpin a severe mitochondrial stress induced in DOXY-treated neuroblastoma cells. Recent studies showed that mitochondrial stress activates the OMA1-DELE1-HRI cascade, inducing the ISR by phosphorylation of its core mediator eIF2α [20, 21]. Due to the lack of reliable DELE1- or activated HRI kinase-specific antibodies, we assessed the activity of the key upstream and downstream regulators of this pathway. This analysis revealed consistent effects across a panel of neuroblastoma cells. DOXY-mediated inhibition of mitochondrial translation activated mitochondrial stress sensor OMA1, marked by its autocatalytic depletion and cleavage of its substrate optic atrophy-1 (OPA1) [44], and led to the induction of ISR, marked by upregulation of phosphorylated eIF2α, p-eIF2α(S51), and the key ISR effector, CHOP [45] (Figs. 5a, b, S5).

Long, intact L-OPA1 isoforms are crucial for the inner mitochondrial membrane fusion and their specific cleavage into short, fusion-inactive S-OPA1 forms by OMA1 induces the collapse of the mitochondrial network and promotes apoptosis [46, 47]. Consistently, the apparent degradation of L-OPA1 (Fig. 5a–d) likely explains the mitochondrial fragmentation observed in DOXY-treated cells, which counterintuitively showed impaired mitochondrial fission machinery (Fig. 4). Importantly, OMA1 activation (assessed by L-OPA1 processing) and increased levels of p-eIF2α(S51) and ATF4, a transcription factor promoting CHOP expression, were detected already after 2-h DOXY treatment (Fig. 5c, d), indicating the importance of mitochondrial ISR during the initial phase of mitochondrial translation inhibition by DOXY.

PERK is a canonical kinase that phosphorylates eIF2α in response to endoplasmic reticulum (ER) stress from unfolded proteins. To evaluate whether ER stress was involved in the DOXY-induced ISR, we treated cells with ER stressor thapsigargin. However, PERK activation was detected only in thapsigargin-treated cells and, conversely, reciprocal degradation of mitochondrial stress-activated proteases OMA1 and YME1L1 and cleavage of L-OPA1 [44, 48] was found only in DOXY-treated samples (Figs. 5c, d, S5, S6a, b). These results demonstrate that DOXY-mediated inhibition of mitochondrial translation leads to mitochondrial stress that directly activates ISR independently of PERK and ER stress signaling.

Prolonged mitochondrial dysfunction is associated with ATP depletion, known to induce AMPK activity [49], and with overproduction of reactive oxygen species (ROS), activating redox-sensitive signaling cascades including p38 MAPK [50]. Indeed, we detected activation of these pathways, marked by increased levels of p-AMPKα(T172/T173) and p-p38 MAPK(T180/Y182), at later time points and DOXY concentrations that resulted in almost fully processed OPA1 and markedly degraded OMA1 (Figs. 5a–d, S5, S6c). Thus, the induction of AMPK and p38 MAPK signaling is a subsequent event following the mitochondrial stress-activated ISR in an OMA1-dependent fashion. However, we demonstrated that these late effects of DOXY-mediated inhibition of mitochondrial translation further contribute to neuroblastoma apoptosis, as inhibiting p38 MAPK by its specific inhibitor SB203580 partially prevented DOXY-induced activation of caspase-3 (Fig. S6d).

### Mitochondrial ISR links inhibited mitochondrial translation with degradation of short-lived oncoproteins and induction of cell death

**Fig. 6:**
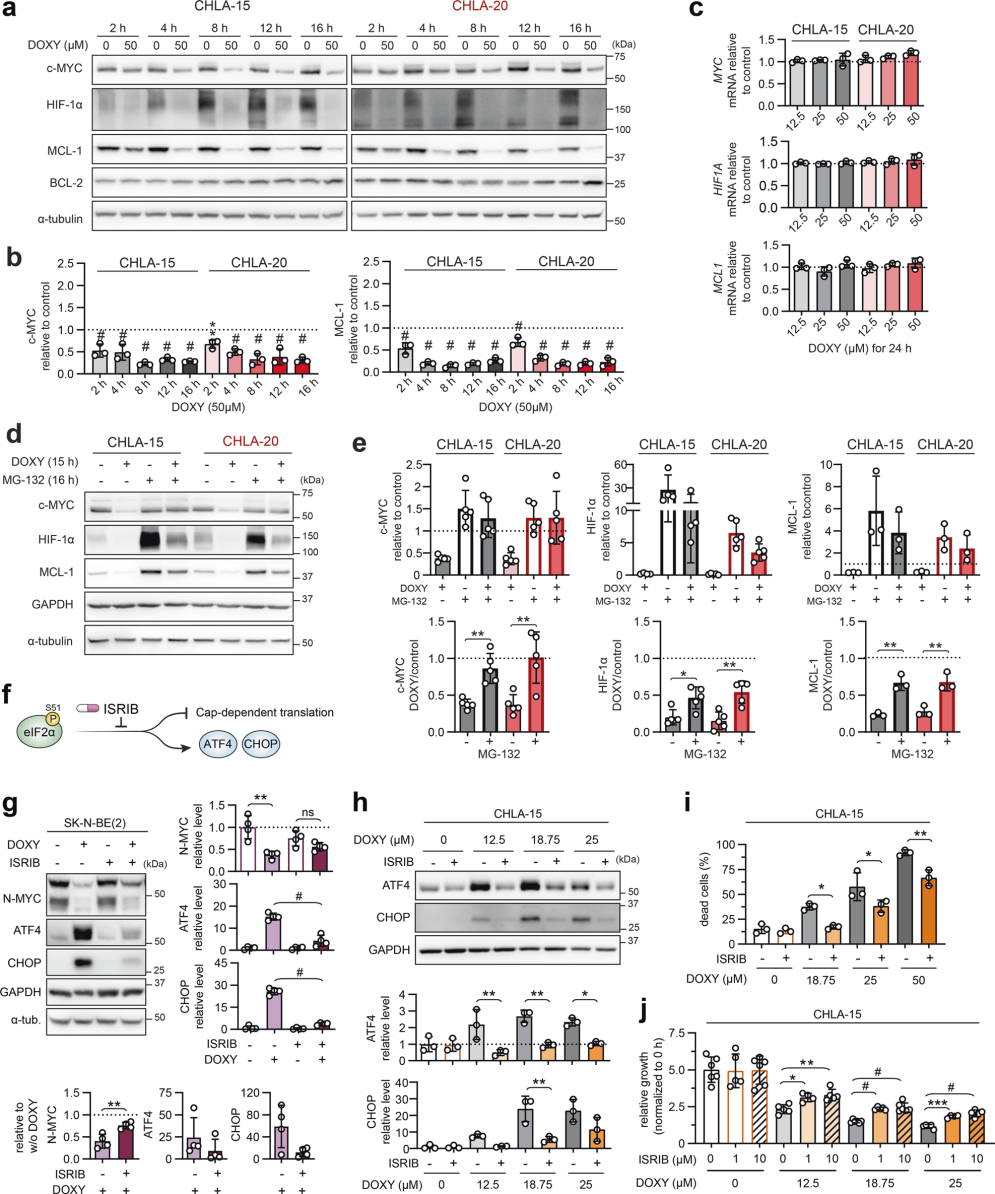
DOXY-induced ISR activation corresponds with early downregulation of short-lived oncoproteins and contributes to neuroblastoma growth inhibition and cell death. **a**, **b** Western blotting detection (**a**) of c-MYC, HIF-1α, and anti-apoptotic BCL-2 family proteins in CHLA-15 and CHLA-20 treated with 50 µM DOXY for 2–16 h followed by densitometric analysis (**b**) of c-MYC and MCL-1. Normalized protein levels are plotted relative to untreated controls, mean ± SD. Densitometric analysis of HIF-1α and BCL-2 is provided in Fig. S6c. **c** No change in the transcription of *MYC*, *HIF1A,* and *MCL1* was detected by RT-qPCR upon 24-h DOXY treatment. Normalized mRNA levels plotted relative to untreated controls, mean ± SD. **d**, **e** Western blotting detection (**d**) and densitometric analysis (**e**) of c-MYC, HIF-1α, and MCL-1 after DOXY treatment of neuroblastoma cells with inhibited proteasome. To block proteasome activity, CHLA-15 and CHLA-20 were pretreated with MG-132 (1 µM for MCL-1 analysis, 5 µM for c-MYC and HIF-1α) and after 1 h, 50 µM DOXY was added for additional 15 h. Upper panel, normalized protein levels are plotted relative to fully untreated controls (dashed line). Lower panel, ratios of normalized protein levels in DOXY-treated cells (w/o or with MG-132) and respective controls (w/o or with MG-132), mean ± SD. **f** ISRIB reverts p-eIF2α(S51)-mediated attenuation of cap-dependent translation [59], thus restores global protein synthesis while suppressing translation of mRNAs encoding ISR effectors such as ATF4 and CHOP. **g** Western blotting detection and densitometric analysis of N-MYC, ATF4, and CHOP in *MYCN*-amplified SK-N-BE(2) treated for 24 h with 50 µM DOXY in the presence or absence of 1 µM ISRIB. To efficiently block the induction of ISR, cells were pretreated with ISRIB for 4 h prior to addition of DOXY. Right panel, normalized protein levels plotted relative to the average of all samples, mean ± SD. Lower panel, ratios of normalized protein levels in DOXY-treated cells and their respective controls (w/o or with ISRIB), mean ± SD. **h** Western blotting detection and densitometric analysis of ATF4 and CHOP in CHLA-15 treated for 72 h with different concentrations of DOXY w/o or with 1 µM ISRIB. Normalized protein levels are plotted relative to the average of all samples, mean ± SD. **i** Cell death rate of CHLA-15 analyzed by flow cytometry using SYTOX Red staining after 72-h treatment with 18.75–50 µM DOXY in the presence or absence of 1µM ISRIB. Data shown as percentages of SYTOX Red-positive dead cells after the indicated treatments, biological *n* = 3. **j** Live-cell imaging growth rate analysis of CHLA-15 concomitantly treated with DOXY and ISRIB in indicated concentrations for 72 h. Data are normalized to 0 h and presented as mean ± SD, biological *n* ≥ 4, technical *n* = 3. Statistical significance was determined by one-way ANOVA followed by Tukey’s multiple comparisons test (**b**, **c**, **g**–**j**) and unpaired two-tailed Student’s t-test (**e**, **g**), *p < 0.05, **p < 0.01, ***p < 0.001, #*p* < 0.0001.

Phosphorylation of eIF2α attenuates cap-dependent translation in favor of ISR-specific mRNAs, allowing for efficient degradation of accumulated proteins and restoration of proteostasis [51]. We hypothesized that the ISR-mediated blockage of global protein synthesis might concurrently downregulate proteins with rapid turnover, as these are more readily targeted for degradation. Notably, c-MYC [52], N-MYC [53], HIF-1α [54], and MCL-1 [55], all significantly downregulated by DOXY treatment (Fig. 3h, i), are known to undergo rapid proteasome-dependent degradation with half-lives of <1 h. Indeed, the early mitochondrial stress-activated ISR was accompanied by the c-MYC, HIF-1α, and MCL-1 downregulation that was detectable already upon 2-h treatment and progressed with time (Figs. 6a, b, S6c). In contrast, levels of BCL-2 that has a substantially longer half-life of ~20 h [56] were not significantly affected even after 16 h of DOXY treatment. Preferential downregulation of short-lived proteins upon the DOXY-induced ISR was further substantiated by the constant levels of the protein loading control GAPDH, which is a substrate for proteasome-independent chaperone-mediated autophagy with a half-life of ~40 h [57] (Fig. 6a, b). In line with this reasoning, activation of the ISR by thapsigargin was also sufficient to downregulate c-MYC, HIF-1α, and MCL-1 in neuroblastoma cells already within 1 h of treatment, whereas BCL-2 levels remained unchanged (Fig. S6a).

We next confirmed that DOXY treatment did not affect expression of c-MYC, HIF-1α, and MCL-1 at transcriptional levels (Fig. 6c). In contrast, proteasome inhibition by MG-132 almost completely reverted the DOXY-induced downregulation of c-MYC (Fig. 6d, e), including its phosphorylated form p-c-MYC(T58) (Fig. S7) prone to proteasomal degradation [58], and significantly rescued expression of HIF-1α and MCL-1 (Fig. 6d, e). Importantly, pretreating cells with the ISR inhibitor ISRIB, which reverses the effects of phosphorylated eIF2α and restores cap-dependent translation [59] (Fig. 6f), partially rescued the N-MYC downregulation in DOXY-treated cells (Fig. 6g). Thus, the attenuated cap-dependent protein synthesis and enhanced proteasomal degradation upon mitochondrial stress-activated ISR are the major mechanisms causing the downregulation of short-lived oncoproteins, including MYC proteins in neuroblastoma cells.

Following this finding, we asked whether inhibiting the DOXY-induced mitochondrial ISR also rescues the viability of neuroblastoma cells. Indeed, combining DOXY treatment with ISRIB suppressed expression of the ISR effectors ATF4 and CHOP (Fig. 6h), and reduced the cell death induction (Fig. 6i) and cell growth inhibition (Fig. 6j) when compared with cells treated with DOXY alone. Together, these results demonstrate mitochondrial ISR as the key pathway responsible for both downregulation of MYC proteins and induction of neuroblastoma cell death upon the mitoribosome inhibition.

### Rapid turnover of MYC proteins associates with sensitivity of MYC-driven neuroblastoma to DOXY-induced cell death

**Fig. 7:**
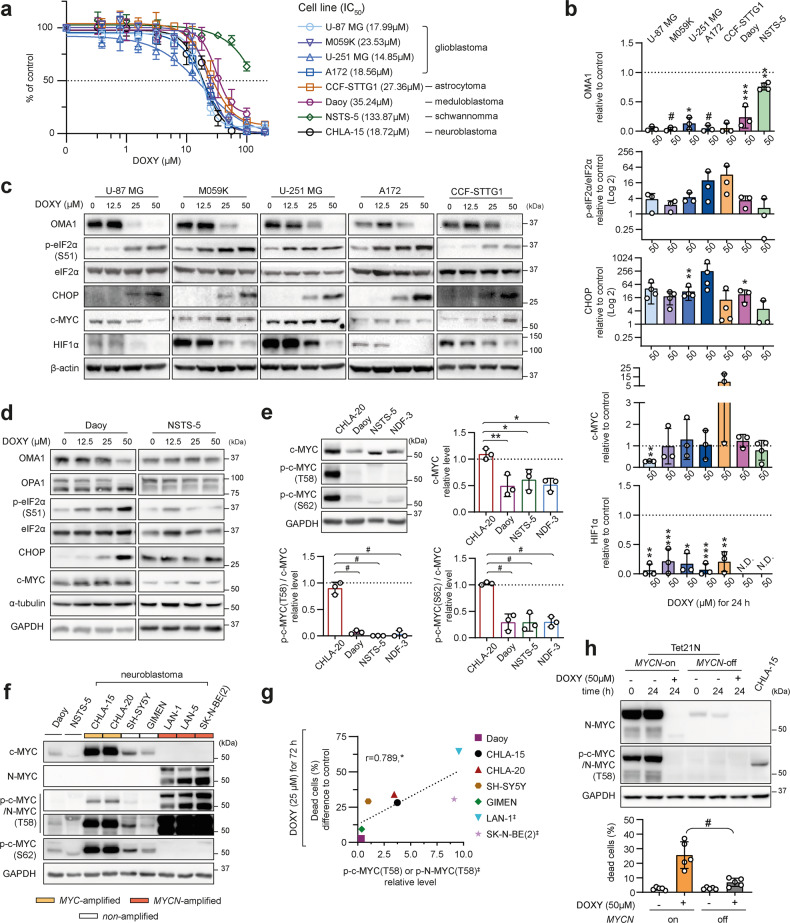
Overexpression and phosphorylation of MYC proteins correlate with sensitivity to cell death via mitochondrial ISR, conserved across various models of nervous system tumors. **a** MTT cell viability assay analysis was used to determine sensitivity of cell lines derived from various nervous system tumors to DOXY. Respective IC_50_ values for 72-h treatment are indicated in brackets. Data presented as mean ± SD, biological *n* ≥ 3, technical *n* = 3. **b**–**d** Densitometric analysis (**b**) of immunoblots (**c**, **d**) from lysates after 24-h DOXY treatment showed dose-dependent induction of mitochondrial stress and ISR in a panel of cell lines derived from different nervous system tumors. Contrary to neuroblastoma, this did not lead to consistent downregulation of c-MYC, although HIF-1α was significantly downregulated in nervous system tumors. Normalized protein levels are plotted relative to untreated controls, mean ± SD; N.D. – not detectable. Complete densitometric analysis is provided in Fig. S8. **e** Western blotting detection and densitometric analysis of c-MYC, p-c-MYC(T58), and p-c-MYC(S62) revealed that CHLA-20 neuroblastoma cells have significantly higher c-MYC level and extensively enhanced phosphorylation of c-MYC compared with Daoy, NSTS-5 and NDF-3 cells that were less sensitive to DOXY. Normalized protein levels are plotted relative to untreated control, mean ± SD. **f** Western blotting detection of c-MYC, p-CMYC/N-MYC(T58) and p-c-MYC(S62) in a panel of nervous system tumors. **g** A significant positive correlation between the percentage of dead cells after 72-h treatment with 25 μM DOXY and the basal levels of p-CMYC/N-MYC(T58) in untreated neuroblastoma models and Daoy medulloblastoma cells, r = Pearson correlation coefficient; ǂ, *MYCN*-amplified. Related flow cytometry and protein densitometry data are provided in Fig. S10. **h** Western blotting detection of N-MYC and p-c-MYC/N-MYC(T58) (upper panel; densitometric analysis is provided in Fig. S12a) and cell death rate flow cytometric analysis (lower panel) revealed that Tet21N *MYCN*-off cells with extensively downregulated N-MYC and p-c-MYC/N-MYC(T58) levels are significantly less sensitive to DOXY-induced cell death compared with N-MYC overexpressing Tet21N *MYCN*-on cells. Cell death rate was analyzed using SYTOX Red staining after 24-h treatment with 50 µM DOXY. Data are presented as percentages of SYTOX Red-positive dead cells after indicated treatment, biological *n* = 5. Statistical significance was determined by one-way ANOVA followed by Tukey’s multiple comparisons test (**b**, **e**, **h**) and by Pearson correlation (**g**), *p < 0.05, **p < 0.01, ***p < 0.001, #*p* < 0.0001.

To explore if the effects of DOXY-mediated mitochondrial translation inhibition are neuroblastoma specific, we introduced seven cell lines derived from different nervous system tumors. Consistent with our results in neuroblastoma, inhibiting mitochondrial translation by DOXY limited cell proliferation across the tested cell lines, except for NSTS-5 schwannoma cells (Fig. 7a). Similarly, induction of mitochondrial stress, activation of ISR and decrease of HIF-1α were detected in DOXY-responsive glioblastoma, astrocytoma, and medulloblastoma cells (Figs. 7b–d, S8). Intriguingly, markers of mitochondrial stress and ISR activation were also found in neonatal dermal fibroblasts (Fig. S9) that partially reduced proliferation but retained their viability in response to DOXY treatment (Figs. 3g, S3). This may suggest that the ISR is a conserved retrograde pathway that relays DOXY-induced imbalance in mitochondrial proteins but leads to cell type-dependent outcomes.

In contrast to neuroblastoma, we did not observe consistent downregulation of c-MYC in cell lines derived from other nervous system tumors (Figs. 7b–d, S8) or neonatal dermal fibroblasts (Fig. S9). Compared with other tumor types, *MYC*-amplified CHLA-20 neuroblastoma cells express markedly higher levels of c-MYC together with its phosphorylated forms, p-c-MYC(T58) and p-c-MYC(S62) (Fig. 7e). Phosphorylation at threonine 58 (T58), subsequent to serine 62 (S62) phosphorylation, is known to promote proteasomal degradation and rapid turnover of both c-MYC [58] and N-MYC [60].

Strikingly, we found cell types with relatively low levels of c-MYC and its phosphorylated forms to be substantially resistant to DOXY-induced cell death, as demonstrated in Daoy (Fig. S10), NSTS-5 (Fig. 7a) and NDF-3 cells (Figs. 3g, S3). Immunoblots using the antibody recognizing both p-c-MYC(T58) and p-N-MYC(T58) showed that these phosphorylated forms are markedly upregulated in MYC-driven neuroblastoma and their levels correlated with sensitivity to DOXY-induced cell death in a panel of neuroblastoma and medulloblastoma models (Figs. 7f, g, S11a, c, d). To functionally validate this MYC-dependent sensitivity, we utilized Tet21N neuroblastoma cells [61] with tunable N-MYC expression (for details refer to Methods section). Tet21N cells that expressed high levels of N-MYC and its T58-phosphorylated form (Tet21N *MYCN*-on) were significantly more sensitive to DOXY-induced cell death compared to their counterparts (Tet21N *MYCN*-off) where N-MYC expression was switched off (Figs. 7h, S12a). Of note, both *MYCN*-on and *MYCN*-off untreated cells showed very similar growth rates (Fig. S12b), which supports the conclusion that the outcomes of mitoribosome inhibition are MYC-dependent. Our data indicate that the high c-MYC/N-MYC levels and extensive T58 phosphorylation, priming MYC proteins for rapid degradation upon the mitochondrial ISR (Fig. S7), determine the propensity of MYC-driven neuroblastoma cells to cell death in response to inhibition of mitochondrial translation. Conversely, cell types lacking aberrantly upregulated expression and phosphorylation of MYC proteins, such as normal fibroblasts (Figs. 3g, 7e), did not undergo cell death after mitochondrial ISR activation, which suggests a promising opportunity for developing mitoribosome targeting therapies that would be efficient against MYC-driven neuroblastomas while sparing healthy tissues.

### Long-term repeated inhibition of mitochondrial translation does not induce highly resistant neuroblastoma phenotype

**Fig. 8:**
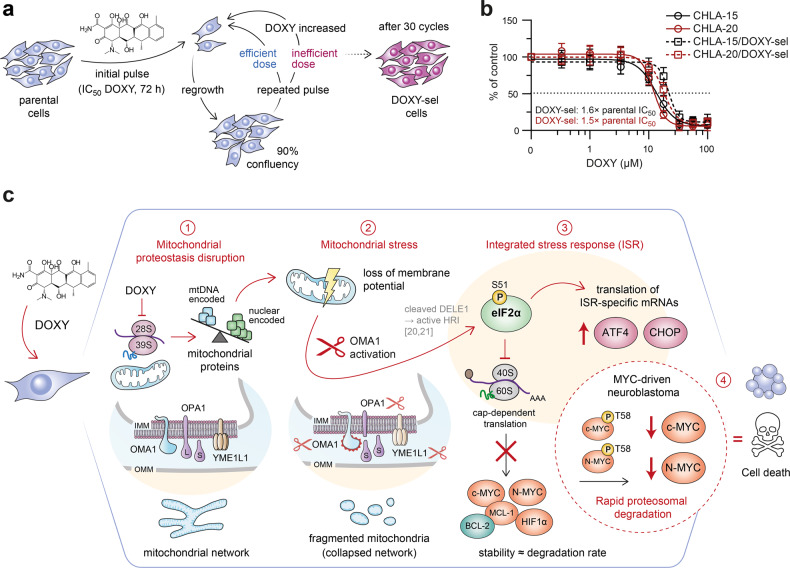
Inhibiting mitochondrial translation does not induce clinically relevant resistance and offers a promising therapeutic strategy for MYC-driven neuroblastoma. **a** A schematic overview of the pulsed-selection strategy mimicking the chemotherapy cycles in the clinic. Parental cells CHLA-15 and CHLA-20 were repeatedly exposed to gradually increasing concentrations of DOXY, starting from IC_50_ (determined for each cell line by MTT assay upon 72-h treatment) as follows: 3 cycles of IC_50_, followed by 3 cycles of 1.5-fold IC_50_ and finally 2-fold IC_50_ that remained highly efficient for additional 24 cycles. In each cycle, cells were treated for 72 h and let to regrow to approx. 90% confluency in fresh drug-free culture media before another round of DOXY treatment. **b** The sensitivity of parental cells and established DOXY-selected cells (CHLA-15/DOXY-sel and CHLA-20/DOXY-sel) to DOXY was compared by MTT cell viability assay after 72 h of treatment. Data are presented as mean ± SD, biological *n* = 3, technical *n* = 3. **c** A model summarizing synthetic lethal effects induced by inhibition of mitochondrial translation in MYC-driven neuroblastoma. Blocking mitochondrial ribosomes by DOXY disrupts mitochondrial proteostasis (1) which impairs mitochondrial function and leads to the mitochondrial stress-induced activation of OMA1 (2). Activated OMA1 mediates OPA1 cleavage, eventually resulting in collapsed mitochondrial network, and relays mitochondrial stress directly via the mitochondrial ISR without involving ER stress signaling (3). Besides preferential translation of ISR-specific mRNAs, attenuated cap-dependent translation leads to downregulation of short-lived proteins primed for rapid degradation, including p-c-MYC(T58) and p-N-MYC(T58), which sensitizes MYC-driven neuroblastoma to cell death induced by the inhibition of mitochondrial translation (4).

Target-specific resistance often develops between chemotherapy cycles. We therefore repeatedly exposed CHLA-15/CHLA-20 cells to DOXY to mimic multiple rounds of therapy and gradually select for the DOXY-resistant phenotype (DOXY-sel; Fig. 8a). However, even after 11 months and 30 cycles of selection, the cells remained highly sensitive to 2-fold of the initial IC_50_ of DOXY and the finally established DOXY-sel cell lines showed only ~1.5-fold resistance compared with parental cells (Fig. 8b). Both DOXY-sel cell lines exhibited downregulation of several proteins associated with poor neuroblastoma prognosis, including HIF-1α [62], P-gp, or MCL-1 (Fig. S13a, b). Given the marked P-gp downregulation in DOXY-sel cells, we next asked whether P-gp modulates sensitivity to DOXY. However, pharmacological inhibition of P-gp did not change the viability of parental cells treated with DOXY (Fig. S13c). Additional cell line-specific changes involved proteins targeted by DOXY treatment, including MT-CO1, eIF2α, or OMA1 (Fig. S13a, b). How these potentially compensatory events affect mitochondrial functions and the overall phenotype of DOXY-sel neuroblastoma cells will require further investigation.

Collectively, our data establish that perturbing mitochondrial function by DOXY-mediated targeting of mitochondrial protein synthesis triggers the mitochondrial ISR which in the context of MYC-driven neuroblastoma efficiently overcomes existing multidrug resistance mechanisms and eradicates aggressive tumor cells without inducing clinically relevant resistance (Fig. 8c). To confirm this concept of mitoribosomal synthetic lethality, we also replicated the key experiments with a tetracycline-unrelated mitoribosome inhibitor, chloramphenicol. This antibiotic was selected as it is known to bind to the large ribosomal subunit (DOXY binds to the small subunit of the mitoribosome), and as it was already found efficient against therapy-naive and drug-resistant neuroblastoma cells in this study (Fig. 3c). In line with the effects observed after DOXY treatment, chloramphenicol selectively inhibited mitochondrial translation (Fig. S14a) and induced mitochondrial ISR leading to the downregulation of c-MYC in neuroblastoma cells (Fig. S14b, c). Consistently, sensitivity of Tet21N cells to chloramphenicol-mediated inhibition of proliferation was also diminished by N-MYC downregulation (Fig. S14d). In contrast to DOXY, chloramphenicol did not induce OMA1 activity but degradation of other mitochondrial stress-activated protease YME1L1 was detected (Fig. S14b, c). This suggests that an alternative pathway distinct from the OMA1-DELE1-HRI cascade might relay the mitochondrial stress signals to eIF2α upon blocking the large mitoribosomal subunit. Nevertheless, the key effects observed after chloramphenicol treatment, i.e., the mitochondrial ISR activation leading to impaired neuroblastoma cell viability in a MYC-dependent manner, provide further evidence for the newly identified mitoribosomal synthetic lethality, highlighting the potential of repurposing ribosome-targeting antibiotics in neuroblastoma therapy.

## Discussion

Targeting mitochondria is an emerging strategy to overcome cancer drug resistance. Yet, molecular determinants that would guide efficient mitochondrial therapies are poorly understood, particularly in pediatric tumors. Here, we identified mitochondrial translation as a promising MYC-dependent target in multidrug-resistant neuroblastoma. Our results also implicate that mitochondrial quality control mechanisms, including mitochondrial dynamics and translation control, are essential for neuroblastoma cell survival irrespective of the drug resistance status, as demonstrated by inhibition of mitochondrial fission by mdivi-1 and disruption of mitoribosome processivity by DOXY (Fig. 2c–h, Supplementary Videos 1, 2).

Mechanistically, time course experiments showed that inhibition of mitoribosomes by DOXY induces the activity of mitochondrial metalloprotease OMA1 and initiates mitochondrial retrograde signaling via the central ISR mediator eIF2α. Short-running ISR normally orchestrates gene expression and protein translation to restore cellular homeostasis and maintain cell survival, in line with its reported pro-tumorigenic effects [63]. However, excessive or prolonged ISR is known to induce cell death in various cell types [51]. Interestingly, the ISR effector ATF4 was already shown to mediate cell death upon glutamine deprivation in *MYCN*-amplified neuroblastoma [64]. Consistently, we provide evidence that MYC-driven neuroblastoma cells are particularly vulnerable to the ISR-mediated cell death, while p38 MAPK appears to further favor apoptosis induced by the mitochondrial ISR. p38 MAPK is known to promote apoptosis [65–67] and likely synergizes with the ISR by directly phosphorylating CHOP [68], enhancing its pro-apoptotic activity [69].

Previously, DOXY-mediated inhibition of mitochondrial translation was shown to enhance ER-mitochondria connectivity [70] and was suggested to activate ISR via inducing ER stress in carcinomas [70, 71] and melanoma [18, 72]. On the contrary, we demonstrate that ER stress is not involved in DOXY-induced ISR in neuroblastoma, as eIF2α phosphorylation was not mediated by its upstream ER stress-activated kinase PERK. Our data are consistent with recent studies demonstrating mitochondrial stress sensor OMA1 as the major inducer of ISR via the OMA1-DELE1-HRI pathway [20, 21]. Similarly, we demonstrate that DOXY-induced mitochondrial protein imbalance disrupts mitochondrial morphology and membrane potential, inducing the ISR along the OMA1 activity detected already in early phases of the treatment. These results indicate that targeting the small mitoribosomal subunit by DOXY induces ISR via OMA1 signaling.

However, our data also revealed a potentially alternative, OMA1-independent mechanism of mitochondrial ISR upon blocking mitoribosomes, as targeting the large mitoribosomal subunit by chloramphenicol induced ISR without apparent OMA1 activation (Fig. S14). Of note, additional cell context-dependent signaling pathways cannot be excluded. Previously, knocking down components of the OMA1-DELE1-HRI pathway showed insufficient to completely block the DOXY-mediated ISR in embryonal kidney HEK293T cells [20]. However, in contrast to our models (Figs. 5, 7d, S5a, b), DOXY-treated HEK293T cells did not show any signs of L-OPA1 cleavage [20], suggesting cell type-dependent activation of OMA1 in response to the DOXY-mediated inhibition of mitochondrial translation. Similarly, DOXY structural analog tigecycline was recently shown to induce ISR via GCN2 signaling in colorectal adenocarcinoma and chronic myelogenous leukemia cells [73]. Together, these findings suggest that mitoribosomal inhibitors induce ISR via multiple signaling pathways and further investigation is needed to explore the context dependency.

A chronic ISR has been recently suggested to predict the sensitivity of melanoma cells to tigecycline-mediated inhibition of mitoribosomes [18]. In contrast, we did not observe any association between DOXY-induced cell death and the extent of ISR activation prior or after treatment. In fact, we found p-eIF2α(S51) and one of the major ISR effectors, CHOP, to be consistently upregulated by DOXY in nearly all tested cell lines (neuroblastoma: 11/11; other nerve tissue tumors: 6/7; normal fibroblasts: 1/1) regardless their vulnerability to DOXY-induced cell death. High c-MYC levels have been associated with the susceptibility of hematological malignancies to inhibition of mitochondrial protein synthesis [37, 74]. In this study, we found that the mitoribosome inhibition-induced ISR leads to degradation of short-lived MYC proteins and that the upregulation of their T58-phosphorylated forms, tagging MYC proteins for rapid proteasome degradation [58, 60, 75, 76], sensitizes MYC-driven neuroblastoma to inhibition of mitoribosomes.

Based on these findings, we propose mitochondrial translation as a novel synthetic lethal target that might be exploited to overcome multidrug resistance in MYC-driven neuroblastoma (Fig. 8c). We anticipate that further dissection of mitochondrial stress signaling pathways might enable identification of additional clinically relevant targets for the treatment of MYC-addicted tumors.

## Methods

### Cell culture and treatment

The following cell lines were used in the study: (i) neuroblastoma cell lines CHLA-15, CHLA-20, CHLA-122, CHLA-136, CHLA-145, SK-N-BE(1), SK-N-BE(2)C (obtained from the COG/ALSF Childhood Cancer Repository; www.cccells.org), GIMEN, LAN-1, LAN-5 (a kind gift of Prof. Lumír Krejčí), SH-SY5Y, SK-N-BE(2) (purchased from ECACC), and Tet21N (kindly provided by Dr. Frank Westermann); (ii) glioblastoma cell lines U-87 MG, M059K, U-251 MG, A-172 and astrocytoma cell line CCF-STTG1 (all purchased from ATCC); (iii) medulloblastoma cell line Daoy (purchased from ECACC); (iv) schwannoma cell line NSTS-5 (in-house derived from tumor tissue with written informed consent under IGA MZCR NR/9125-4 project approved by the Research Ethics Committee of the School of Medicine, Masaryk University, Brno, Czech Republic – approval no. 23/2005); (v) human neonatal dermal fibroblast cell lines NDF-2 and NDF-3 (#CC-2509; Lonza Bioscience, Durham, NC, USA; a kind gift of Dr. Tomáš Bárta). In addition, human pluripotent embryonal carcinoma cell line NTERA-2 (clone D1) purchased from ECACC (#01071221) served as a positive control shown in Fig. 1d. All cell lines were authenticated by STR profiling (Generi Biotech, Hradec Králové, Czech Republic; Westmead Institute of Medical Research, Westmead, NSW, Australia; Promega Geneprint 10, Madison, Wisconsin, USA) and routinely tested for mycoplasma by PCR [77].

Cell lines were cultured in a humidified atmosphere of 5% CO_2_ at 37 °C in media with supplements as detailed in Supplementary Tables 1, 2. Drug treatments were always performed the day after cell seeding. A detailed overview of the drugs used is provided in Supplementary Table 3. Tet21N cells express N-MYC from a *MYCN* Tet-off construct [61]. To induce *MYCN*-off state, Tet21N cells (*MYCN*-on) were pretreated with a non-cytotoxic and non-cytostatic dose of DOXY (2.25 μM) for 72 h, blocking *MYCN* expression under the control of Tet-off promoter. If not treated with 50 μM of DOXY, Tet21N *MYCN*-off cells were always maintained in media supplemented with 2.25 μM DOXY during subsequent experiments.

### Cell viability assays

For 24–72-h and 6-day experiments, cells were seeded into 96-well plates in a density of 5000 and 2500 cells/well, respectively, except for neonatal dermal fibroblast NDF-2 and NDF-3 (2000 cells/well for 72-h experiments) and Tet21N cells (700 cells/well for 6-day experiments). After the incubation time, cell viability was measured. Performing MTT assay, thiazolyl blue tetrazolium bromide (#M2128, Sigma-Aldrich, St. Louis, MO, USA) was added to each well to reach the final concentration of 0.455 mg/ml. After 3-h incubation under standard conditions, the medium was replaced with 200 μL of DMSO to solubilize the formazan crystals. The absorbance of each well was determined by Sunrise Absorbance Reader (Tecan, Männedorf, Switzerland). For CellTiter-Glo® assay (#G7571, Promega, Madison, WI, USA), cells were seeded in Corning® 96-well Flat Clear Bottom White Polystyrene TC-treated luminescent microplates (#3610, Corning, Corning, NY, USA) and manufacturer instructions were followed. Luminescence was measured by Synergy™ 2 microplate reader (BioTek Instruments, Winooski, VT, USA).

Absolute half maximal inhibitory concentration (IC_50_) of drugs and inhibitors was determined from non-linear regression of datasets of MTT assay and CellTiter-Glo® assay with individual tested concentrations normalized to untreated control cells. Non-linear regression with variable slope was calculated using GraphPad Prism 8.0.2. software (GraphPad Software, San Diego, CA, US). Determined parameters were then used to calculate absolute IC_50_ values according to the following formula: relative IC_50_*(((50-top)/(bottom-50))^(−1/hill slope)).

### Growth analysis

Cells were seeded into 96-well plates (CHLA-15, CHLA-20: 5000 cells/well; NDF-3: 2000 cells/well) and treated the day after seeding with respective drugs. To validate that 2.25 μM DOXY shows no cytotoxic and cytostatic activity in Tet21N cells during the 6-day treatment, Tet21N cells were seeded at low density in 6-well plates and replenished with fresh media w/o DOXY (*MYCN*-on) or with 2.25 μM DOXY (*MYCN*-off) the day after seeding. Subsequently, cell confluency was determined every 4 h using live cell imaging system Incucyte® SX1 (Sartorius, Göttingen, Germany) and plotted relative to initial values. Cell line doubling times were calculated by GraphPad Prism 8.0.2. software using non-linear regression – exponential (Malthusian) growth model.

### Sphere formation assay

Cells were harvested and dissociated into single-cell suspension using Accutase (#LM-T1735, Biosera) and seeded into ultra-low attachment 6-well plates (#CLS3471-24EA, Corning) at a density of 1000 cells/well in a defined serum-free medium: DMEM/F12 based (as detailed in Supplementary Table 1) w/o fetal bovine serum, supplemented with 1× B27 w/o vitamin A (#12587, Gibco), 10 ng/ml EGF (#E9644, Sigma-Aldrich), and 20 ng/ml FGF2 (#SRP4037, Sigma-Aldrich). Cells were replenished with 200 μl of the freshly prepared medium three times a week. The number of spheres (diameter ≥ 50 μm) was manually counted after 21 days of incubation using PROView software analysis of images taken under IM-3 light microscope equipped with C-B5 digital camera (all Optika, Ponteranica, Italy).

### Tumorigenicity assay in vivo

Cells were harvested, enzymatically dissociated and a single cell suspension of 1 × 10^6^ cells in 100 μl of pure DMEM/F/12 medium (#LM-D-1224, Biosera) was injected subcutaneously into the right flank of 9-week-old female NSG (NOD/ShiLtSz-*scid/Il2rγ*^null^) mice. All animal experiments were conducted in accordance with a study (MSMT-4408/2016-6) approved by the Institutional Animal Care and Use Committee of Masaryk University and registered by the Ministry of Education, Youth and Sports of the Czech Republic as required by national legislation. After 29 days, the mice were sacrificed and surgically examined. The xenograft tumors were excised and photographed, and the final tumor volume was determined using the following formula: tumor volume (mm^3^) = length (mm) × width (mm) × width (mm) × 1/2. The adequate sample size (*n* = 3 animals/experimental arm) was chosen based on our previous studies [24, 78] in accordance with the reduction principle. Animal exclusion criteria, randomization, and blinding were not applied in this study.

### Western blotting

Whole-cell extracts were collected using RIPA lysis buffer (2 mM EDTA, 1% IGEPAL® CA-630, 0.1% SDS, 8.7 mg/ml sodium chloride, 5 mg/ml sodium deoxycholate, 50 mM Tris-HCl) supplemented with cOmplete™ Mini Protease Inhibitor Cocktail (#11836170001, Roche, Basel, Switzerland) and PhosSTOP (#4906837001, Roche). 20 µg of total proteins were resolved on 10% polyacrylamide gels (except for 6% gels used for PERK detection) and blotted onto PVDF membranes (#1620177, Bio-Rad Laboratories, Hercules, CA, USA). The membranes were blocked with 5% not-fat dry milk or bovine serum albumin (#A7906, Sigma-Aldrich) in Tris-buffered saline with 0.05% Tween-20 (#93773, Sigma-Aldrich) for at least 1 h and incubated with primary antibodies overnight on rocking platform at 4 °C. The incubation with secondary HRP-linked antibody was conducted at RT for at least 1 h. The list of antibodies used, including dilutions and respective blocking agents, is provided in Supplementary Table 4. Chemiluminescent detection was performed following a 5-min incubation with ECL™ Prime Western Blotting Detection Reagent (#RPN2236, Cytiva, Marlborough, MA, USA) using either Azure C600 imaging system (Azure Biosystems, Dublin, CA, USA) or light sensitive films (#CP-BU NEW 100 NIF, Agfa, Mortsel, Belgium).

Densitometric analysis of western blotting images was done using gel analysis tool in ImageJ (Fiji) software (NIH, Bethesda, MD, USA), version 2.1.0/1.53c. The signal of a protein of interest was normalized to that of a loading control, α-tubulin, β-actin, or GAPDH, detected on the same gel. For all relevant figures, original uncropped blots including all replicates are provided as supplementary Original Data file.

### Immunostaining

Cells were seeded on coverslips coated with Matrigel (#734-1440, Corning). After the incubation period, the coverslips were rinsed by PBS and cells were fixed by 3% paraformaldehyde (#158127, Sigma-Aldrich). The cells were then permeabilized using 0.2% Triton X-100 (#04807423, MP Biomedicals, Irvine, CA, USA) for 1 min and blocking was performed by 3% bovine serum albumin (#A7906, Sigma-Aldrich) for 10 min at RT. The primary and secondary antibodies (Supplementary Table 4) were diluted in blocking solution. The incubation with antibodies lasted for at least 60 min. Nuclei were stained by TO-PRO3 (#T3605, Invitrogen, Carlsbad, CA, USA). The cells were mounted by ProLong™ Diamond Antifade (#P36961, Invitrogen) and imaged using Leica SP8 confocal microscope (Leica, Wetzlar, Germany). Z-stacked images were captured and processed as maximum intensity projections using software LAS X (Leica, 3.4.218368).

### Mitochondrial morphology analysis

Mitochondrial morphology was determined from maximum intensity projection of z-stack confocal images of TOMM20 fluorescence channel using ImageJ (Fiji) plug-in tool MiNA (Mitochondrial Network Analysis) [79]. The pre-processing parameters were applied as follows: 3rd-order median filter with radius 1; 1st-order unsharp mask with radius 2 and mask weight 0.9; contrast enhancement using 2nd-order CLAHE with block size 127 pixels, histogram bins 256 and maximum slope 3.

### Flow cytometry

For all experiments, cells were harvested using Accutase (#LM-T1735, Biosera) and diluted in PBS with 3% fetal bovine serum (#FB-1101, Biosera) and 2 mM EDTA (#ED2SS, Sigma-Aldrich) and immediately processed for measurements using CytoFLEX S flow cytometer (Beckman Coulter, Brea, CA, USA). Cell viability was measured following 15-min incubation with 5 nM SYTOX™ Red Dead Cell Stain (#S34859, Invitrogen) on ice as per manufacturer’s instructions.

Mitochondrial membrane potential was assessed by 10 μg/ml JC-1 Probe (#65-0851-38, Invitrogen) after 10-min incubation at 37 °C. Fluorescence of JC-1 monomers and aggregates was excited by 488 nm laser and detected using FITC (525/40) and ECD (610/20) channels, respectively. To eliminate the spillover signal of JC-1 monomers marking depolarized mitochondria into ECD channel, fluorescence compensation was performed as previously described [80].

To analyze DOXY absorption by neonatal dermal fibroblasts, NDF-3 cells were treated with different concentrations of DOXY for 72 h. DOXY fluorescence was excited by 405 nm laser and detected using the KO525 (550/40) channel [81].

### RT-qPCR

RNA isolation, reverse transcription, and qPCR were performed as previously described [78]. The list of primer sequences used is provided in Supplementary Table 5. The qPCR reactions were performed in technical triplicates.

### Statistical analysis

All experiments were replicated at least three times, as detailed in the figure legends. For violin plots and bar graphs, individual data points show independent biological replicates. For all violin plots, median and quartiles are shown. Bar graphs and line graphs are presented as mean ± standard deviation (SD). Statistical analysis was performed using GraphPad Prism 8.0.2. software. Unpaired two-tailed Student’s t-test was applied when comparing 2 groups, otherwise one-way ANOVA followed by Tukey’s multiple comparison test was used, assuming normal data distribution and similar variance between the compared groups. Linear correlation between 2 datasets was tested by Pearson correlation coefficient (r). *p* values < 0.05 were considered statistically significant; *p < 0.05, **p < 0.01, ***p < 0.001, #*p* < 0.0001.

### Supplementary information

Supplementary Information

Supplementary Video 1

Supplementary Video 2

Original Data File

Reproducibility Checklist

## Data Availability

The data analyzed during this study are included in this published article and the supplemental data files. Additional supporting data are available from the corresponding author upon reasonable request.
