# Supplementary material for: Mitoribosomal synthetic lethality overcomes multidrug resistance in MYC-driven neuroblastoma
Source: Cell Death Dis. 2023 Nov 16;14(11):747. doi: 10.1038/s41419-023-06278-x (PMC10654511; doi:10.1038/s41419-023-06278-x)
Supplement: Supplementary file 1 — Supplementary Information [file 41419_2023_6278_MOESM1_ESM.pdf]

SUPPLEMENTARY INFORMATION

Mitoribosomal synthetic lethality overcomes multidrug resistance in MYC-driven neuroblastoma

Karolina Borankova[1,2], Maria Krchniakova[1,2], Lionel YW Leck[3,4], Adela Kubistova[1], Jakub Neradil[1,2], Patric J Jansson[3,4], Michael D Hogarty[5,6], Jan Skoda[1,2,*]

[1]Department of Experimental Biology, Faculty of Science, Masaryk University, 62500, Brno, Czech Republic
[2]International Clinical Research Center, St. Anne's University Hospital, 65691, Brno, Czech Republic
[3]Cancer Drug Resistance & Stem Cell Program, School of Medical Science, Faculty of Medicine and Health, The University of Sydney, Camperdown, NSW 2006, Australia
[4]Bill Walsh Translational Cancer Research Laboratory, Kolling Institute, Faculty of Medicine and Health, The University of Sydney, St. Leonards, NSW 2065, Australia
[5]Division of Oncology and Center for Childhood Cancer Research, The Children's Hospital of Philadelphia, Philadelphia, PA, USA
[6]Department of Pediatrics, Perelman School of Medicine, University of Pennsylvania, Philadelphia, PA, USA

* Correspondence: jan.skoda@sci.muni.cz

This PDF file includes:

Legends for Supplementary Videos 1,2

Figures S1 to S14

Supplementary Tables 1 to 5

Other Supplementary Materials for this manuscript include the following:

Supplementary Videos 1,2

Original Data file with uncropped blots and replicates

Supplementary Video 1: Mdivi-1-mediated inhibition of DRP1 induces cell death in neuroblastoma. A time-lapse video of CHLA-15 and CHLA-20 neuroblastoma cells treated with vehicle or mdivi-1 in indicated concentrations. Phase contrast images were captured under 10× objective by the live-cell imaging system Incucyte® in 4-h intervals for 72 h.

Supplementary Video 2: DOXY-mediated inhibition of mitochondrial translation induces cell death in neuroblastoma cells but not in nonmalignant neonatal dermal fibroblasts. A time-lapse video of neuroblastoma cells (CHLA-15, CHLA-20) and fibroblasts (NDF-3) treated with vehicle or DOXY in indicated concentrations. Phase contrast images were captured under 10× objective by the live-cell imaging system Incucyte® in 4-h intervals for 72 h.

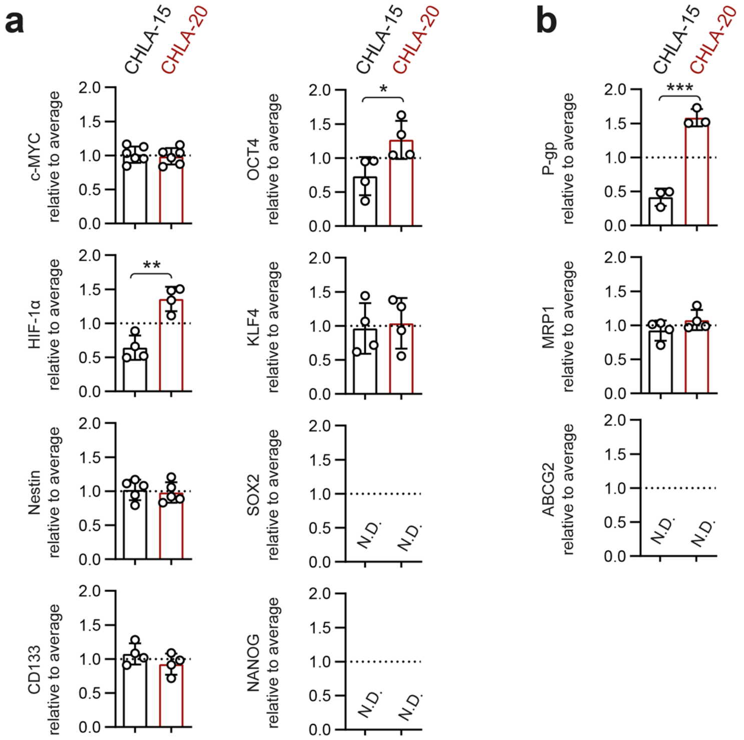

Fig. S1: **Analysis of cancer stemness-related markers revealed upregulation of HIF-1α and P-gp in post-therapy CHLA-20 neuroblastoma cells. a,b**, Densitometric analysis of western blotting detection of stemness transcription factors and cancer stemness-related markers (**a**) and ABC transporters (**b**) in therapy-naïve CHLA-15 and post-therapy CHLA-20 near-isogenic neuroblastoma cells. Normalized protein levels are plotted relative to the average level of both cell lines, mean ± SD; N.D. – not detected. Statistical significance was determined by unpaired two-tailed Student's t-test, **p*<0.05, **p<0.01, ***p<0.001.

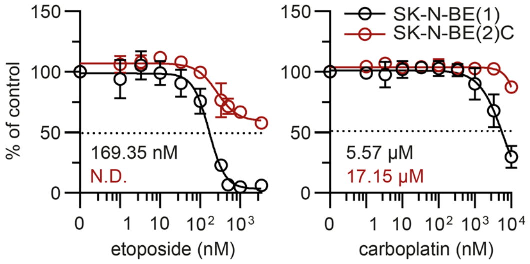

Fig. S2: Drug sensitivity in therapy-naïve SK-N-BE(1) and post-therapy SK-N-BE(2)C near-isogenic cell lines. CellTiter-Glo cell viability assay analysis of therapy-naïve SK-N-BE(1) and post-therapy SK-N-BE(2)C after 72-h treatment showed enhanced resistance of SK-N-BE(2)C to conventional chemotherapeutics, etoposide and carboplatin. Data are presented as mean ± SD, biological n=3, technical n=3.Calculated IC_{50} are indicated. N.D.- IC_{50} not determinable.

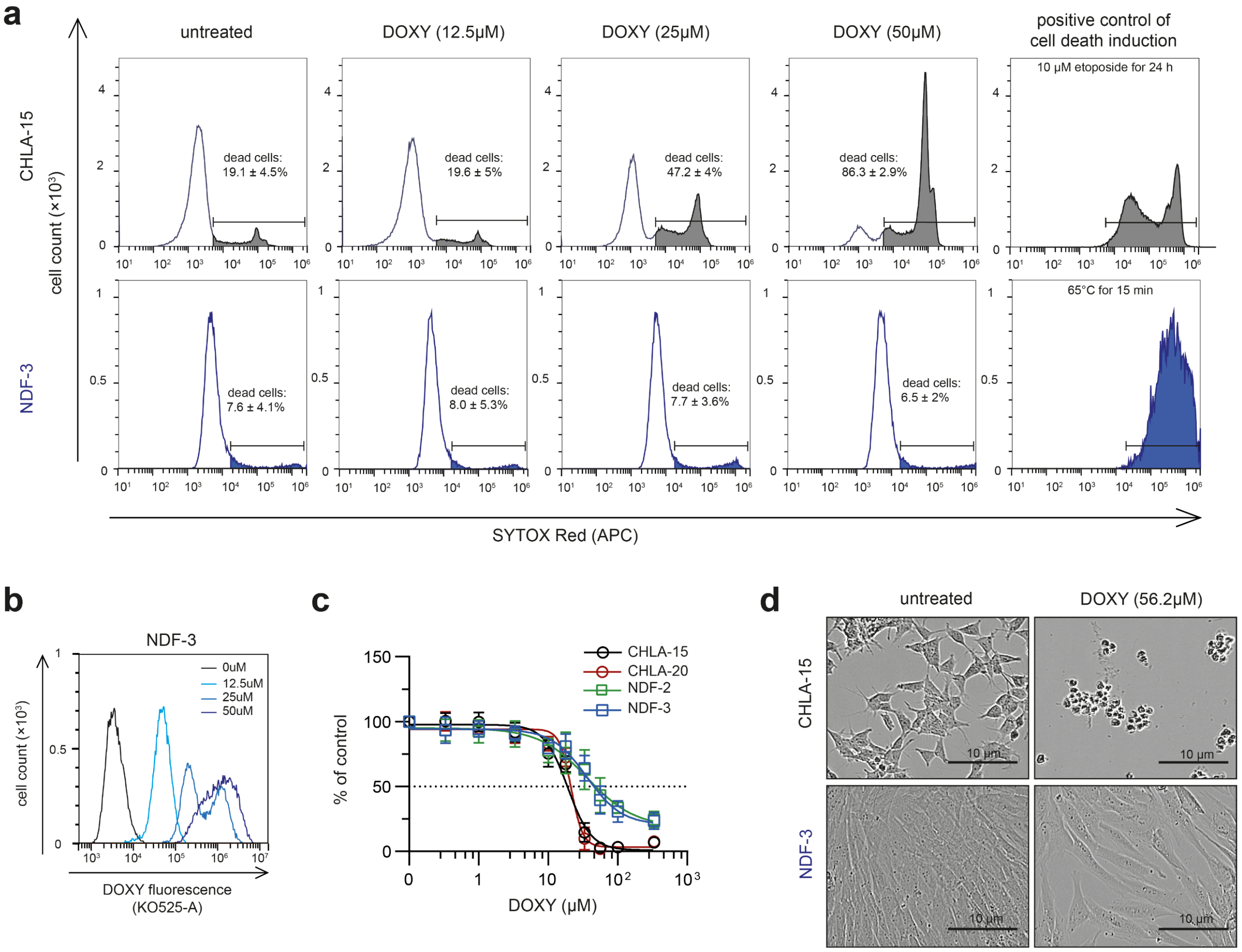

Fig. S3: **DOXY-mediated inhibition of mitochondrial translation does not induce cell death in neonatal fibroblasts. a**, Flow-cytometry analysis of cell viability after DOXY treatment for 72 h using SYTOX Red staining showed marked dose-dependent increase of dead cells in CHLA-15 neuroblastoma cells, whereas the viability of NDF-3 neonatal fibroblasts was not affected by the treatment. The percentages of SYTOX Red-positive dead cells are presented as mean ± SD, biological n=3**. b**, Despite having no effects on cell viability, absorption of DOXY by NDF-3 cells was confirmed by flow cytometry analysis that revealed a dose-dependent increase of DOXY fluorescence. Representative histograms are shown, biological n=2. **c,** MTT cell viability assay analysis showed that DOXY treatment limits proliferation of NDF-2 and NDF-3 neonatal dermal fibroblasts to a lesser extent compared with the CHLA-15/CHLA-20 pair. Data are presented as mean ± SD, biological n≥5, technical n≥3. The microscopic observations after 72-h treatment with indicated DOXY concentration **(d)** together with the viability analysis **(a)** indicate that DOXY-induced reduction of MTT absorbance was caused by reduction of cell growth rather than induction of cell death. See also Supplementary Video 2.

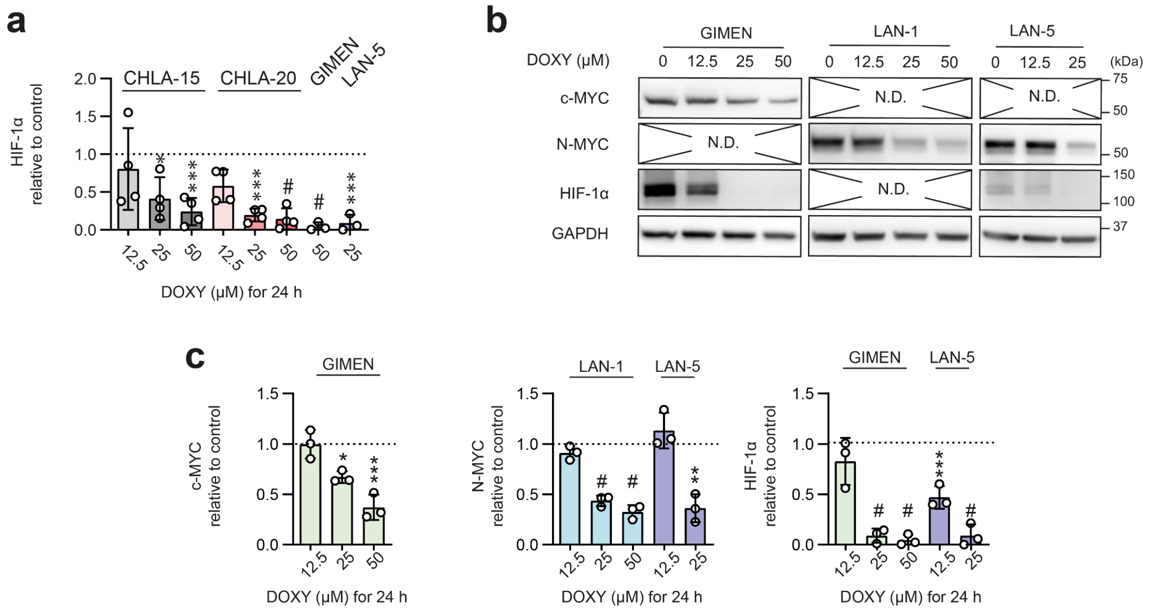

Fig. S4: Transcription factors associated with poor neuroblastoma prognosis are downregulated upon DOXY-mediated inhibition of mitochondrial translation. a, Densitometric analysis of western blotting detection showed significant decrease of HIF-1α across a panel of neuroblastoma cells after 24-h DOXY treatment in indicated concentrations. **b,c,** Western blotting (**b**) and densitometric analysis (**c**) showed downregulation of transcription factors c-MYC, N-MYC and HIF-1α upon 25μM and 50μM DOXY treatment for 24 h in GIMEN, LAN-1, and LAN-5 neuroblastoma cells. Normalized protein levels are plotted relative to untreated controls, mean ± SD. N.D. **-** not detectable at this cell line**.** Statistical significance was determined by one-way ANOVA followed by Tukey's multiple comparisons test (**a,c**), *p<0.05, **p<0.01, ***p<0.001, #p<0.0001.

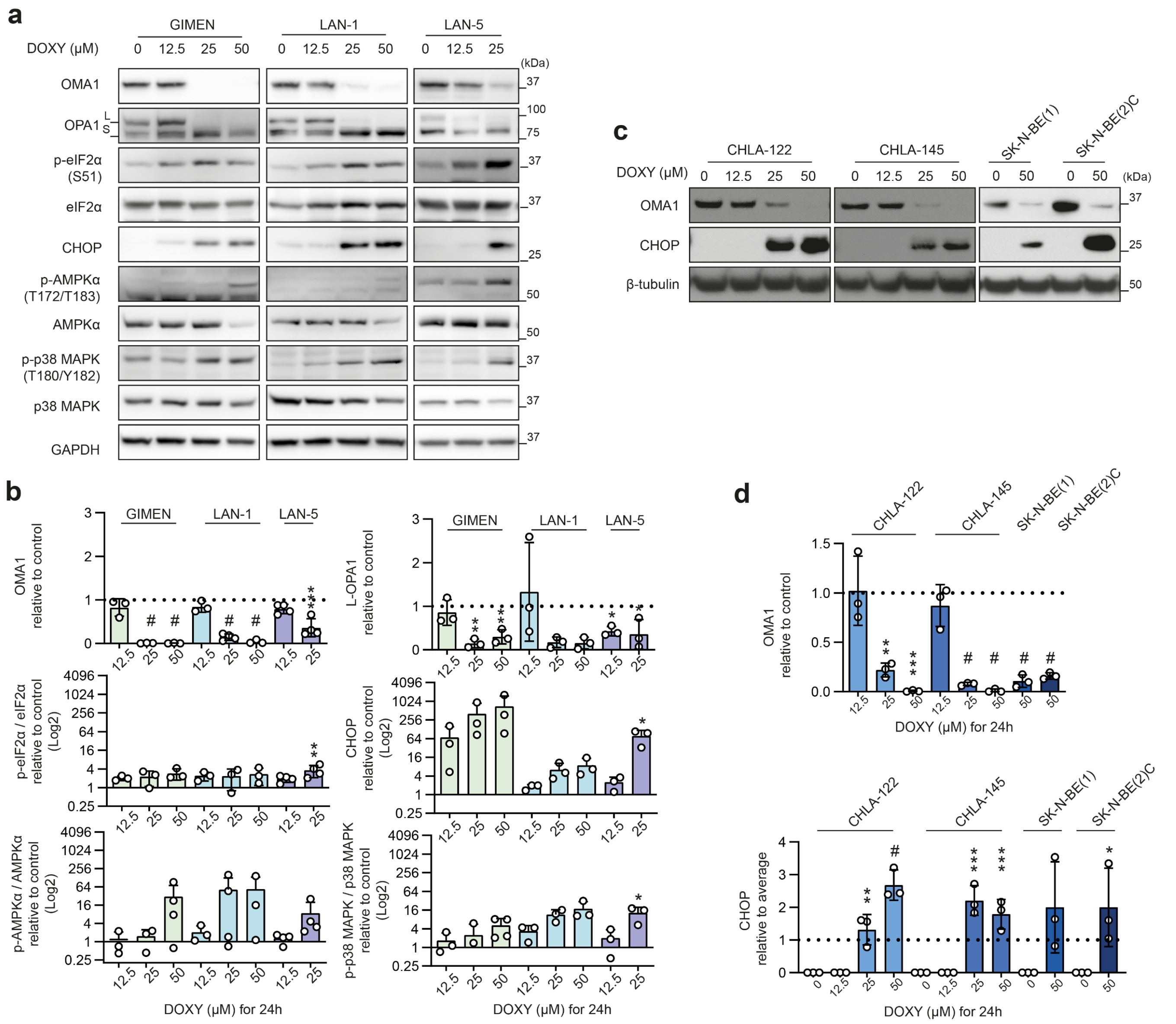

Fig. S5: OMA1-mediated ISR is consistently activated upon DOXY-mediated inhibition of mitochondrial translation across a panel of neuroblastoma cells. a-d, Western blotting and subsequent densitometric analysis of markers of the mitochondrial stress and ISR showed consistent effects of DOXY-mediated inhibition of mitochondrial translation across the neuroblastoma models: GIMEN, LAN-1, LAN-5 (**a,b**), CHLA-122, CHLA-145, SK-N-BE(1), SK-N-BE(2)C (**c,d**). Activating phosphorylation of AMPKα and p38 MAPK were also analyzed after 24-h treatment with indicated concentrations of DOXY in GIMEN LAN-1, LAN-5 (**a,b**). Normalized protein levels are plotted relative to untreated controls or in case of CHOP detection in CHLA-122, CHLA-145, SK-N-BE(1), SK-N-BE(2)C relative to average density of analyzed samples, mean ± SD, biological n≥3. Statistical significance was determined by one-way ANOVA followed by Tukey's multiple comparisons test (GIMEN, LAN-1, LAN-5, CHLA-122, CHLA-145) or by unpaired two-tailed Student's t-test (SK-N-BE(1), SK-N-BE(2)C), *p<0.05, **p<0.01, ***p<0.001, #p<0.0001.

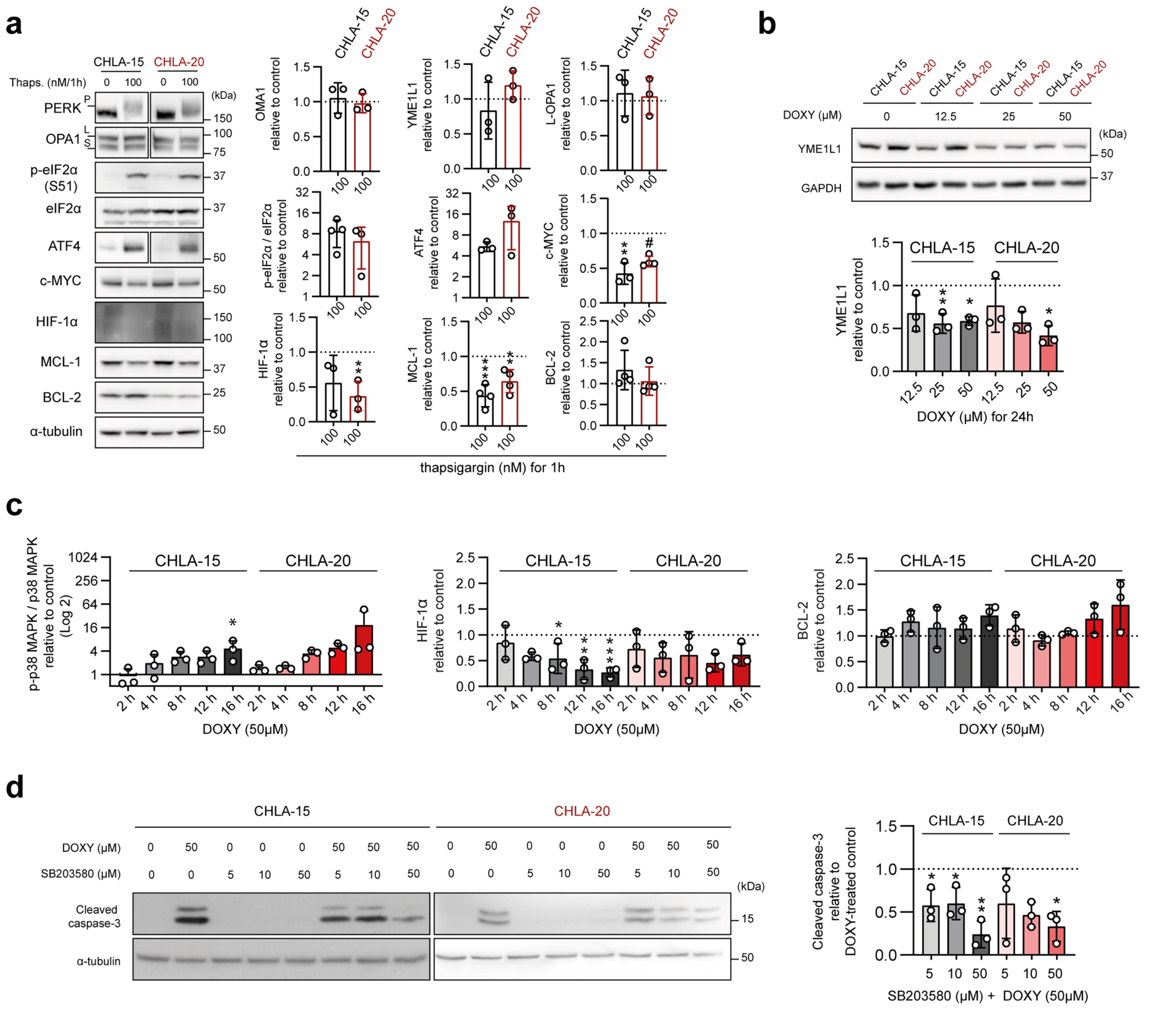

Fig. S6: ER stress does not promote OPA1 processing and DOXY-induced cell death can be partially rescued by p38 MAPK inhibition. a, Western blotting detection and densitometric analysis of markers of the mitochondrial stress and ISR, together with c-MYC, HIF-1α and BCL-2 anti-apoptotic proteins in neuroblastoma cells CHLA-15 and CHLA-20 after PERK-mediated activation of ISR by 100 nM thapsigargin. Note the unprocessed OPA1 and phosphorylated eIF2α in thapsigargin-treated samples. Normalized protein levels are plotted relative to untreated controls, mean ± SD, biological n=3. **b**, A dose-dependent downregulation of YME1L1 detected by western blotting and densitometric analysis in the CHLA-15/CHLA-20 pair after 24-h DOXY treatment. Normalized protein levels are plotted relative to untreated controls, mean ± SD, biological n=3**. c,** Densitometric analysis of western blotting detection of p-p38 MAPK (T180/Y182)/p38 MAPK, HIF1α and BCL-2 in CHLA-15 and CHLA-20 neuroblastoma cells treated with 50 µM DOXY for 2–16 h. Normalized protein levels are plotted relative to untreated controls, mean ± SD. **d,** Western blotting and subsequent densitometric analysis of cleaved caspase-3 levels after 24-h 50µM DOXY treatment either w/o or with inhibitor of p38 MAPK (SB203580) showed that presence of SB203580 reduced the level of detected cleaved caspase-3 induced by DOXY comparing to DOXY alone. Normalized protein levels are plotted relative to DOXY-treated controls, mean ± SD. Statistical significance was determined by unpaired two-tailed Student's t-test (**a**) and by one-way ANOVA followed by Tukey's multiple comparisons test (**b**,**c**,**d**), *p<0.05, **p<0.01, ***p<0.001, #p<0.0001.

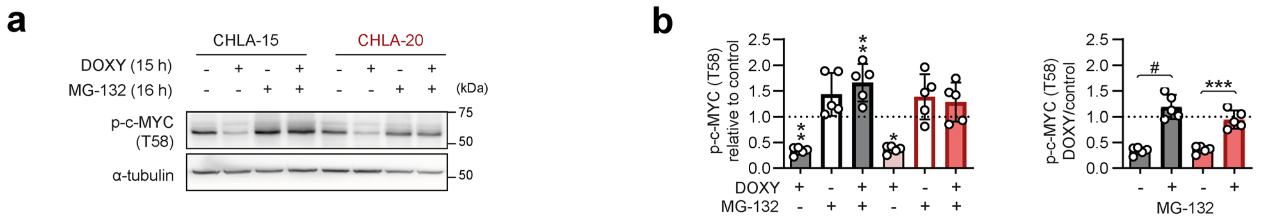

Fig. S7: DOXY-induced p-c-MYC(T58) downregulation is fully rescued by proteasome inhibition. a,b, Proteasome inhibition fully rescues DOXY-induced degradation of p-c MYC(T58) in the CHLA-15/20 pair, as detected by western blotting (**a**) and densitometric analysis (**b**). Cells were pre-treated with 5 µM MG-132 to block proteasome activity and after 1 h, 50 µM DOXY was added for additional 15 h. Left graph, normalized protein levels are plotted relative to fully untreated controls (dashed line). Right graph, ratios of normalized protein levels in DOXY-treated cells (w/o or with MG-132) and respective controls (w/o or with MG-132) are plotted as mean ± SD. Statistical significance was determined by one-way ANOVA followed by Tukey's multiple comparisons test, *p<0.05, **p<0.01, ***p<0.001, #p<0.0001.

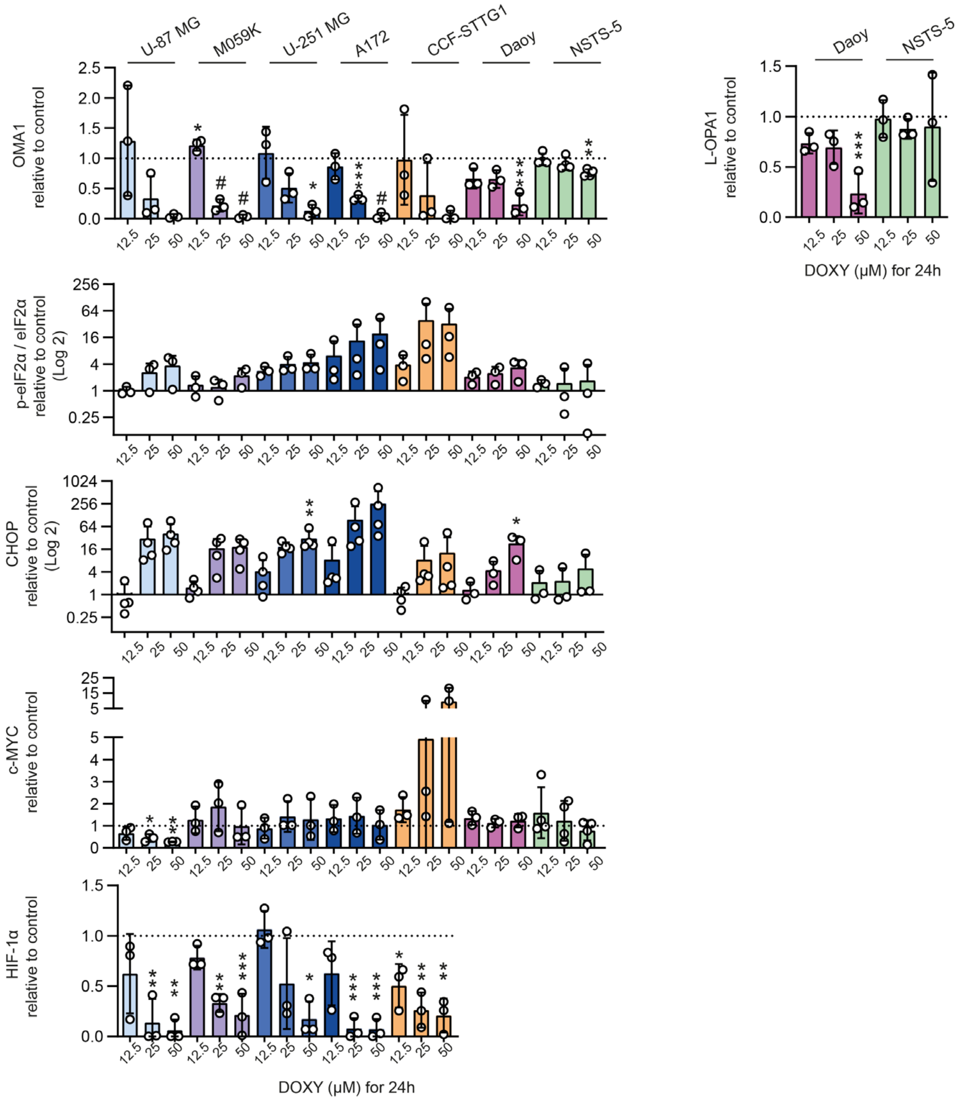

Fig. S8: OMA1-mediated ISR is dose dependently activated by DOXY treatment in various nervous system tumors. Densitometric analysis of western blotting detection of markers of mitochondrial stress, ISR and oncogenic transcription factors, c-MYC and HIF-1α, revealed an induction of mitochondrial stress and ISR after 24-h DOXY treatment in a panel of cell lines derived from different nervous system tumors. As in neuroblastoma, HIF-1α was significantly downregulated in nervous tissue derived tumor types, but a consistent downregulation of c-MYC was not detected. Normalized protein levels are plotted relative to untreated controls, mean ± SD. Statistical significance was determined by one-way ANOVA followed by Tukey's multiple comparisons test, *p<0.05, **p<0.01, ***p<0.001, #p<0.0001.

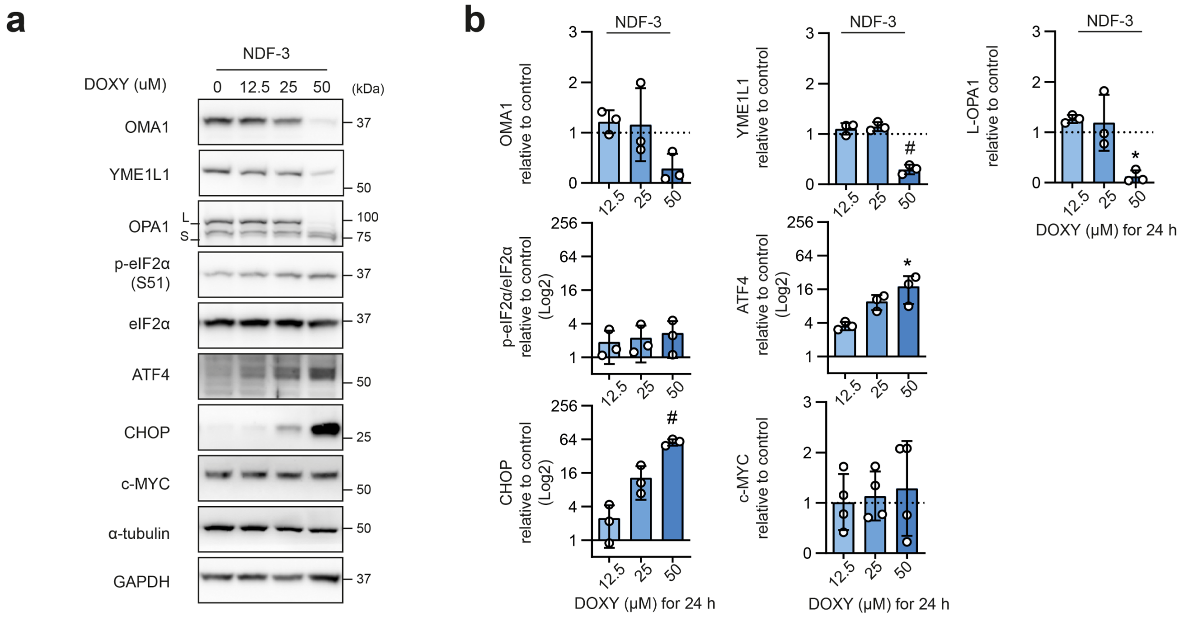

Fig. S9: Inhibition of mitochondrial translation induces OMA1-mediated ISR in human neonatal dermal fibroblasts resistant to DOXY-induced cell death. a,b, Western blotting detection (**a**) and densitometric analysis (**b**) showed induction of mitochondrial stress and ISR but unaffected c-MYC expression upon 24-h DOXY treatment in NDF-3 cells. Normalized protein levels are plotted relative to untreated controls, mean ± SD. Statistical significance was determined by one-way ANOVA followed by Tukey's multiple comparisons test, *p<0.05, #p<0.0001.

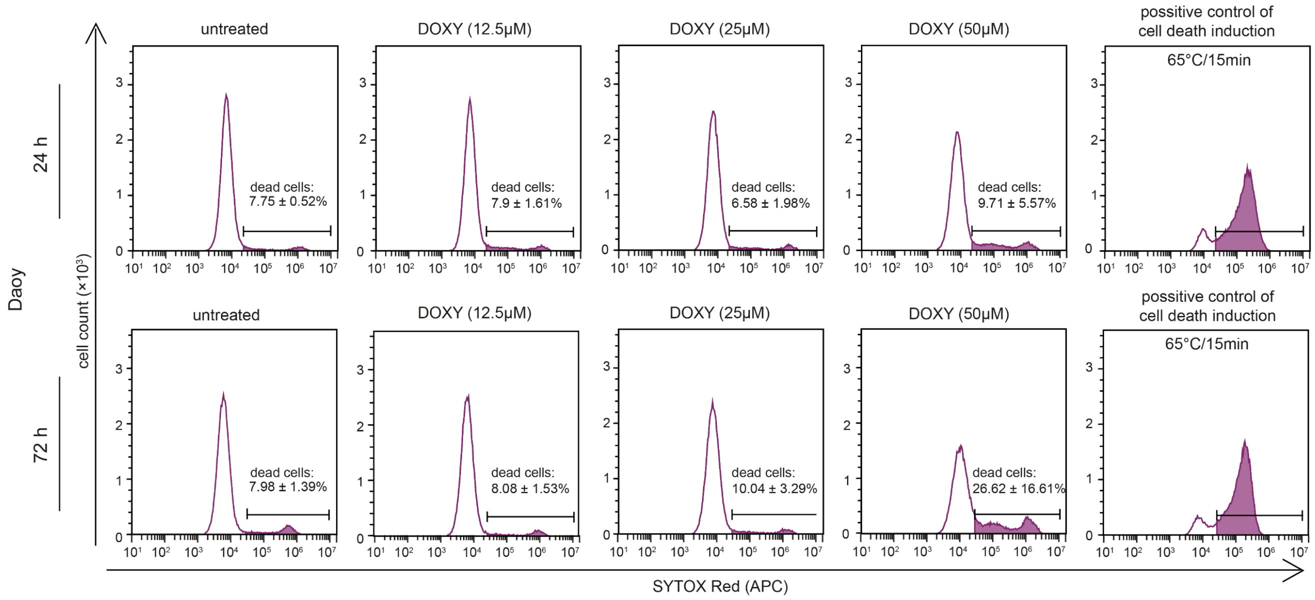

Fig. S10: Daoy medulloblastoma cells show attenuated sensitivity to DOXY-induced cell death. Flow cytometry analysis of cell viability using SYTOX Red staining after DOXY treatment for 24 h and 72 h. The percentages of SYTOX Red-positive dead cells are presented as mean ± SD, biological n=3 (24-h treatment) and n=8 (72-h treatment).

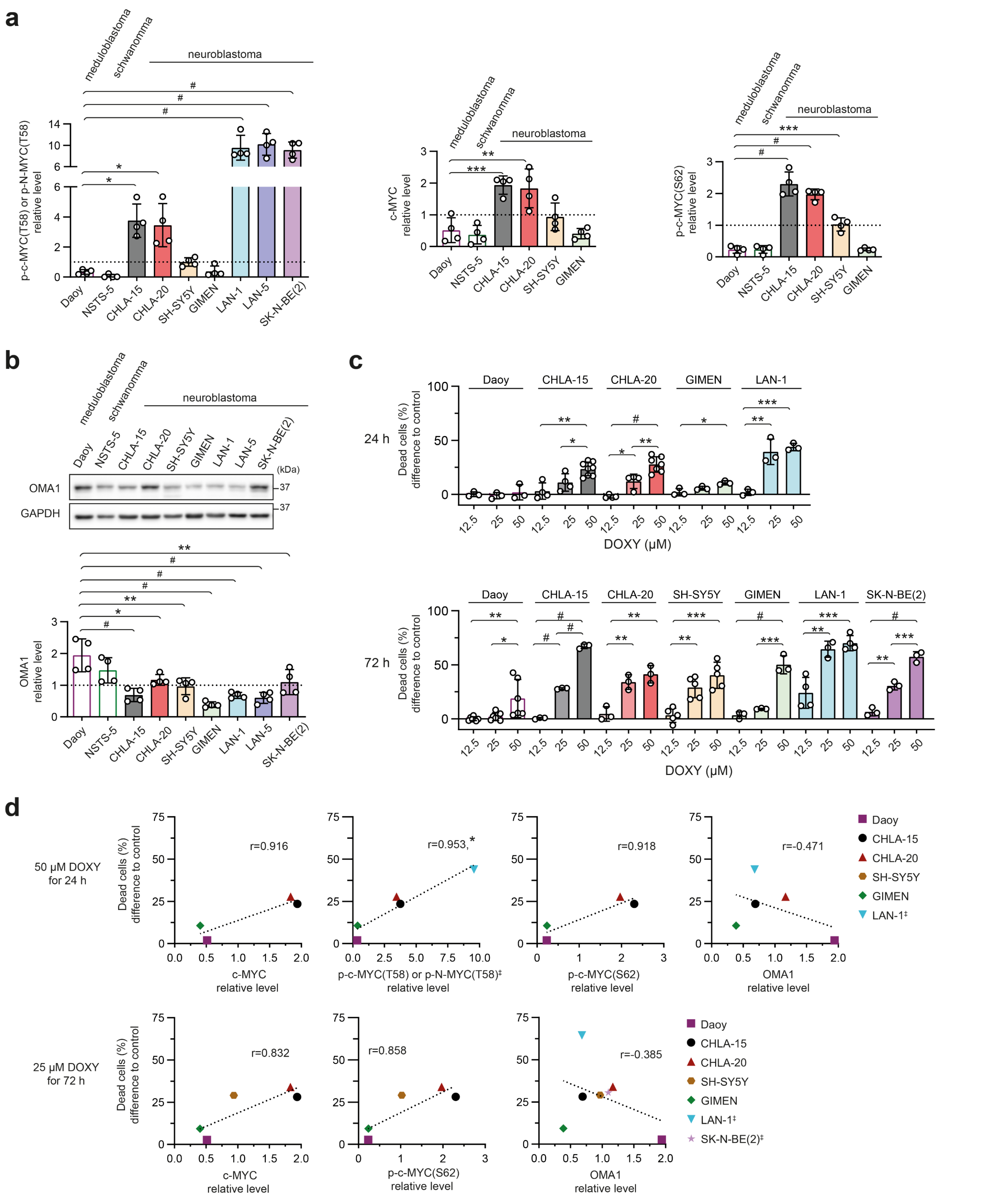

Fig. S11: Susceptibility to DOXY-induced cell death positively correlates with overexpression and phosphorylation of MYC proteins. a-b, Densitometric analysis of western blotting detection of p-CMYC/N-MYC(T58), c-MYC, and p-c-MYC(S62) (**a**) and OMA1 (**b**) in a panel of nervous system tumors. Normalized protein levels are plotted relative to the average level of all samples, mean ± SD. **c**, A compiled comparison of cell death

rate in neuroblastoma models, CHLA-15, CHLA-20, SH-SY5Y, GIMEN, LAN-1, and SK-N-BE(2), and Daoy medulloblastoma cells after DOXY treatment for 24 h and 72 h. Viability analyzed by flow cytometry using SYTOX Red staining is presented as the difference in percentages of SYTOX Red-positive dead cells after indicated treatment vs. respective untreated controls, biological n≥3. **d**, Pearson correlation of c-MYC, p-CMYC/N-MYC (T58), p-c-MYC (S62) and OMA1 levels with difference of dead cells to control after 50 µM DOXY for 24 h and 25 µM for 72 h revealed positive correlation between high c-MYC, p-CMYC/N-MYC (T58), p-c-MYC (S62) levels and susceptibility to DOXY-induced cell death, whereas the sensitivity to DOXY was independent of OMA1 levels, r = Pearson correlation coefficient; ǂ, *MYCN*-amplified. Statistical significance was determined by one-way ANOVA followed by Tukey's multiple comparisons test (**a-c**) and by Pearson correlation (**d**), *p<0.05, **p<0.01, ***p<0.001, #p<0.0001.

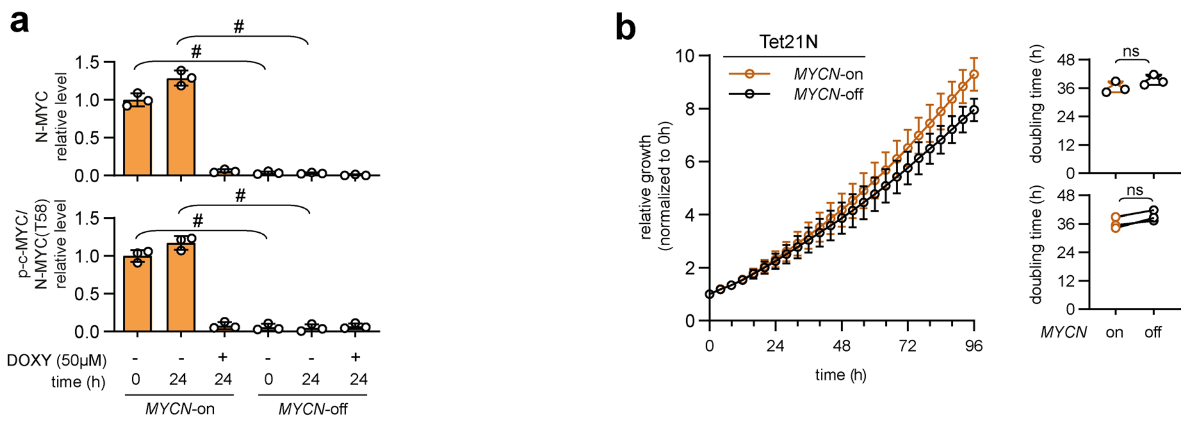

Fig. S12: Tet21N *MYCN*-off cells have extensively downregulated N-MYC level while their growth rate is comparable with Tet21N *MYCN*-on cells. a, Densitometric analysis of western blotting detection of N-MYC and p-CMYC/N-MYC(T58) in Tet21N *MYCN*-on and Tet21N *MYCN*-off cells. Normalized protein levels are plotted relative to the average level of all samples, mean ± SD. **b**, Live-cell imaging growth rate analysis of Tet21N untreated cells (*MYCN*-on) and cells treated with 2.25 µM DOXY to repress *MYCN* expression from the Tet-off construct (*MYCN*-off). Relative growth values are presented as mean ± SD, biological n=3, technical n=3. Statistical significance was determined by one-way ANOVA followed by Tukey's multiple comparisons test (**a**) and by unpaired two-tailed Student's t-test (**b**), ns p>0.05, #p<0.0001.

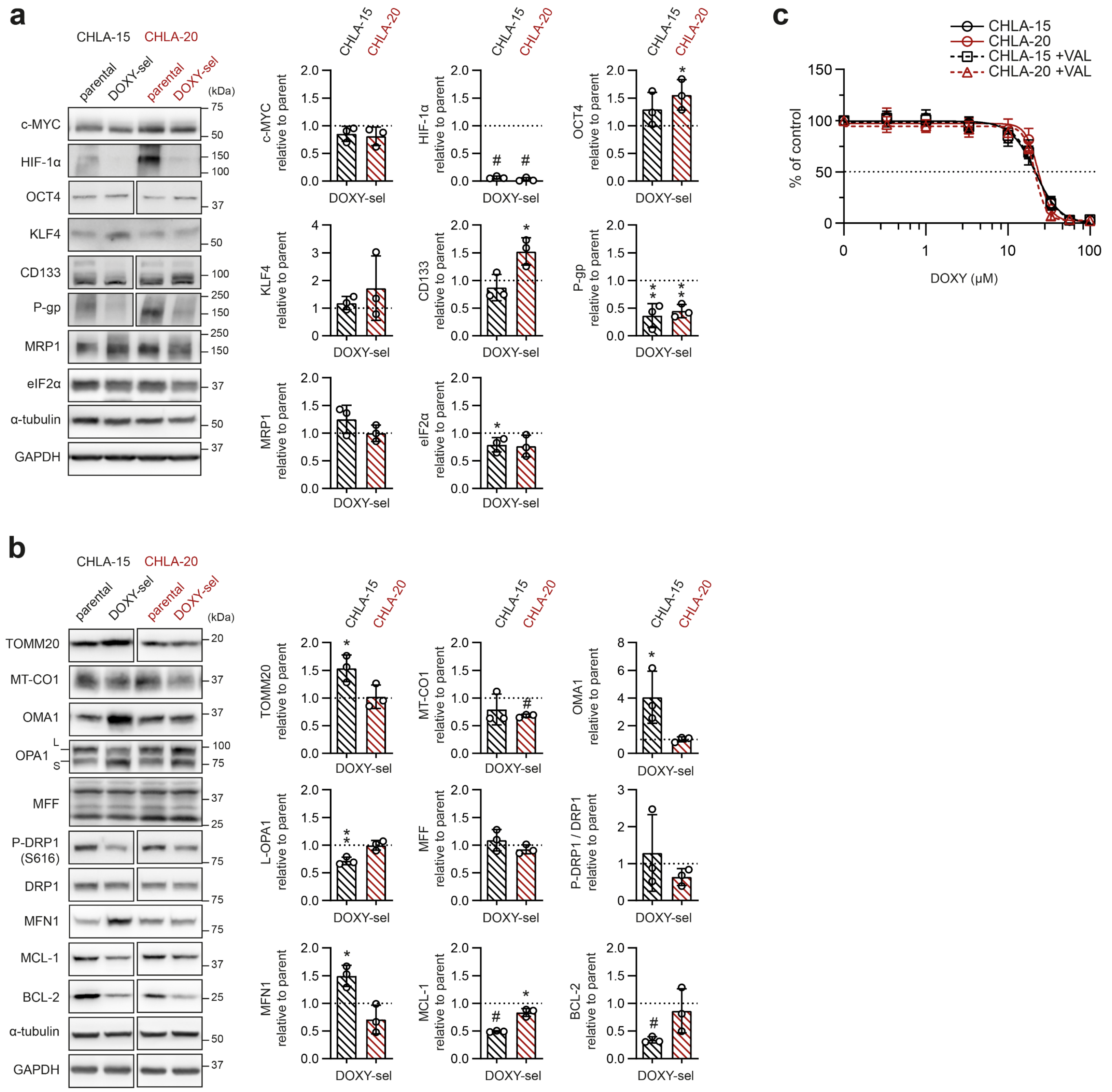

Fig. S13: Protein expression changes in DOXY-sel cell lines. a,b, Western blotting detection and densitometric analysis of stemness-related proteins, drug efflux pumps and eIF2α (**a**) and proteins related to mitochondrial dynamics and mitochondrial apoptotic pathway (**b**) in lysates from cells cultured in the standard medium. Normalized protein levels of DOXY-sel cells are plotted relative to normalized protein levels of parental cells, mean ± SD, biological n=3. **c,** MTT cell viability assay analysis of parent CHLA-15 and CHLA-20 cells after 72-h exposure to DOXY alone or with 0.5 μM of P-gp inhibitor valspodar (VAL). Data are presented as mean ± SD, biological n=5, technical n=3. Statistical significance was determined by unpaired two-tailed Student's t-test (**a,b**), *$p<0.05$, **$p<0.01$, #$p<0.0001$.

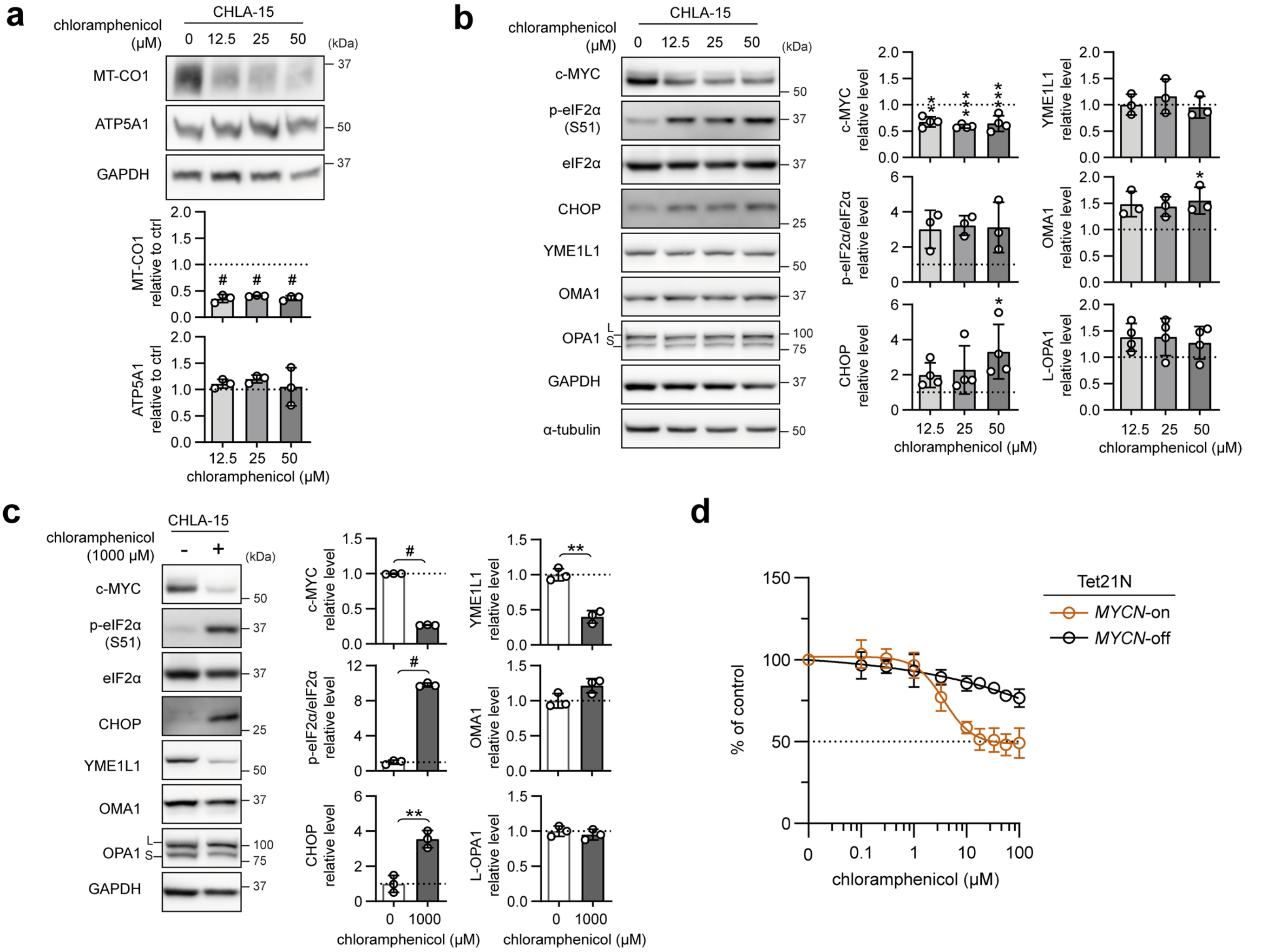

Fig. S14: N-MYC levels governs sensitivity to chloramphenicol-mediated inhibition of mitochondrial translation and ISR activation. a, Expression of mitochondrial proteins, mitochondrial-encoded MT-CO1 and nuclear-encoded ATP5A1, analyzed by western blotting and subsequent densitometry after 24-h chloramphenicol treatment. Normalized protein levels are plotted relative to untreated controls, mean ± SD. **b,c**, Western blotting detection and densitometric analysis showed induction of mitochondrial stress-activated ISR and downregulation of c-MYC upon 72-h chloramphenicol treatment in CHLA-15 cells. Normalized protein levels are plotted relative to untreated controls, mean ± SD (b) or relative to the average level of both samples, mean ± SD (c). **d,** MTT cell viability assay analysis after 6-day treatment showed lowered sensitivity of Tet21N *MYCN*-off cells to chloramphenicol compared with their *MYCN*-on counterparts. Data are presented as mean ± SD, biological n=5, technical n=3. Statistical significance was determined by one-way ANOVA followed by Tukey's multiple comparisons test (**a,b**) and by unpaired two-tailed Student's t-test (**c**), *p<0.05, **p<0.01, ***p<0.001, #p<0.0001.

Supplementary Table 1. Summary of neuroblastoma cell lines and culture media composition

Cell Line	Cellosaurus accession #	Genetic Background	Source	Culture Medium	Supplements	Buffer System
CHLA-15	CVCL_6594	*MYC* amplified, *ALK* R1275Q	COG/ALSF Childhood Cancer Repository; www.cccells.org	DMEM/F-12 (LM-D1224, Biosera)	10% FBS (FB-1101); 2 mM L-Glutamine (XC-T1715); 1× MEM Non-Essential Amino Acids (XC-E1154); Penicilin (100 IU/mL)/Streptomycin (100 µg/ml) (XC-A4122) all Biosera; 0.12% ITS-X (51500056, Gibco)	15 mM HEPES & 1.2 g/l sodium bicarbonate (included in the culture medium)
CHLA-20	CVCL_6602	*MYC* amplified, *ALK* R1275Q	COG/ALSF Childhood Cancer Repository; www.cccells.org	DMEM/F-12 (LM-D1224, Biosera)	10% FBS (FB-1101); 2 mM L-Glutamine (XC-T1715); 1× MEM Non-Essential Amino Acids (XC-E1154); Penicilin (100 IU/mL)/Streptomycin (100 µg/ml) (XC-A4122) all Biosera; 0.12% ITS-X (51500056, Gibco)	15 mM HEPES & 1.2 g/l sodium bicarbonate (included in the culture medium)
CHLA-122	CVCL_A054	*MYCN* amplified	COG/ALSF Childhood Cancer Repository; www.cccells.org	DMEM/F-12 (LM-D1224, Biosera)	10% FBS (FB-1101); 2 mM L-Glutamine (XC-T1715); 1× MEM Non-Essential Amino Acids (XC-E1154); Penicilin (100 IU/mL)/Streptomycin (100 µg/ml) (XC-A4122) all Biosera; 0.12% ITS-X (51500056, Gibco)	15 mM HEPES & 1.2 g/l sodium bicarbonate (included in the culture medium)
CHLA-136	CVCL_6590	*MYCN* amplified	COG/ALSF Childhood Cancer Repository; www.cccells.org	DMEM/F-12 (LM-D1224, Biosera)	10% FBS (FB-1101); 2 mM L-Glutamine (XC-T1715); 1× MEM Non-Essential Amino Acids (XC-E1154); Penicilin (100 IU/mL)/Streptomycin (100 µg/ml) (XC-A4122) all Biosera; 0.12% ITS-X (51500056, Gibco)	15 mM HEPES & 1.2 g/l sodium bicarbonate (included in the culture medium)
CHLA-145	CVCL_AQ13	*MYCN* amplified	COG/ALSF Childhood Cancer Repository; www.cccells.org	DMEM/F-12 (LM-D1224, Biosera)	10% FBS (FB-1101); 2 mM L-Glutamine (XC-T1715); 1× MEM Non-Essential Amino Acids (XC-E1154); Penicilin (100 IU/mL)/Streptomycin (100 µg/ml) (XC-A4122) all Biosera; 0.12% ITS-X (51500056, Gibco)	15 mM HEPES & 1.2 g/l sodium bicarbonate (included in the culture medium)
SK-N-BE(1)	CVCL_9898	*MYCN* amplified	COG/ALSF Childhood Cancer Repository; www.cccells.org	DMEM/F-12 (LM-D1224, Biosera)	10% FBS (FB-1101); 2 mM L-Glutamine (XC-T1715); 1× MEM Non-Essential Amino Acids (XC-E1154); Penicilin (100 IU/mL)/Streptomycin (100 µg/ml) (XC-A4122) all Biosera; 0.12% ITS-X (51500056, Gibco)	15 mM HEPES & 1.2 g/l sodium bicarbonate (included in the culture medium)
SK-N-BE(2)C	CVCL_0529	*MYCN* amplified	COG/ALSF Childhood Cancer Repository; www.cccells.org	DMEM/F-12 (LM-D1224, Biosera)	10% FBS (FB-1101); 2 mM L-Glutamine (XC-T1715); 1× MEM Non-Essential Amino Acids (XC-E1154); Penicilin (100 IU/mL)/Streptomycin (100 µg/ml) (XC-A4122) all Biosera; 0.12% ITS-X (51500056, Gibco)	15 mM HEPES & 1.2 g/l sodium bicarbonate (included in the culture medium)
SH-SY5Y	CVCL_0019	-	ECACC	DMEM/F-12 (LM-D1224, Biosera)	20% FBS (FB-1101); 2 mM L-Glutamine (XC-T1715); 1× MEM Non-Essential Amino Acids (XC-E1154); Penicilin (100 IU/ml) /Streptomycin (100 µg/ml) (XC-A4122) all Biosera	15 mM HEPES & 1.2 g/l sodium bicarbonate (included in the culture medium)
SK-N-BE(2)	CVCL_0528	*MYCN* amplified	ECACC	DMEM/F-12 (LM-D1224, Biosera)	20% FBS (FB-1101); 2 mM L-Glutamine (XC-T1715); 1× MEM Non-Essential Amino Acids (XC-E1154); Penicilin (100 IU/ml) /Streptomycin (100 µg/ml) (XC-A4122) all Biosera	15 mM HEPES & 1.2 g/l sodium bicarbonate (included in the culture medium)
GIMEN	CVCL_1232	-	Kind gift of Prof. Krejčí (Masaryk University, Brno, Czech Republic)	RPMI-1640, (PM-R1645, Biosera)	10% FBS (FB-1101); 2 mM L-Glutamine (XC-T1715); 1× MEM Non-Essential Amino Acids (XC-E1154); Penicilin (100 IU/ml) /Streptomycin (100 µg/ml) (XC-A4122) all Biosera	15 mM HEPES (H0887) & 1.2 g/l sodium bicarbonate (S8761), both Sigma-Aldrich
LAN-1	CVCL_1827	*MYCN* amplified	Kind gift of Prof. Krejčí (Masaryk University, Brno, Czech Republic)	RPMI-1640, (PM-R1645, Biosera)	10% FBS (FB-1101); 2 mM L-Glutamine (XC-T1715); 1× MEM Non-Essential Amino Acids (XC-E1154); Penicilin (100 IU/ml) /Streptomycin (100 µg/ml) (XC-A4122) all Biosera	15 mM HEPES (H0887) & 1.2 g/l sodium bicarbonate (S8761), both Sigma-Aldrich
LAN-5	CVCL_0389	*MYCN* amplified	Kind gift of Prof. Krejčí (Masaryk University, Brno, Czech Republic)	RPMI-1640, (PM-R1645, Biosera)	10% FBS (FB-1101); 2 mM L-Glutamine (XC-T1715); 1× MEM Non-Essential Amino Acids (XC-E1154); Penicilin (100 IU/ml) /Streptomycin (100 µg/ml) (XC-A4122) all Biosera	15 mM HEPES (H0887) & 1.2 g/l sodium bicarbonate (S8761), both Sigma-Aldrich
Tet21N	CVCL_9812	N-MYC expression from a *MYCN* Tet-off construct	Kindly provided by Dr. Westermann (DKFZ, Heidelberg, Germany)	RPMI-1640, (PM-R1645, Biosera)	10% FBS (FB-1101); 2 mM L-Glutamine (XC-T1715); Penicilin (100 IU/ml) /Streptomycin (100 µg/ml) (XC-A4122) all Biosera, 0.2 mg/ml Geneticin™ G418 Sulfate (11811023, Gibco), 0.09 mg/ml Hygromycin B (R.CP12.1, Carl Roth)	15 mM HEPES (H0887) & 1.2 g/l sodium bicarbonate (S8761), both Sigma-Aldrich

Providers: Carl Roth (Karlsruhe, Germany); ECACC, The European Collection of Authenticated Cell Cultures, UK Health Security Agency); Gibco (Carlsbad, CA, USA); Sigma-Aldrich (St. Louis, MO, USA). FBS, fetal bovine serum; ITS-X, Insulin-Transferrin-Selenium-Ethanolamine; HEPES, N-(2-Hydroxyethyl)piperazine-N′-(2-ethanesulfonic acid), CAS #7365-45-9.

Supplementary Table 2. Summary of non-neuroblastoma cell lines and culture media composition

Cell type	Cell Line	Cellosaurus accession #	Source	Culture Medium	Supplements	Buffer System
medulloblastoma	Daoy	CVCL_1167	ECACC	DMEM Low Glucose (PM-D1105, Biosera)	10% FBS (FB-1101); 2 mM L-Glutamine (XC-T1715); 1× MEM Non-Essential Amino Acids (XC-E1154); Penicilin (100 IU/ml) /Streptomycin (100 µg/ml) (XC-A4122) all Biosera	15 mM HEPES (H0887) & 1.2 g/l sodium bicarbonate (S8761), both Sigma-Aldrich
schwannoma	NSTS-5	In-house derived (under IGA MZCR NR/9125-4 project; ethics approval no. 23/2005)			20% FBS (FB-1101); 2 mM L-Glutamine (XC-T1715); 1× MEM Non-Essential Amino Acids (XC-E1154); Penicilin (100 IU/ml) /Streptomycin (100 µg/ml) (XC-A4122) all Biosera	
glioblastoma	U-87 MG	CVCL_0022	ATCC	DMEM/F-12 (11320033, Thermofisher Scientific)	10% FBS (Bovogen Biological); Penicilin (100 IU/ml)/Streptomycin (100 µg/ml) (P4333, Sigma-Aldrich)	15 mM HEPES & 1.2 g/l sodium bicarbonate (included in the culture medium)
	M059K	CVCL_0401				
	U-251 MG	CVCL_0021				
	A-172	CVCL_0131				
astrocytoma	CCF-STTG1	CVCL_1118				
human neonatal dermal fibroblast	NDF-2	-	Lonza Bioscience (#CC-2509), Kind gift of Dr. Bárta (Masaryk University)	DMEM High Glucose (PM-D1114, Biosera)	10% FBS (FB-1101); 2 mM L-Glutamine (XC-T1715); 1× MEM Non-Essential Amino Acids (XC-E1154); Penicilin (100 IU/ml) /Streptomycin (100 µg/ml) (X-A4122) all Biosera	15 mM HEPES (H0887) & 1.2 g/l sodium bicarbonate (S8761), both Sigma-Aldrich
	NDF-3					
embryonal carcinoma	NTERA-2 clone D1	CVCL_3407	ECACC		10% FBS (FB-1101); 2 mM L-Glutamine (XC-T1715); Penicilin (100 IU/ml) /Streptomycin (100 µg/ml) (X-A4122) all Biosera	3.7 g/l sodium bicarbonate (included in the culture medium

Providers: ATCC, The American Type Culture Collection (Manassas, VA, USA); Biosera (Nuaille, France), Bovogen Biological (Keilor, Victoria, Australia); ECACC, The European Collection of Authenticated Cell Cultures, UK Health Security Agency); Gibco (Carlsbad, CA, USA); Lonza Bioscience (Basel, Switzerland); Sigma-Aldrich (St. Louis, MO, USA). FBS, fetal bovine serum; HEPES, N-(2-Hydroxyethyl)piperazine-N′-(2-ethanesulfonic acid), CAS #7365-45-9.

Supplementary Table 3. Drugs used in the study

Drug	Manufacturer	Catalog number	Dissolvent
Ampicillin	Sigma-Aldrich	A9393	H_2O
ABT-737	Abcam	ab141336	DMSO
Carboplatin	EMD Millipore	216100	PBS
Chloramphenicol	Serva	1678502	H_2O
Cisplatin	Sigma-Aldrich	232120	PBS
Crizotinib	Sigma-Aldrich	PZ0191	DMSO
Doxorubicine	Cell Signaling Technology	5927	DMSO
Doxycycline hyclate	Sigma-Aldrich	D9891	H_2O
(R)-(+)-Etomoxir sodium salt	Tocris	4539	H_2O
Etoposide	EMD Millipore	341205	DMSO
ISRIB	Sigma-Aldrich	SML0843	DMSO
Linezolid	Sigma-Aldrich	PZ0014	DMSO
Lorlatinib	Tocris	5640	DMSO
Mdivi 1	Tocris	3982	DMSO
MG-132	Tocris	1748	DMSO
Phenformin hydrochloride	Sigma-Aldrich	P7045	H_2O
SB203580	Tocris	1202	DMSO
Thapsigargin	Tocris	1138	DMSO
Tigecycline hydrate	Sigma-Aldrich	PZ0021	DMSO
Trametinib	APEXBIO	GEN1590424	DMSO
Valspodar	Sigma-Aldrich	SML0572	DMSO
Vincristine	Tocris	1257	DMSO

Providers: Abcam (Cambridge, UK), APEXBIO (Houston, TX, USA), Cell Signaling Technology (Danvers, MA, USA), EMD Millipore (Billerica, MA, USA), Sigma-Aldrich (St. Louis, MO, USA), Serva (Heidelberg, Germany), Tocris (Bristol, UK).

Supplementary Table 4. Antibodies used for immunodetection

Primary Antibodies (Antigen)	Manufacturer	Catalog Number	Blocking agent	Method	Dilution
ABCB1 (P-gp)	Sigma-Aldrich	P7965	NFM	WB	1:1 000
ABCC1 (MRP1)	CST	14685	NFM	WB	1:1 000
ABCG2	Abcam	ab130244	NFM	WB	1:1 000
AMPKα	CST	5832	NFM	WB	1:1 000
phospho-AMPKα (Thr172/Thr183)	Abcam	ab133448	NFM	WB	1:1 000
ATF4	CST	11815	NFM	WB	1:1 000
ATP5A1	CST	18023	NFM	WB	1:2 000
BCL-2	CST	15071	NFM	WB	1:2 000
C-MYC	CST	5605	NFM	WB	1:2 000
phospho-C-MYC (Thr58)	CST	46650	BSA	WB	1:1 000
phospho-C-MYC (Ser62)	CST	13748	NFM	WB	1:1 000
CHOP	CST	2895	NFM	WB	1:1 000
CD133	Abcam	ab19898	NFM	WB	1:1 000
Cleaved Caspase 3	CST	9661	BSA	IF	1:200
Cleaved Caspase 3	CST	9664	NFM	WB	1:1 000
DRP1	CST	8570	NFM	WB	1:1 000
phospho-DRP1 (Ser616)	CST	4494	NFM	WB	1:1 000
phospho-DRP1 (Ser637)	CST	4867	BSA	WB	1:1 000
eIF2α	CST	5324	NFM	WB	1:3 000
phospho-eIF2α (Ser51)	CST	3398	NFM	WB	1:1 000
GAPDH	CST	2118	NFM	WB	1:10 000
GAPDH	SCBT	sc-365062	NFM	WB	1:10 000
HIF1α	CST	36169	NFM	WB	1:1 000
KLF4	CST	4038	NFM	WB	1:1 000
MCL-1	CST	94296	NFM	WB	1:2 000
MFF	CST	84580	BSA	WB	1:2 000
MFN1	CST	14739	BSA	WB	1:2 000
MT-CO1	Abcam	ab14705	NFM	WB	1:1 000
N-MYC	CST	84406	NFM	WB	1:2 000
NANOG	CST	4893	NFM	WB	1:1 000
Nestin	Abcam	ab18102	NFM	WB	1:1 000
OMA1	CST	95473	NFM	WB	1:1 000
OPA1	CST	80471	BSA	WB	1:2 000
p38αβγ MAPK	CST	9212	NFM	WB	1:2 000
phospho-p38 MAPK (Thr180/Tyr182)	CST	4511	BSA	WB	1:1 000
PERK	CST	3192	NFM	WB	1:1 000
PGC-1α	Abcam	ab54481	NFM	WB	1:1 000
OCT4	CST	2750	NFM	WB	1:1 000
SOX2	CST	3579	NFM	WB	1:1 000
TOMM20	SCBT	sc-17764	BSA	IF	1:200
TOMM20	CST	42406	NFM	WB	1:3 000
YME1L1	Proteintech	11510-1-AP	NFM	WB	1:1 000
α-tubulin	Abcam	ab7291	NFM	WB	1:10 000
β-actin	Sigma	A1978	NFM	WB	1:10 000
β-tubulin	CST	86298	NFM	WB	1:5 000
**Secondary Antibodies**	**Manufacturer**	**Catalog Number**	**Blocking agent**	**Method**	**Dilution**
Anti-mouse IgG, HRP-linked	CST	7076	NFM	WB	1:5 000; 1:10 0000
Anti-rabbit IgG, HRP-linked	CST	7074	NFM	WB	1:5 000; 1:10 0000
Anti-mouse IgG Alexa Fluor ® 488	Invitrogen*	A21202	BSA	IF	1:200
Anti-mouse IgG Alexa Fluor ® 568	Invitrogen	A10042	BSA	IF	1:200
**Isotype Control Antibodies**	**Manufacturer**	**Catalog Number**	**Blocking agent**	**Method**	**Dilution**
F (ab')2 IgG Rabbit Isotype Control	Sigma-Aldrich	ab37416-5	BSA	IF	1:20 000
IgG2a Mouse Isotype Control	Invitrogen	MG2a00	BSA	IF	1:100

Providers: Abcam (Cambridge, UK), CST – Cell Signaling Technology, Inc.,(Danvers, MA, USA), Invitrogen, (Carlsbad, CA, USA), Proteintech Group (Rosemont, IL, USA), SCBT – Santa Cruz Biotechnology, Inc., (Dallas, TX, USA), Sigma-Aldrich (St. Louis, MO, USA). BSA, bovine serum albumin; IF, immunofluorescence staining; NFM, dry non-fat milk; WB, western blotting.

Supplementary Table 5. The sequences of primers used for RT-qPCR

List of primers		
**Gene**	**Forward (5'→3')**	**Reverse (3'→5')**
HIF1A	CAAGAACCTACTGCTAATGC	TTATGTATGTGGGTAGGAGATG
HSP90AB1	CGCATGAAGGAGACACAGAA	TCCCATCAAATTCCTTGAGC
MCL1	GGACAAAACGGGACTGGCT	ATGCCACCTTCTAGGTCCTCT
MYC	TCTCTCCGTCCTCGGATTCT	GCCTCTTTTCCACAGAAACAACA
