# Supplementary material for: Mitoribosomal synthetic lethality overcomes multidrug resistance in MYC-driven neuroblastoma
Source: Cell Death Dis. 2023 Nov 16;14(11):747. doi: 10.1038/s41419-023-06278-x (PMC10654511; doi:10.1038/s41419-023-06278-x)

Figure 1d

*Uncropped images of experiments displayed in **Figure 1d** are followed by other biological replicates. Red box indicates lines related to the figure. Sample order same as displayed in the figure if not indicated differently.*

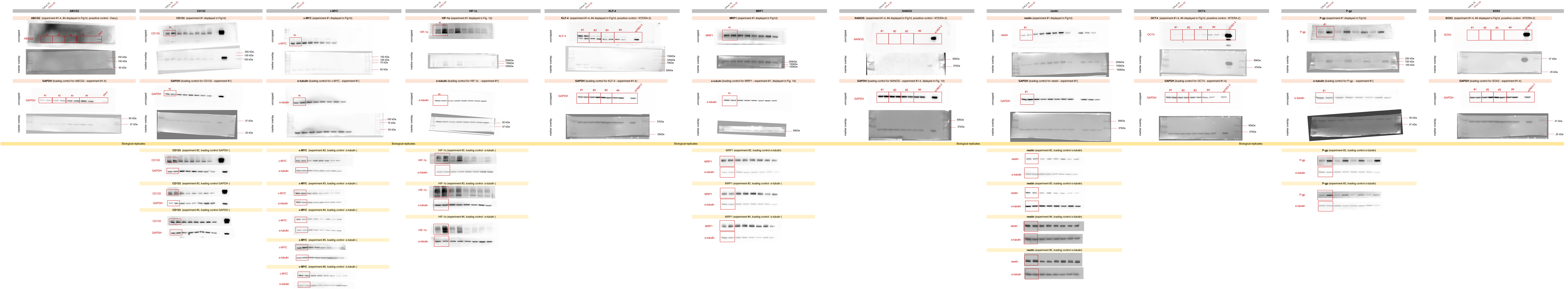

Figure 2d

Uncropped images of experiments displayed in ***Figure 2d*** *are followed by other biological replicates. All lines are related to the figure, otherwise red box indicates lines related to the figure. Sample order same as displayed in the figure if not indicated differently.*

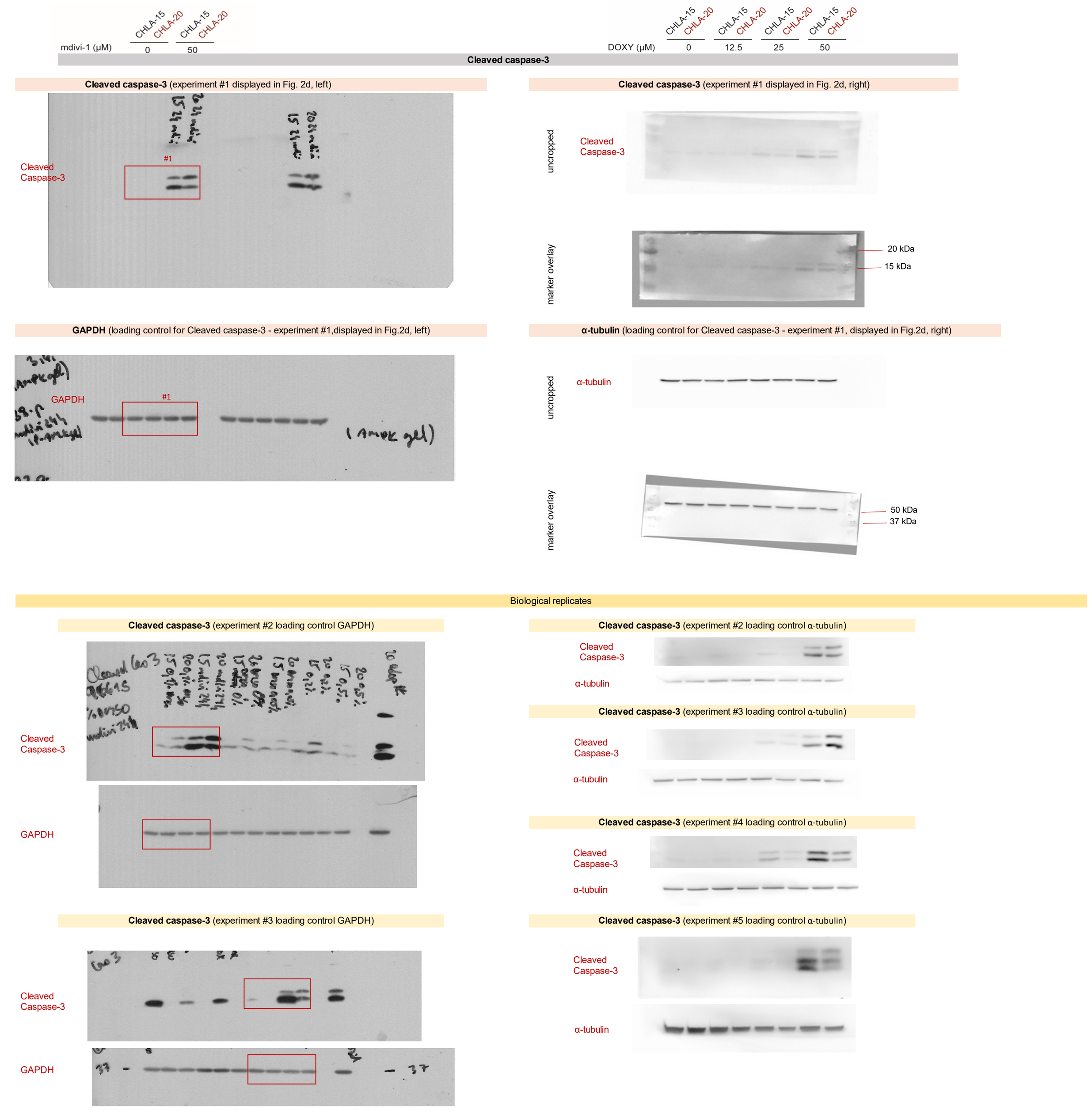

Figure 3a

*Uncropped images of experiments displayed in **Figure 3a** are followed by other biological replicates. Sample order same as displayed in the figure if not indicated differently.*

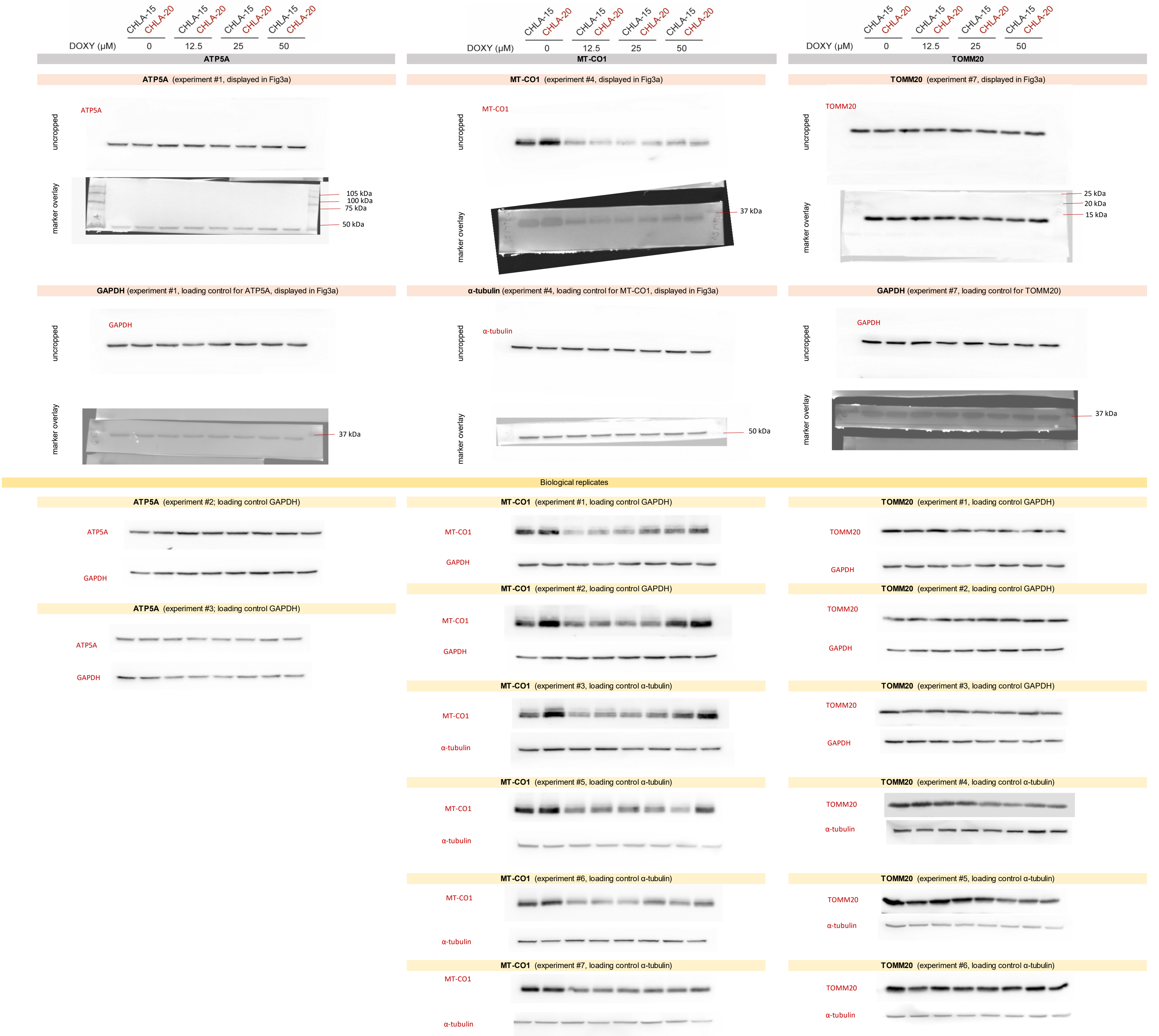

Figure 3i

*Uncropped images of experiments displayed in **Figure 3i** are followed by other biological replicates. All lines are related to the figure, otherwise red box indicates lines related to the figure. Sample order same as displayed in the figure if not indicated differently.*

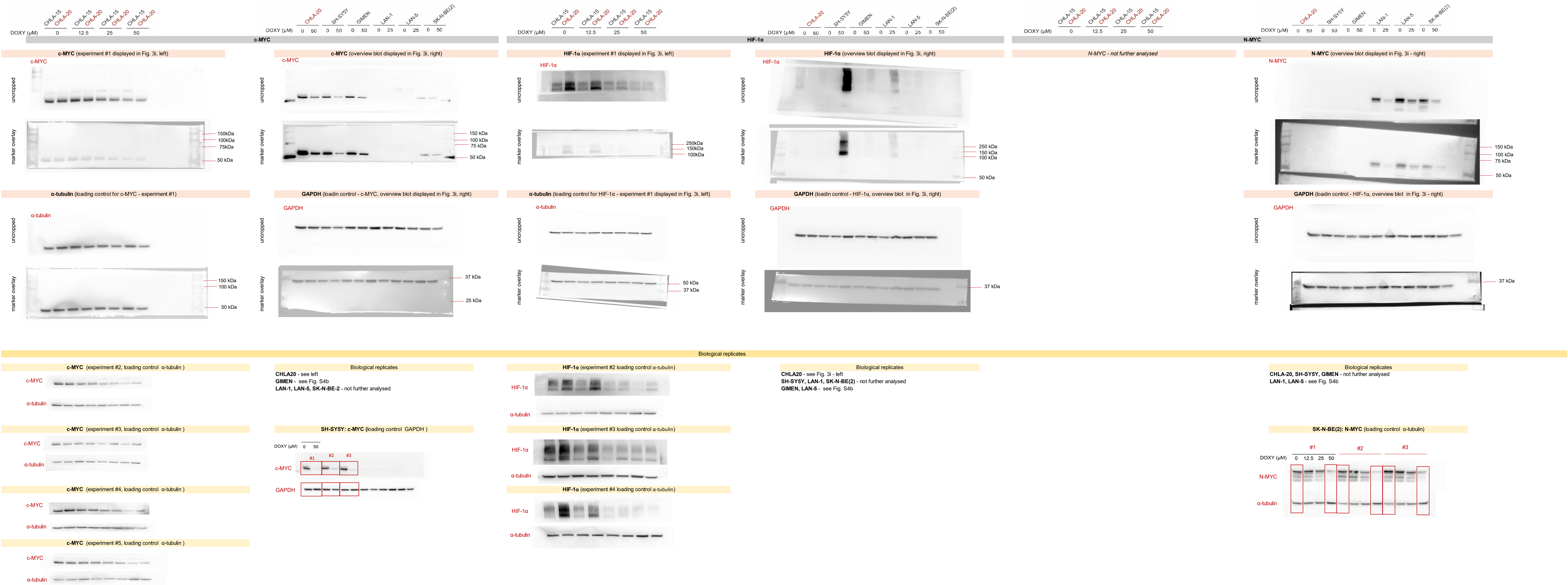

Figure 4d

*Uncropped images of experiments displayed in **Figure 4d** are followed by other biological replicates. All lines are related to the figure, otherwise red box indicates lines related to the figure. Sample order same as displayed in the figure if not indicated differently.*

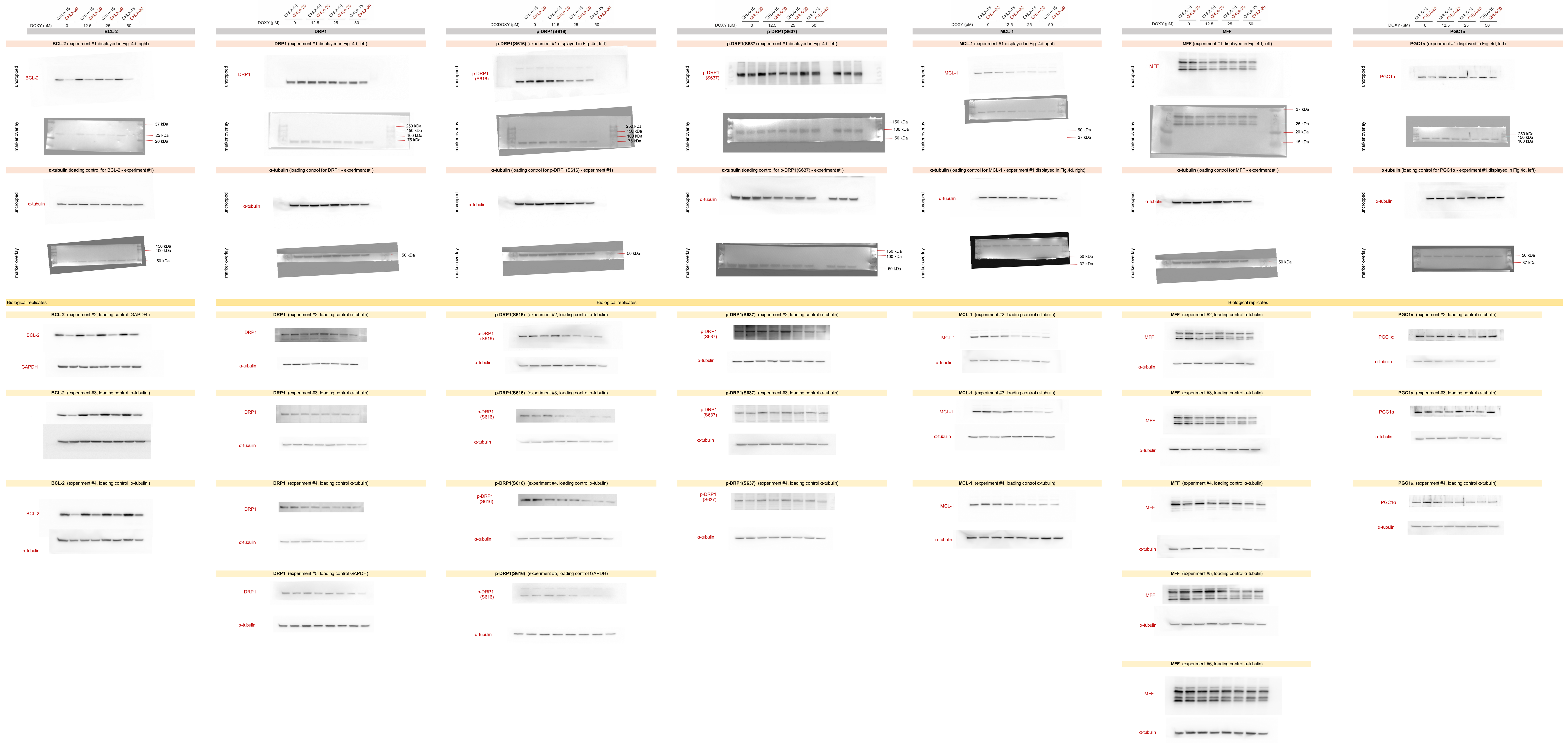

Figure 5a

*Uncropped images of experiments displayed in **Figure 5a** are followed by other biological replicates. All lines are related to the figure, otherwise red box indicates lines related to the figure. Sample order same as displayed in the figure if not indicated differently.*

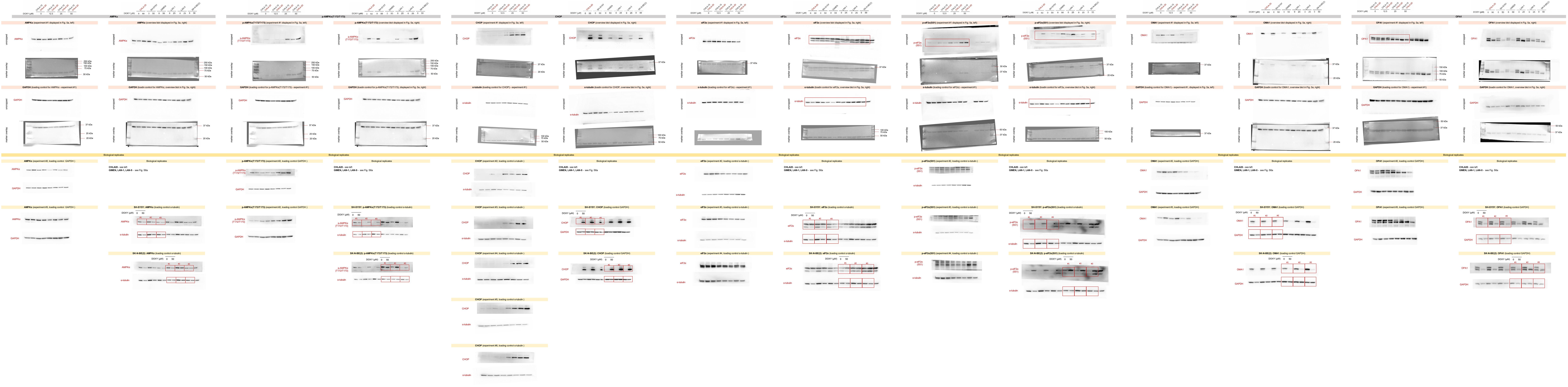

Figure 5a - continued

*Uncropped images of experiments displayed in **Figure 5c** are followed by other biological replicates. All lines are related to the figure, otherwise red box indicates lines related to the figure. Sample order same as displayed in the figure if not indicated differently.*

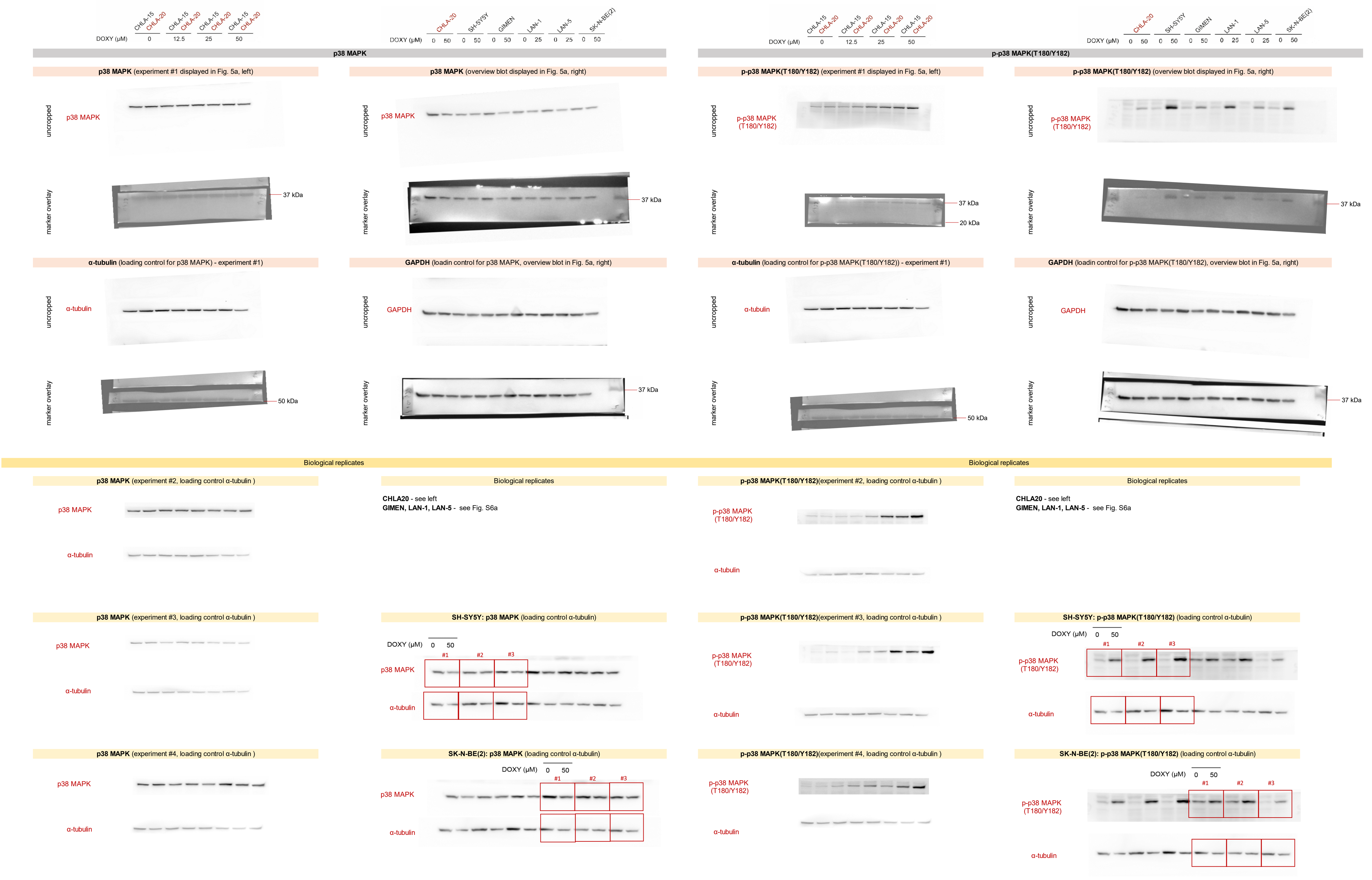

Figure 5c

Uncropped images of experiments displayed in ***Figure 5c*** *are followed by other biological replicates. All lines are related to the figure, otherwise red box indicates lines related to the figure. Sample order same as displayed in the figure if not indicated differently.*

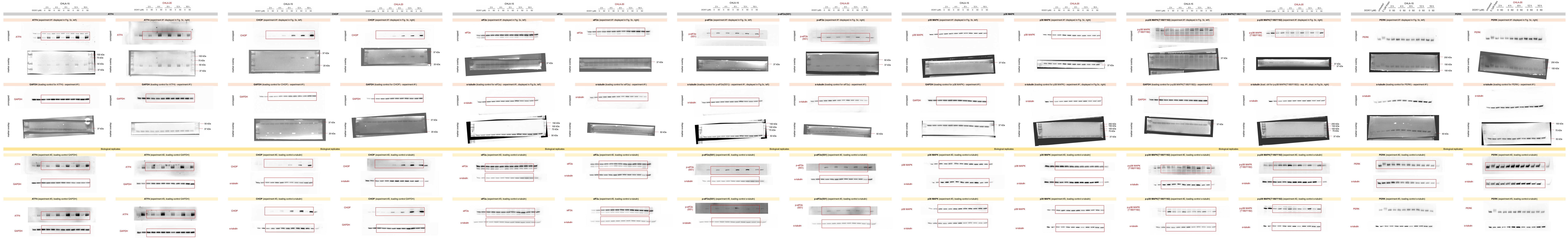

Figure 5c - continued

*Uncropped images of experiments displayed in **Figure 5c** are followed by other biological replicates. All lines are related to the figure, otherwise red box indicates lines related to the figure. Sample order same as displayed in the figure if not indicated differently.*

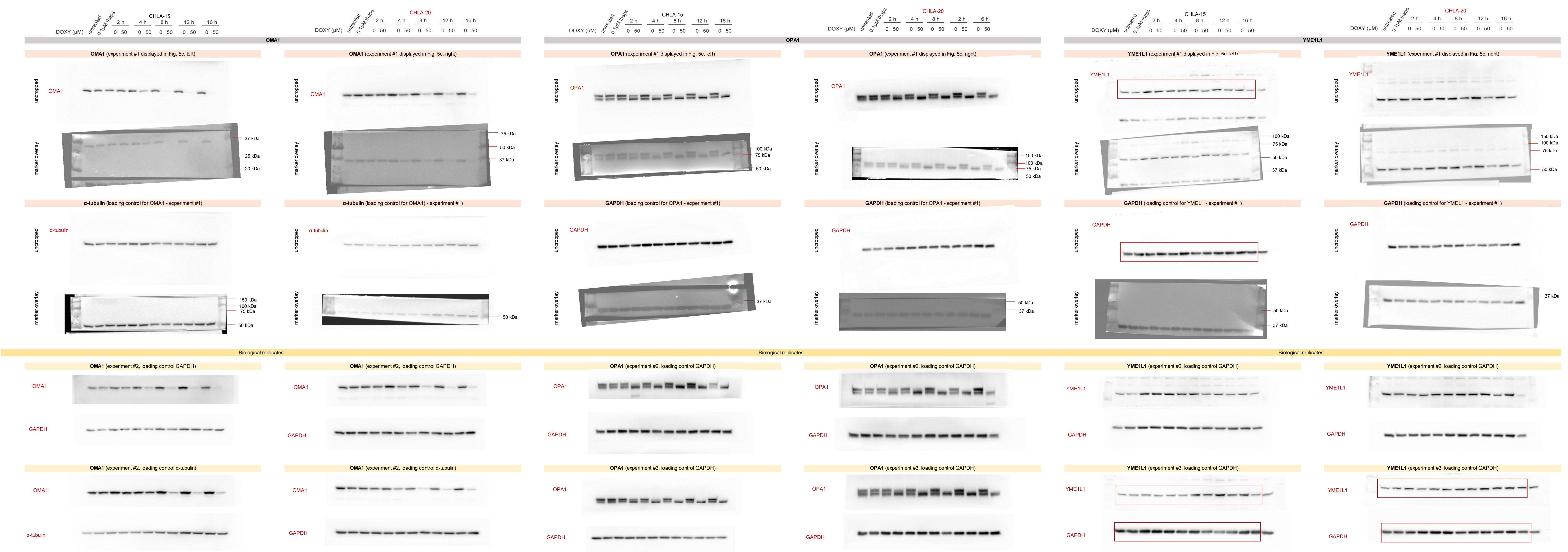

Figure 6a

Uncropped images of experiments displayed in ***Figure 6a*** *are followed by other biological replicates. All lines are related to the figure, otherwise red box indicates lines related to the figure. Sample order same as displayed in the figure if not indicated differently.*

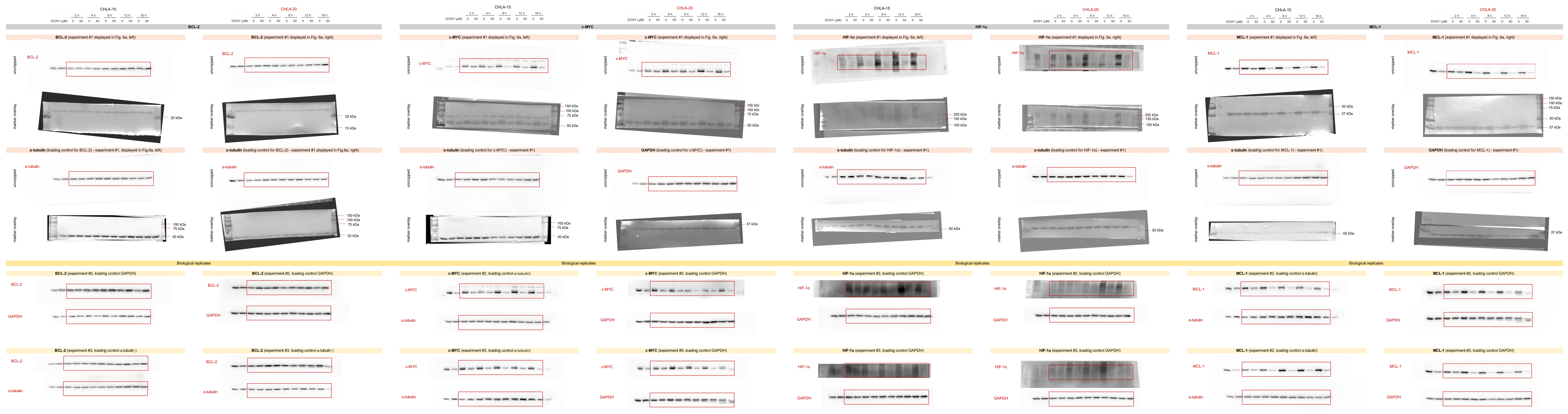

Figure 6d

Uncropped images of experiments displayed in ***Figure 6d*** *are followed by other biological replicates. Sample order same as displayed in the figure if not indicated differently.*

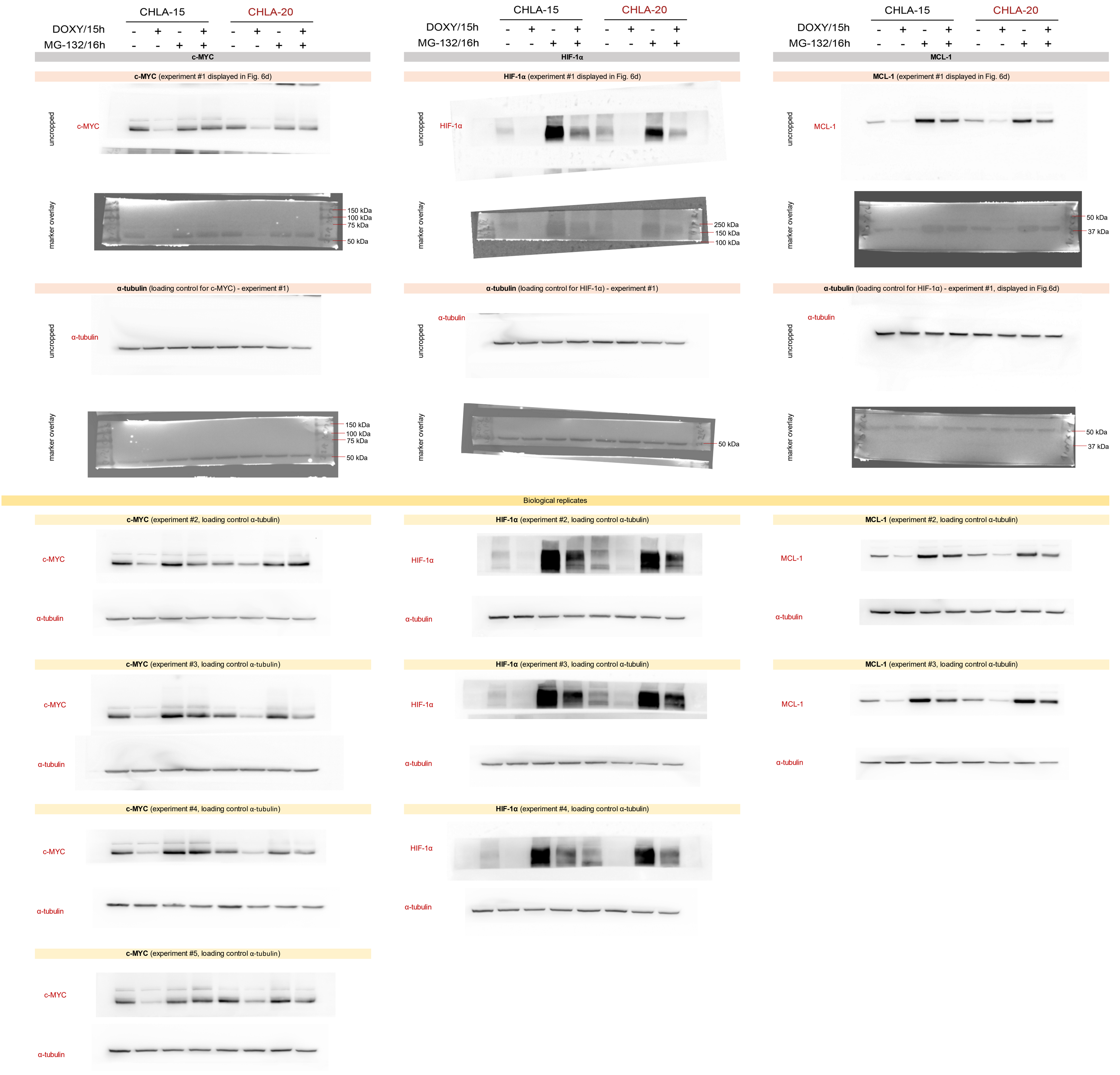

Figure 6g

Uncropped images of experiments displayed in ***Figure 6g*** *are followed by other biological replicates. Sample order same as displayed in the figure if not indicated differently.*

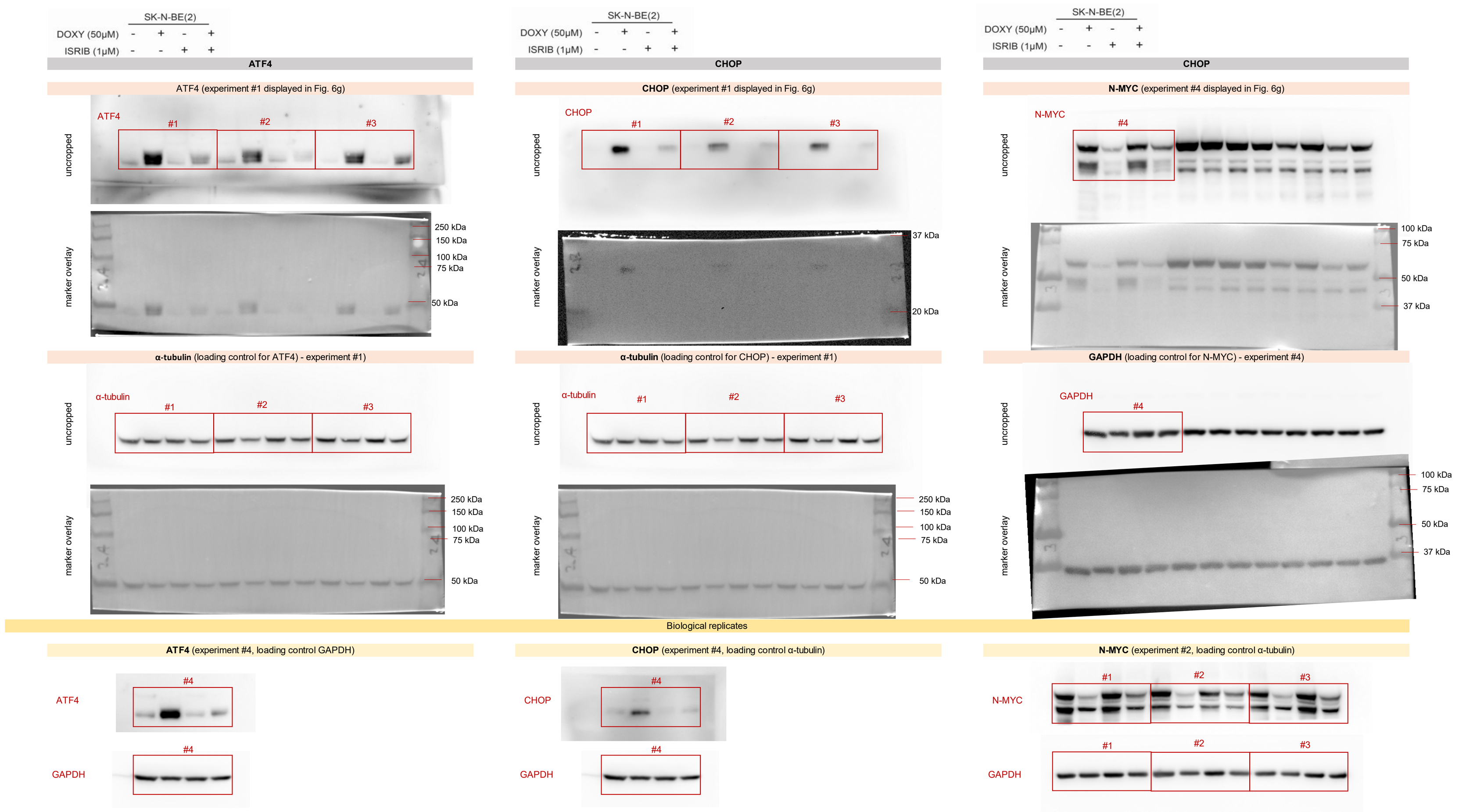

Figure 6h

*Uncropped images of experiments displayed in **Figure 6h** are followed by other biological replicates. Sample order same as displayed in the figure if not indicated differently.*

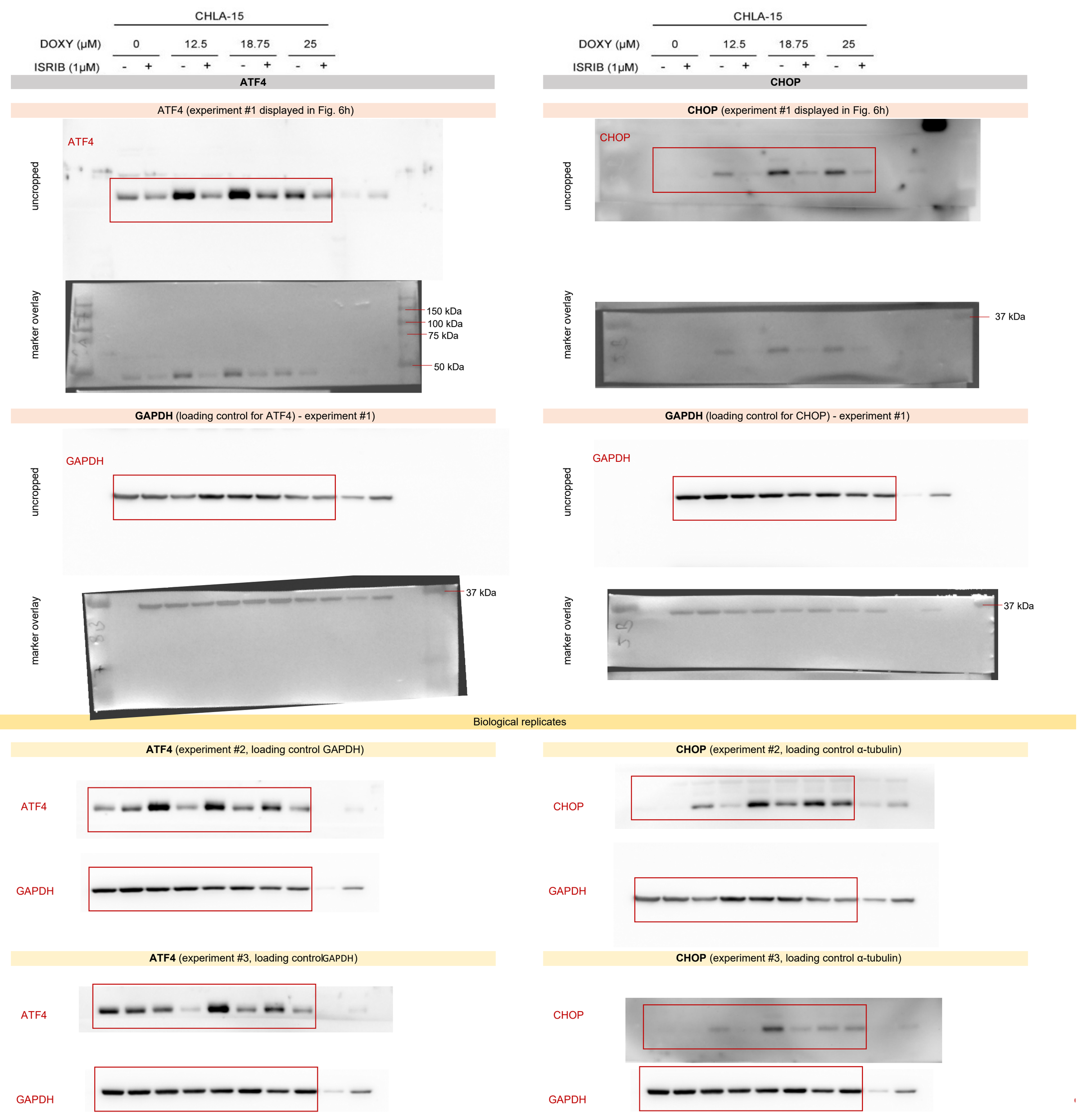

Figure 7c

Uncropped images of experiments displayed in ***Figure 7c*** *are followed by other biological replicates. Red box indicates lines related to the figure. Sample order same as displayed in the figure if not indicated differently.*

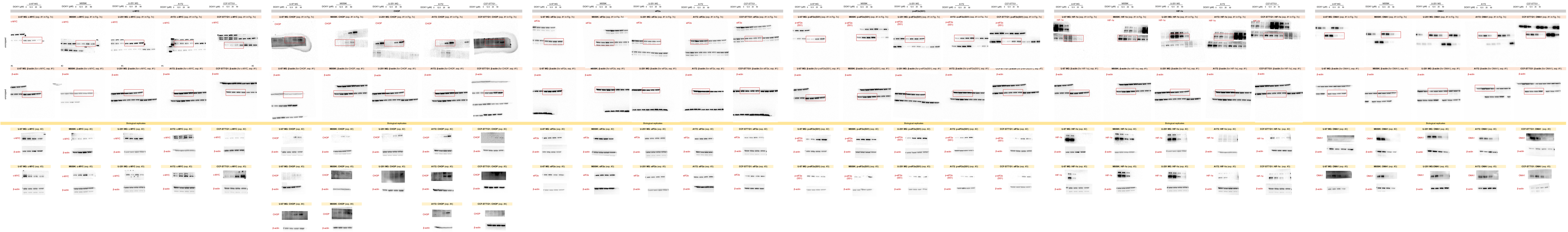

Figure 7d

*Uncropped images of experiments displayed in **Figure 7d** are followed by other biological replicates. Red box indicates lines related to the figure. Sample order same as displayed in the figure if not indicated differently.*

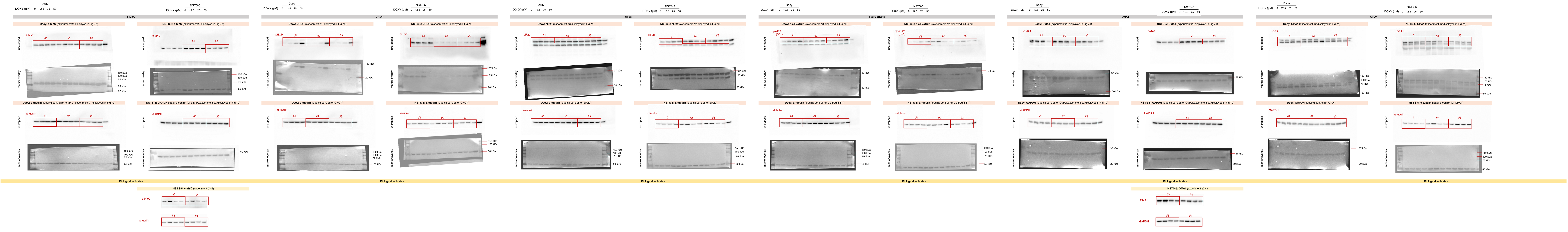

Figure 7e

*Uncropped images of experiments displayed in **Figure 7e** are followed by other biological replicates. Red box indicates lines related to the figure. Sample order same as displayed in the figure if not indicated differently.*

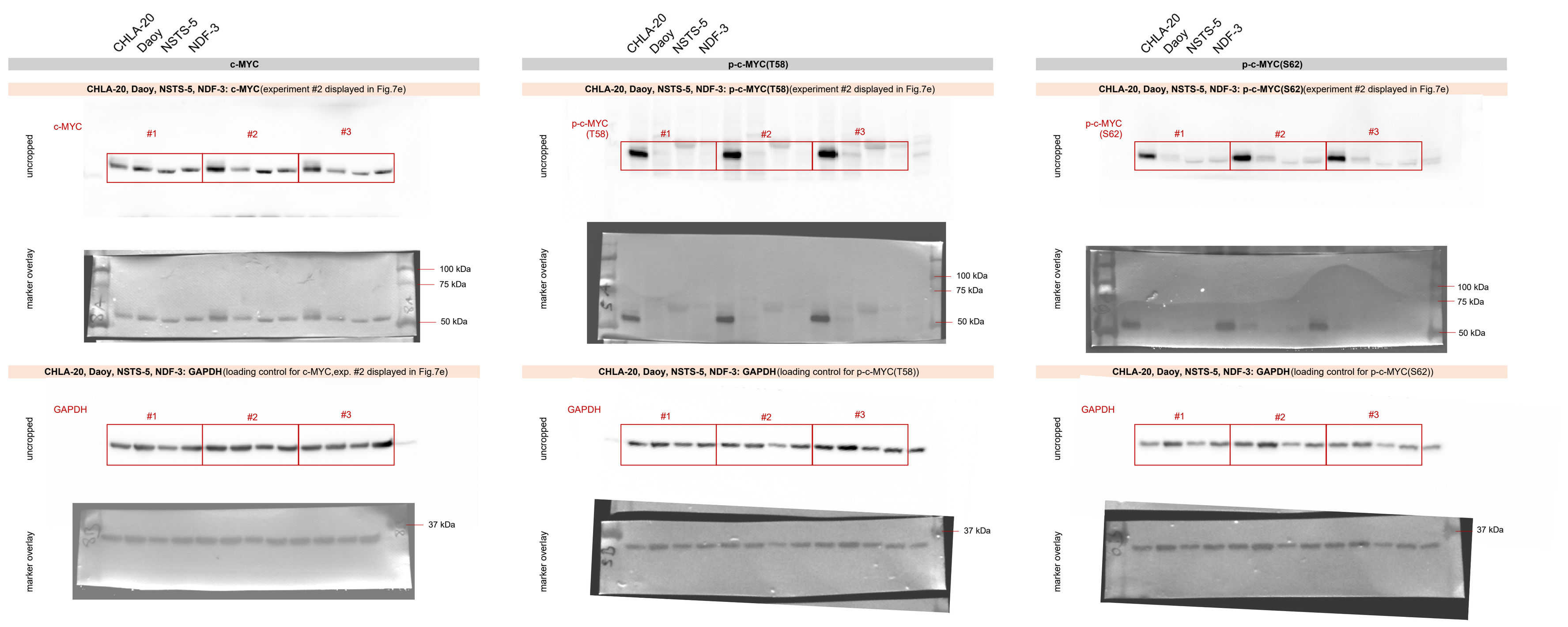

Figure 7f

*Uncropped images of experiments displayed in **Figure 7f** are followed by other biological replicates. Red box indicates lines related to the figure. Sample order same as displayed in the figure if not indicated differently.*

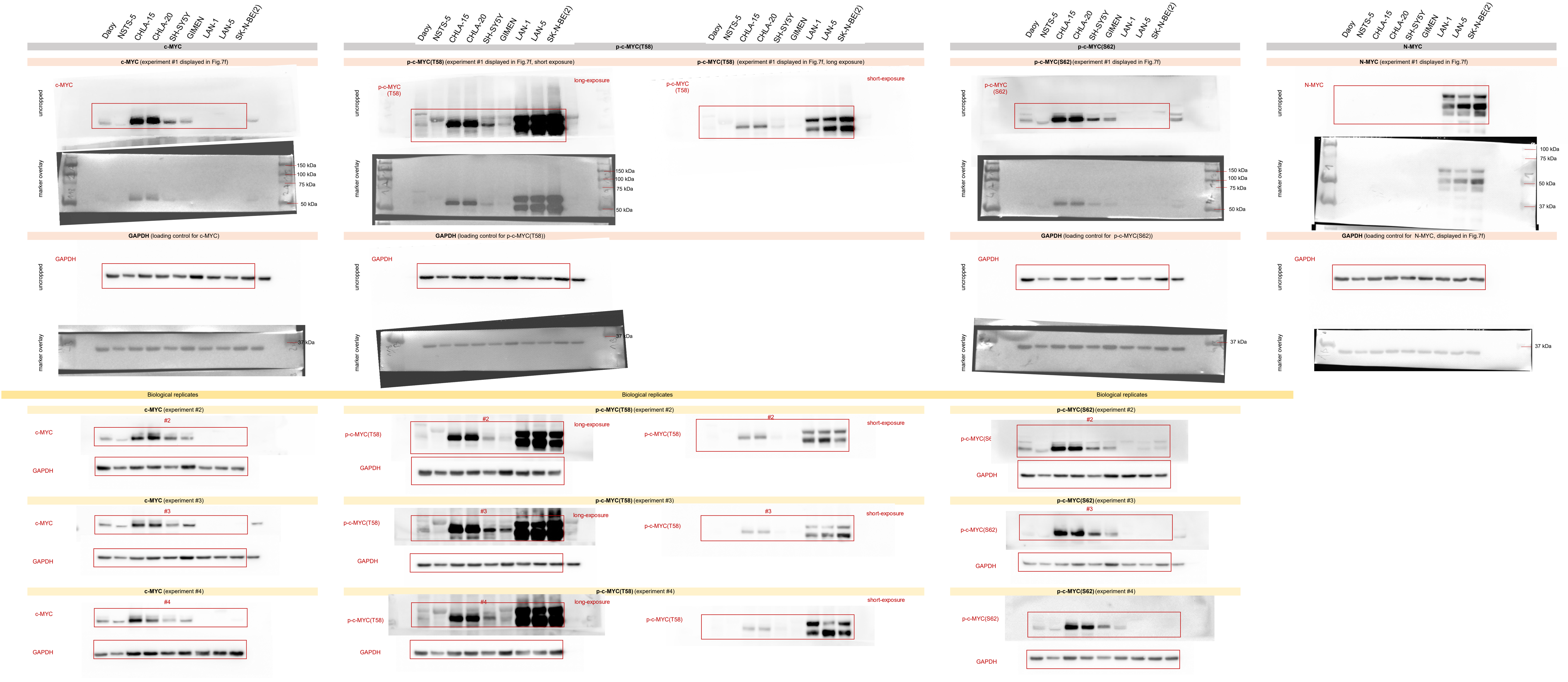

Figure 7h

Uncropped images of experiments displayed in ***Figure 7h*** *are followed by other biological replicates. Red box indicates lines related to t* *Uncropped images of experiments displayed in* ***Figure 7h*** *are followed by other biological replicates. Red box indicates lines related to the figure. Sample order same as displayed in the figure if not indicated differently.*

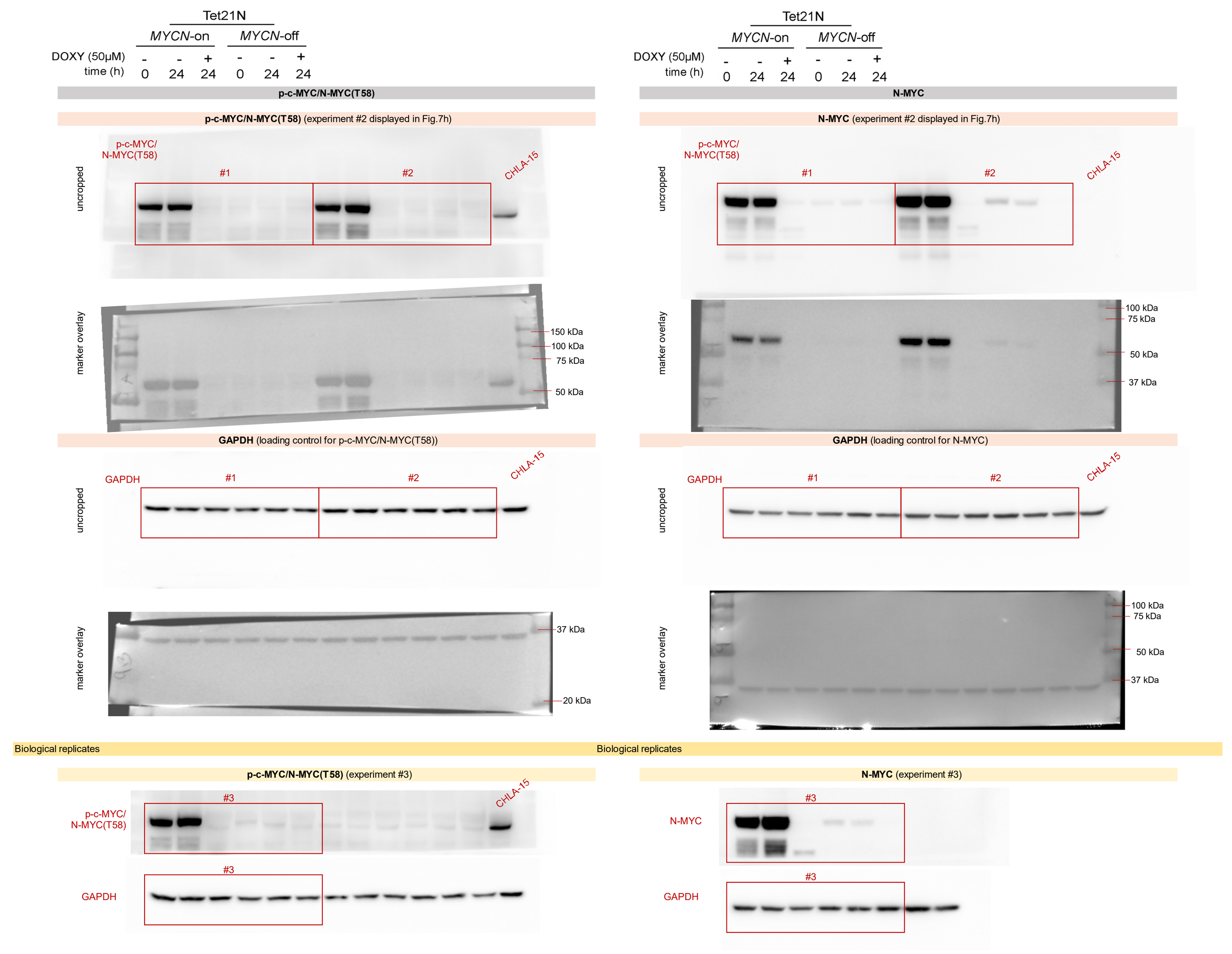

Figure S4b

*Uncropped images of experiments displayed in **Figure S4b** are followed by other biological replicates. Red box indicates lines related to the figure. Sample order same as displayed in the figure if not indicated differently.*

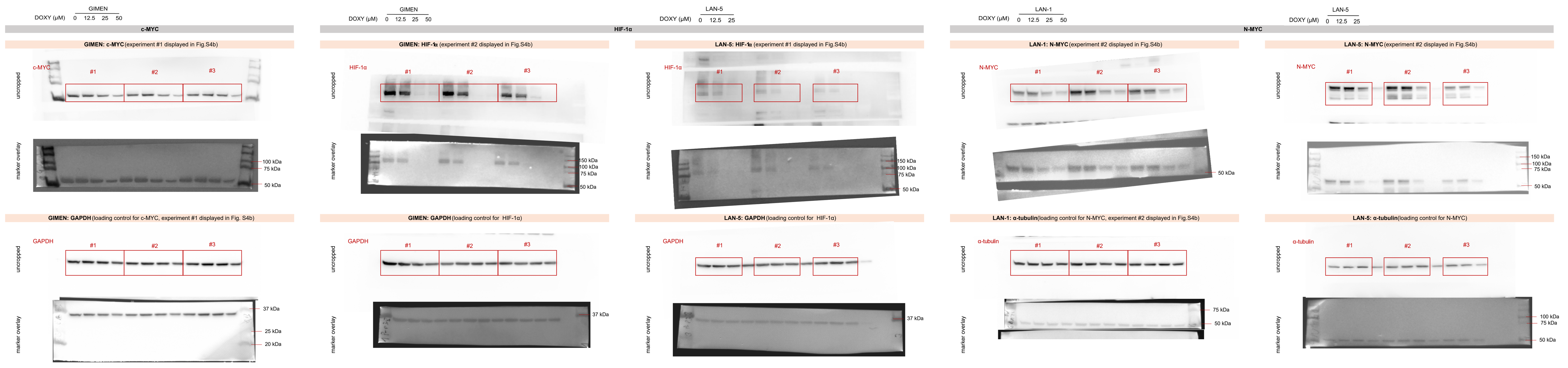

Figure S5a

Uncropped images of experiments displayed in ***Figure S5a*** *are followed by other biological replicates. Red box indicates lines related to the figure. Sample order same as displayed in the figure if not indicated differently.*

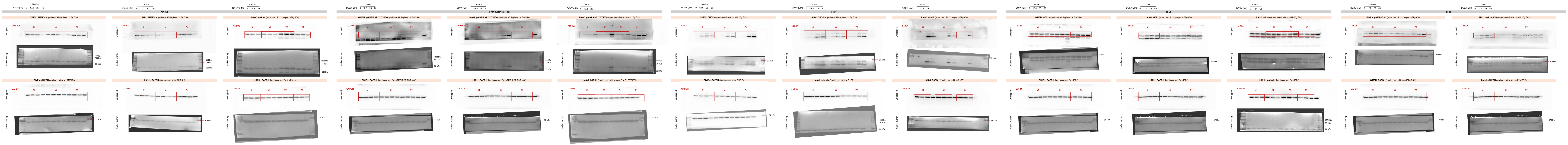

Figure S5a - continued

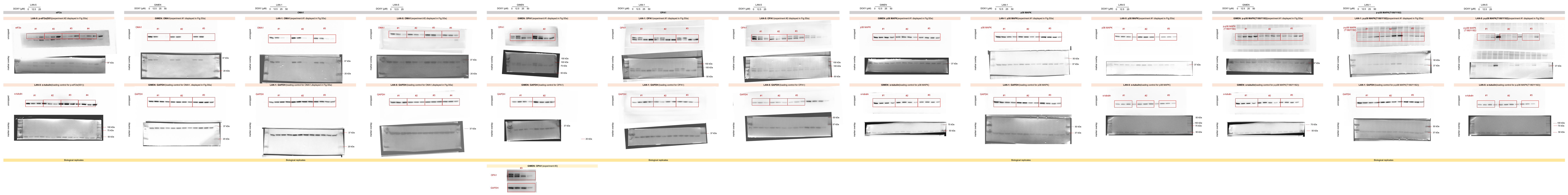

Figure S5c

*Uncropped images of experiments displayed in **Figure S5c** are followed by other biological replicates. Red box indicates lines related to the figure. Sample order same as displayed in the figure if not indicated differently.*

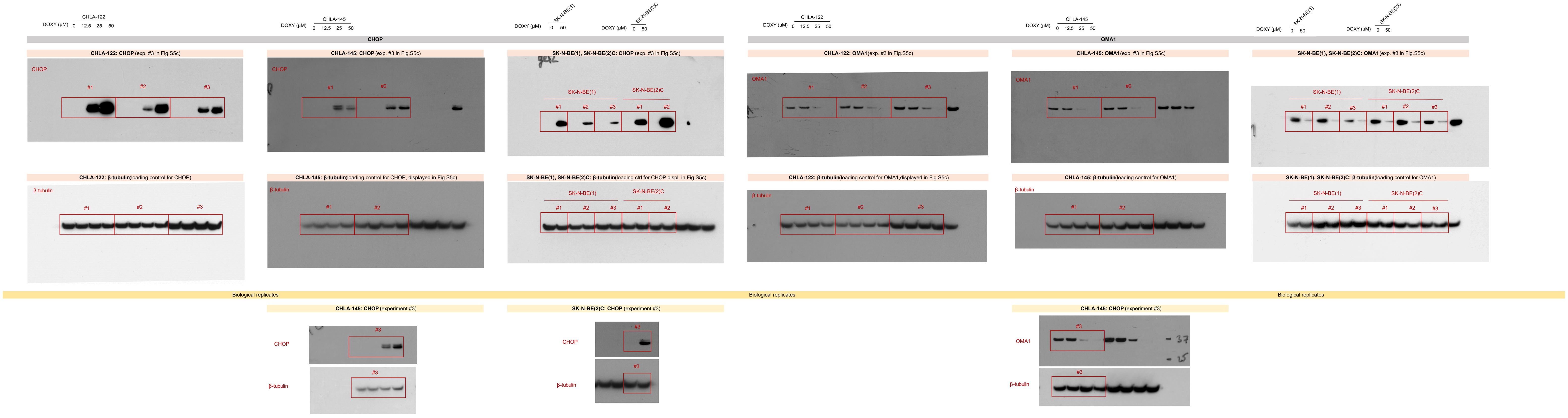

Figure S6a

*Uncropped images of experiments displayed in **Figure S6a** are followed by other biological replicates. Red box indicates lines related to the figure. Sample order same as displayed in the figure if not indicated differently.*

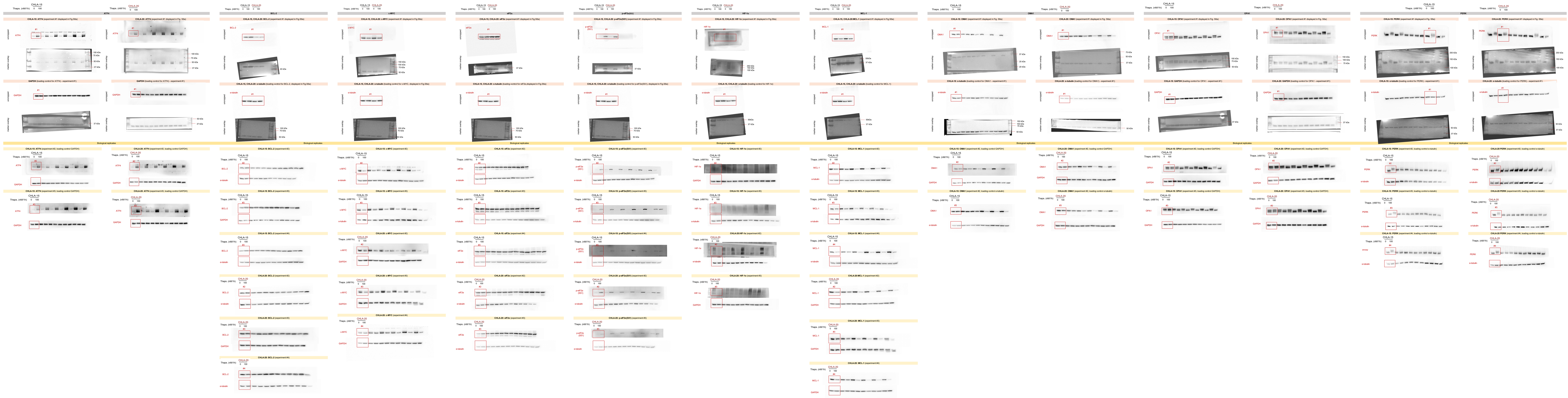

Figure S6b

*Uncropped images of experiments displayed in **Figure S6b** are followed by other biological replicates. Red box indicates lines related to the figure. Sample order same as displayed in the figure if not indicated differently.*

DOXY (µM)	0		12.5		25		50	
	CHLA-15	CHLA-20	CHLA-15	CHLA-20	CHLA-15	CHLA-20	CHLA-15	CHLA-20

YME1L1

CHLA-15, CHLA-20: YME1L1 (experiment #1 displayed in Fig. S6b)

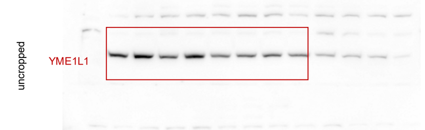

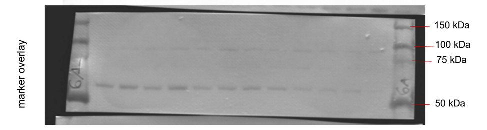

GAPDH (loading control for YME1L1- experiment #1, displayed in Fig.S6b)

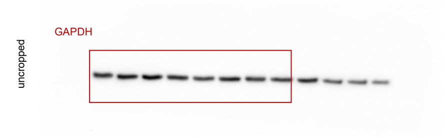

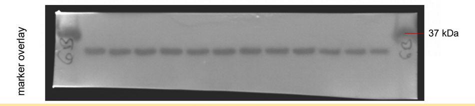

Biological replicates

CHLA-15, CHLA-20: YME1L1 (experiment #2, loading control GAPDH)

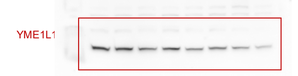

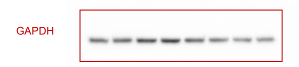

CHLA-15, CHLA-20: YME1L1 (experiment #3, loading control GAPDH)

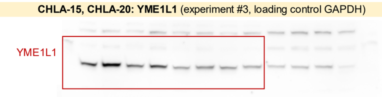

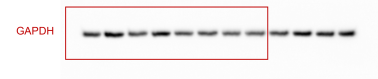

Figure S6d

*Uncropped images of experiments displayed in **Figure S6d** are followed by other biological replicates. Red box indicates lines related to the figure. Sample order same as displayed in the figure if not indicated differently.*

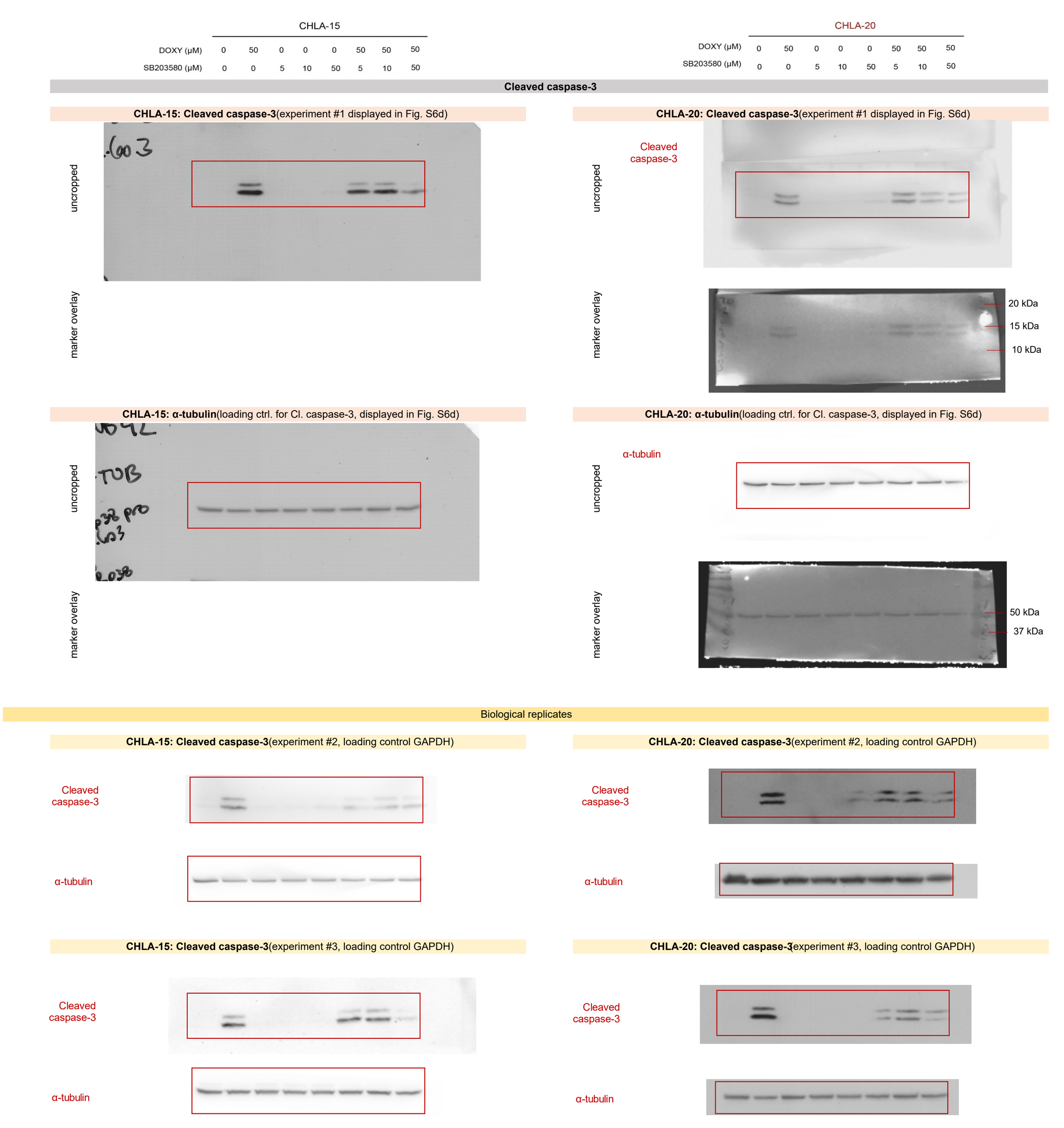

Figure S7a

Uncropped images of experiments displayed in ***Figure S7a*** *are followed by other biological replicates. Sample order same as displayed in the figure if not indicated differently.*

	CHLA-15				CHLA-20			
DOXY/15h	-	+	-	+	-	+	-	+
MG-132/16h	-	-	+	+	-	-	+	+

p-c-MYC(T58)

p-c-MYC(T58) (experiment #1 displayed in Fig.S7a)

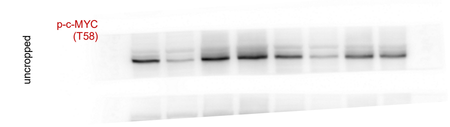

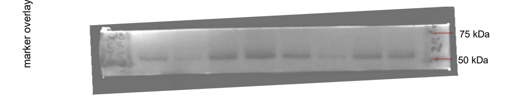

α-tubulin (loading control for p-c-MYC(T58)) - experiment #1)

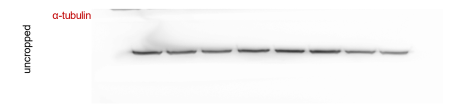

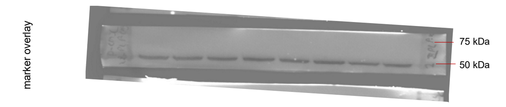

Biological replicates

p-c-MYC(T58) (experiment #2, loading control α-tubulin)

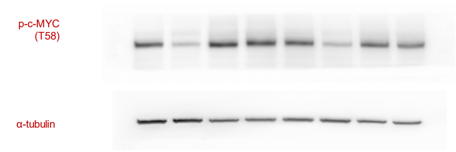

p-c-MYC(T58) (experiment #3, loading control α-tubulin)

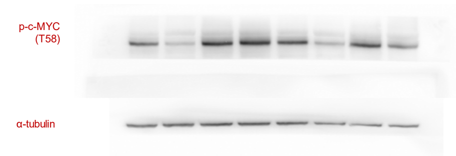

p-c-MYC(T58) (experiment #4, loading control α-tubulin)

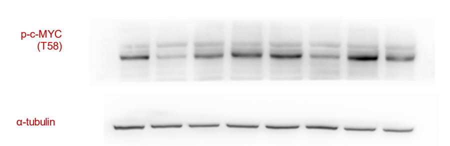

p-c-MYC(T58) (experiment #5, loading control α-tubulin)

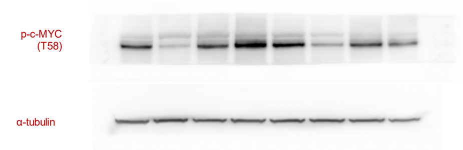

Figure S9a

Uncropped images of experiments displayed in ***Figure S9a*** *are followed by other biological replicates. Red box indicates lines related to the figure. Sample order same as displayed in the figure if not indicated differently.*

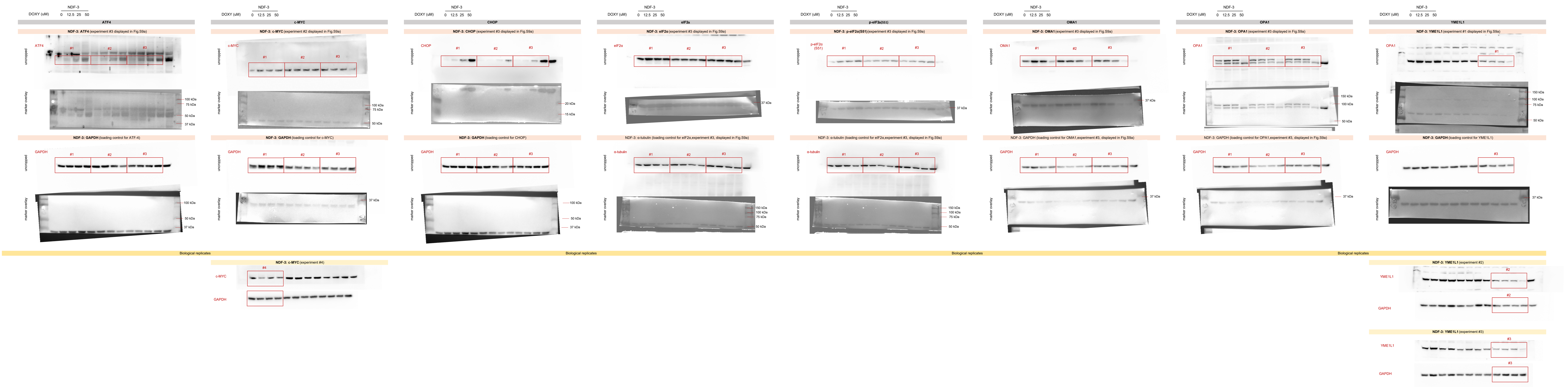

Figure S11b

*Uncropped images of experiments displayed in **Figure S11b** are followed by other biological replicates. Red box indicates lines related to the figure. Sample order same as displayed in the figure if not indicated differently.*

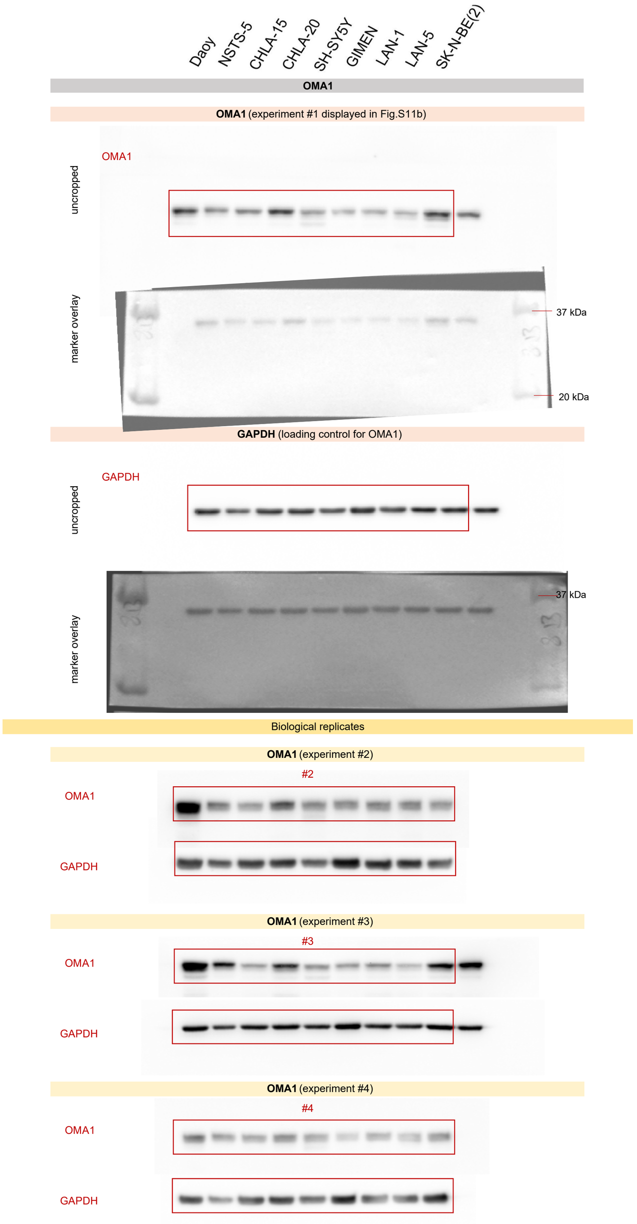

Figure S13a

*Uncropped images of experiments displayed in **Figure S13a** are followed by other biological replicates. Red box indicates lines related to the figure. Sample order same as displayed in the figure if not indicated differently.*

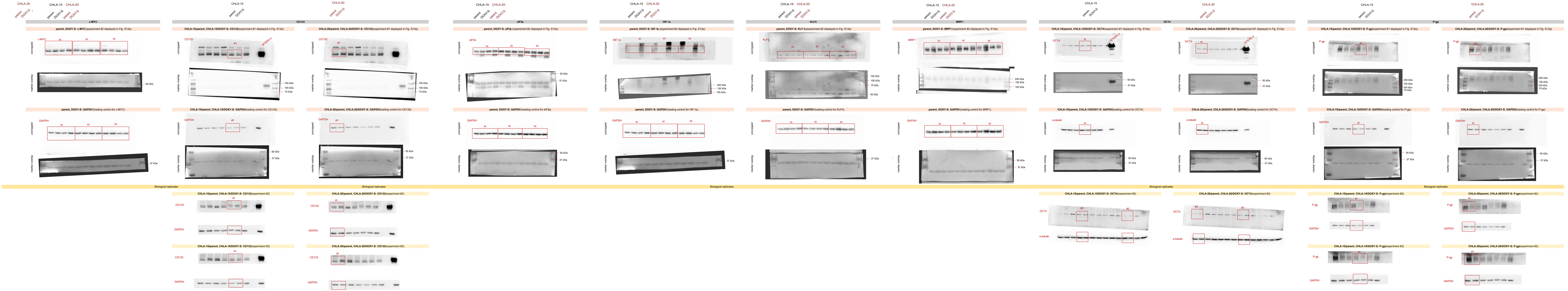

Figure S13b

Uncropped images of experiments displayed in ***Figure S13b*** *are followed by other biological replicates. Red box indicates lines related to the figure. Sample order same as displayed in the figure if not indicated differently.*

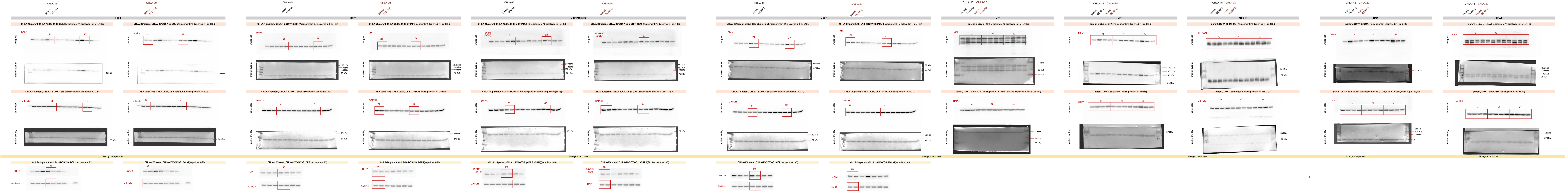

Figure S13b - continued

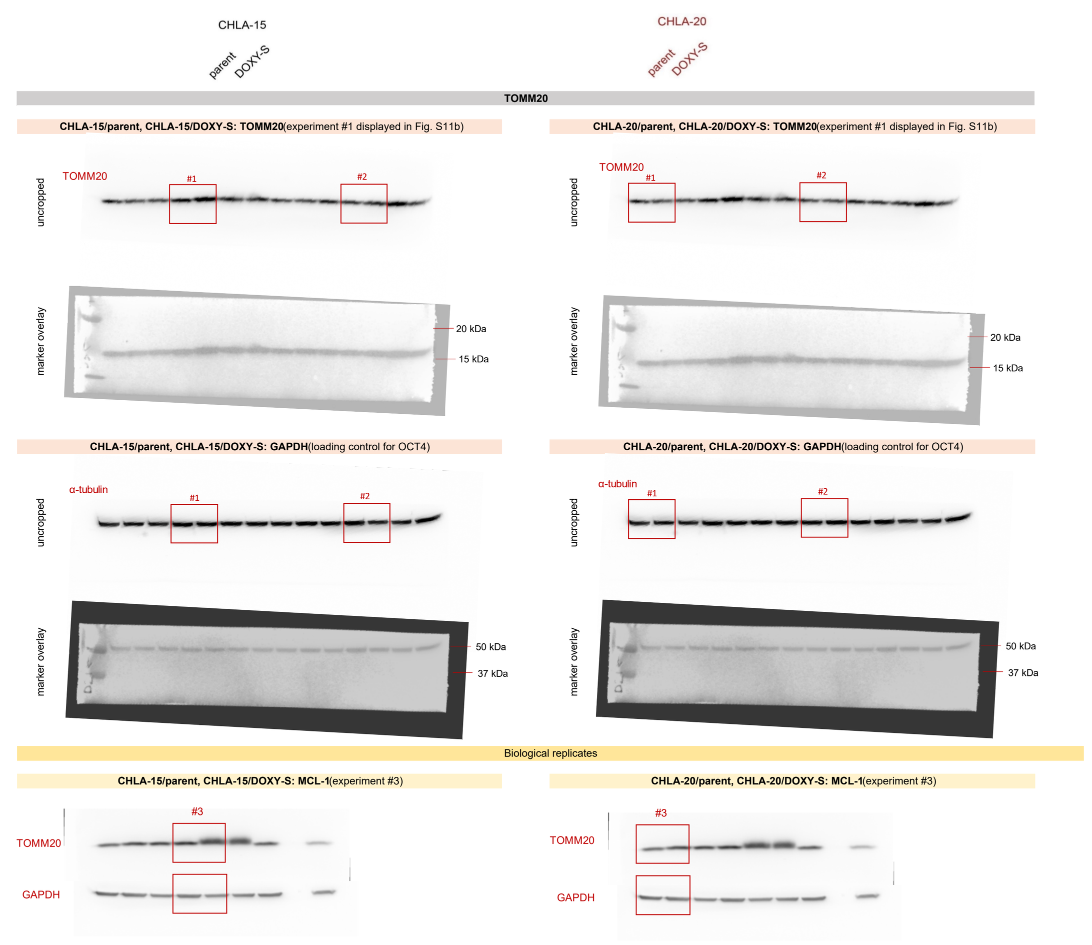

Figure S14a

*Uncropped images of experiments displayed in **Figure S14a** are followed by other biological replicates. Red box indicates lines related to the figure. Sample order same as displayed in the figure if not indicated differently.*

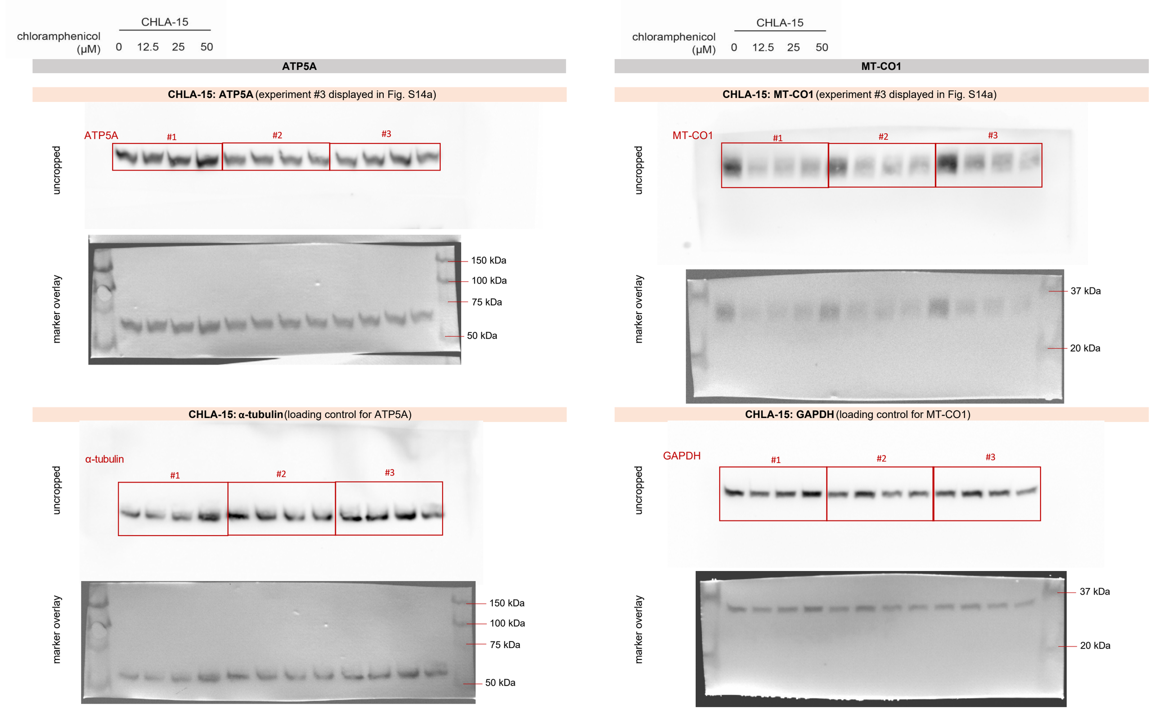

Figure S14b

*Uncropped images of experiments displayed in **Figure S14b** are followed by other biological replicates. Red box indicates lines related to the figure. Sample order same as displayed in the figure if not indicated differently.*

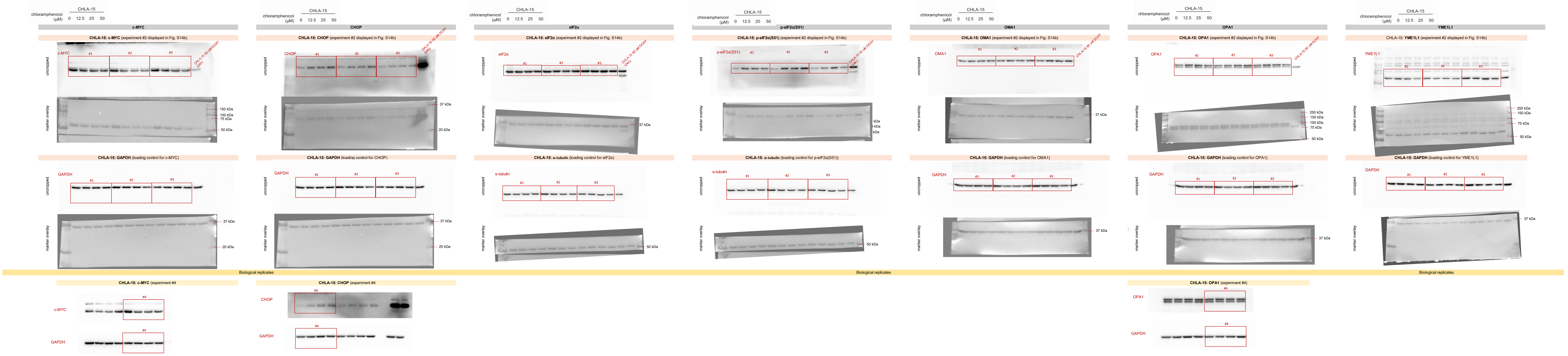

Figure S14c

*Uncropped images of experiments displayed in **Figure S14c** are followed by other biological replicates. Red box indicates lines related to the figure. Sample order same as displayed in the figure if not indicated differently.*

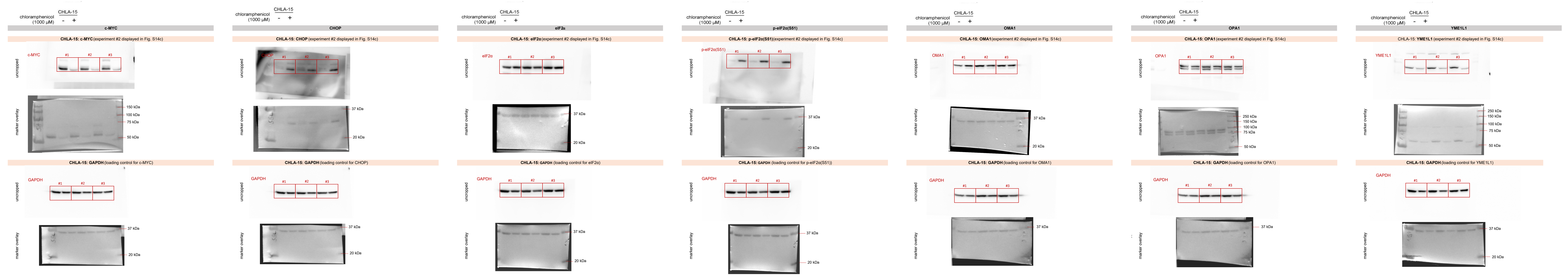

Supplement: Supplementary file 4 — Original Data File [file 41419_2023_6278_MOESM4_ESM.pdf]
